# Characterization of the microbiota dynamics associated with *Moniliophthora roreri*, causal agent of cocoa frosty pod rot disease, reveals new viral species

**DOI:** 10.3389/fmicb.2022.1053562

**Published:** 2023-02-03

**Authors:** Brayan Maudiel Diaz Reyes, Paula Luize Camargos Fonseca, Neander Marcel Heming, Lucas Barbosa de Amorim Conceição, Katiucia Ticila de Souza Nascimento, Karina Peres Gramacho, Enrique Arevalo-Gardini, Carlos Priminho Pirovani, Eric Roberto Guimarães Rocha Aguiar

**Affiliations:** ^1^Departamento de Ciências Biológicas, Universidade Estadual de Santa Cruz, Ilhéus, Brazil; ^2^Departamento de Genética, Instituto de Ciências Biológicas, Universidade Federal de Minas Gerais, Belo Horizonte, Brazil; ^3^Instituto de Biologia, Universidade Federal da Bahia, Salvador, Brazil; ^4^Centro de Pesquisas do Cacau, Comissão Executivo do Plano da Lavoura Cacaueira, CEPEC/CEPLAC, Rodovia Ilhéus-Itabuna, Ilhéus, Brazil; ^5^Instituto de Cultivos Tropicales, Tarapoto, Peru; ^6^Universidad Nacional Autónoma de Alto Amazonas, Yurimaguas, Peru

**Keywords:** *Theobroma cacao*, microbiota, metatranscriptomics, frosty pod rot disease, virus, narnavirus, fungi

## Abstract

**Introduction:**

*Theobroma cacao*, the cocoa tree, is a target for pathogens, such as fungi from the genera *Phytophthora*, *Moniliophthora*, *Colletotrichum*, *Ceratocystis*, among others. Some cacao pathogens are restricted to specific regions of the world, such as the *Cacao swollen shoot virus* (CSSV) in West African countries, while others are expanding geographically, such as *Moniliophthora roreri* in the Americas. *M. roreri* is one of the most threatening cacao pathogens since it directly attacks the cacao pods driving a significant reduction in production, and therefore economic losses. Despite its importance, the knowledge about the microenvironment of this pathogen and the cocoa pods is still poorly characterized.

**Methods:**

Herein we performed RNA sequencing of spores in differential stages of culture in a medium supplemented with cacao pod extract and mycelium collected of the susceptible variety ICT 7121 naturally infected by the pathogen to evaluate the diversity and transcriptional activity of microorganisms associated with the *in vitro* sporulation of *M. roreri*.

**Results:**

Our data revealed a great variety of fungi and bacteria associated with *M. roreri*, with an exceptional diversity of individuals from the genus *Trichoderma* sp. Interestingly, the dynamics of microorganisms from different kingdoms varied proportionally, suggesting they are somehow affected by *M. roreri* culture time. We also identified three sequences similar to viral genomes from the *Narnaviridae* family, posteriorly confirmed by phylogenetic analysis as members of the genus *Narnavirus*. Screening of *M. roreri* public datasets indicated the virus sequences circulating in samples from Ecuador, suggesting a wide spread of these elements. Of note, we did not identify traces of the viral sequences in the *M. roreri* genome or DNA sequencing, restricting the possibility of these sequences representing endogenized elements.

**Discussion:**

To the best of our knowledge, this is the first report of viruses infecting the fungus of the genus *Moniliophthora* and only the third description of viruses that are able to parasite elements from the *Marasmiaceae* family.

## 1. Introduction

*Theobroma cacao L*. (*Malvaceae*) is a tropical plant native to the Amazon, which grows naturally in the shade of tropical forests (Motamayor et al., 2002; Garcia et al., 2018). The genus *Theobroma* is constituted of 22 species, of which, the ones of economic importance are *Theobroma cacao* L. and *Theobroma grandiflorum* as they provide the raw material for chocolate production and other food industries (Bartley, 2005). In addition to many health benefits (Franco et al., 2013), the flavonoids in cocoa enter and build up in the parts of the brain responsible for memory and learning (Shahanas et al., 2019), consuming cocoa-derived products may also activate modifications in redox-sensitive signaling pathways that control gene expression and immunological response (Katz et al., 2011). For this reason, cocoa is classified as one of the most important commodities worldwide responsible for improving the economic livelihoods of farmers (Osorio-Guarín et al., 2020).

Chocolate has great demand and acceptance globally, however, its supply depends on the cultivation of cacao in favorable climatic conditions and low incidence of pests and diseases (Dantas Neto et al., 2005; Evans et al., 2013). Phytopathogenic fungi are one of the primary disease agents that attack cocoa plantations in tropical countries (Dos Santos et al., 2017). One example is frost pod rot (FPR), caused by *Moniliophthora roreri*, a hemibiotrophic basidiomycete belonging to the *Marasmiaceae* family (Aime and Phillips-Mora, 2005). This species is usually found infecting fruits of the plant genera *Herrania* and *Theobroma* (Bailey et al., 2018), impacting especially cocoa beans that can lead to yield losses of up to 80% (Hidalgo et al., 2003; Sánchez et al., 2012). In South American countries, the witches’ broom disease of cacao (WBD) caused by the fungi *Moniliophthora perniciosa* figure as one of the major threats to cocoa production that results in significant yield and economic losses (Marelli et al., 2009; Teixeira et al., 2015), ranging from 50 to 90% in affected regions (Meinhardt et al., 2008). Indeed, WBD has caused significant crop losses, especially in Brazil, where the yield of cacao dropped by more than 70% in the 10 years after the disease first appeared (Teixeira et al., 2014).

The origin of *M. roreri* occurred in Colombia, and then migrated to Ecuador and spread to other regions, such as Venezuela, Peru, and Central American countries (Phillips-Mora et al., 2007). The last report of the presence of the disease in an invasive profile had been in Jamaica (Johnson et al., 2017). Of note, the first outbreak of the disease in Brazil was recently described in the city of Cruzeiro do Sul (Acre state), with symptomatic plants identified, and, posteriorly, the presence of the fungi pathogen validated by the Federal Laboratory for Agricultural Defense – Goiânia (AGROLINK, 2021).

Other fungal pathogens also cause losses in cocoa. For example, the species *Phytophthora megakarya* in West Africa presents a threat to the crop in other regions due to its high virulence (Marelli et al., 2019). The fungus *Ceratocystis cacaofunesta* is one of the most aggressive pathogens impacting cocoa production in different countries, leading to the destruction of large numbers of *Theobroma* crops (Mora-Ocampo et al., 2021). Anthracnose, a disease caused by *Colletotrichum* spp., is a limiting factor in production and the causal agent of this disease has been reported in different countries, as in Ghana an anthracnose outbreak covered a cultivated area of 248.47 hectares, leading to losses (Asare et al., 2021), or in Brazil, in which *Colletotrichum aeschynomenes* was identified in October 2016 colonizing cocoa plants (Nascimento et al., 2019). Moreover, many cocoa-producing countries in West Africa, have been infected by viral diseases such as cocoa swollen stem disease (CSSD) caused by the *Cocoa swollen shoot virus* (CSSV; Abrokwah et al., 2016).

Due to the number of pathogens described infecting *T. cacao*, many studies have proposed to investigate the microbiota associated with the infected plant as well as to assess the potential of endophytes as agents for biological control of these pathogenic agents. A previous study in the Equatorial Amazon characterized the microbiota of cacao plants based on morphological criteria identifying several species from the genera *Cyliindrocladium*, *Dichobotrys*, *Moniliophthora*, *Colletotrichum*, and *Phytophthora* (Carrera-Sánchez et al., 2016). In addition, a study using morphological characteristics and ribosomal DNA sequencing focused on the branch part of cacao plants also revealed a great diversity of endophytic fungi by including the presence of species from the genera *Acremonium*, *Blastomyces*, *Botryosphaeria*, *Cladosporium*, *Colletotrichum*, *Cordyceps*, *Diaporthe*, *Fusarium, Geotrichum, Gibberella, Gliocladium, Lasiodiplodia, Monilochoetes, Nectria, Pestalotiopsis, Phomopsis, Pleurotus, Pseudofusarium, Rhizopycnis, Syncephalastrum, Trichoderma, Verticillium*, and *Xylaria* (Rubini et al., 2005). Several endophyte organisms were recorded directly in the field of healthy tree trunks and pods, and more than 40 genera were identified and recorded mainly representing anamorphs of Hypocreales in the genera *Acremonium*, *Clonostachys*, and *Trichoderma* (Evans et al., 2003).

The advent of High-Throughput Sequencing (HTS) and RNA sequencing (RNA-seq) technologies have revolutionized the study of transcriptionally active elements and enabled the assessment of hidden microbial diversity in terms of different environmental parameters (Colgan et al., 2017; Saminathan et al., 2018). *Omics* sciences have provided a more detailed view of microbial interactions (Aguiar-Pulido et al., 2016). For instance, metatranscriptomics allows access to both transcriptional active community members and the mapping of metabolic pathways (Urich et al., 2008). Moreover, the *Omics* studies have revealed important information about microbial diversity, and interpreting these interactions is fundamental for developing sustainable agricultural practices (Priya et al., 2021).

In our study, RNA deep sequencing was applied to identify and characterize the microbiota dynamics associated with spores of *M. roreri* in differential culture times and mycelium using medium supplemented with cacao extract collected from plants naturally infected by the pathogen. Our metagenomics approach revealed higher diversity of species related to fungal and bacterial kingdoms, in special to *Trichoderma* fungi that have been described with potential for biological control of the different diseases affecting the crop. Unexpectedly, we also identified viral species belonging to the *Narnaviridae* family likely infecting *M. roreri*. This is the first report of viral infection in this species. Altogether, our results provided important data on the understanding of microorganism dynamics during frosty pod rot infection in *T. cacao* and revealed new species that can play a role in fungi-plant interactions.

## 2. Materials and methods

### 2.1. Spore and mycelium production

The spores and vegetative mycelium of *Moniliophthora roreri* were obtained from naturally infected cocoa fruits of the susceptible cocoa variety ICT 7121 at the *Instituto de Cultivos Tropicales* (ICT) located in Tarapoto City, Peru in May 2015 through a partnership between Brazilian and Peruvian institutions. Four-month-old diseased cocoa pods affected by frosty pod rot disease, showing necrotic spots and white pseudostroma were harvested, washed with running water using a brush to eliminate the mycelium, and then immersed in a 2% sodium hypochlorite solution for 5 min. Then, they were dried with a paper towel, cut into two-cm thick slices, transversal to the longitudinal axis of the fruit, placed on the surface of Petri dishes, and kept in a humid chamber for three to 4 days at 21–25°C for growth of the vegetative mycelium (Supplementary Figures 1A, B). After cultivation, the mycelium was carefully removed by scraping the surface of the slices with the aid of a sterile scalpel (Supplementary Figure 1C). The mycelial mass was washed twice with 10% Trichloroacetic Acid (TCA) in Acetone. Two milliliters of RNAlater were then added to the dried pellet for further extraction of total RNA. An aliquot of this material was inoculated into a medium, as well as a positive control, in order to show that the chemical treatments performed made the spores non-viable. The fresh spores were inoculated on a medium containing cocoa broth after incubating the cocoa fruit slices for 5–8 days (Supplementary Figure 1D). For the preparation of the culture medium, 250 g of healthy susceptible variety ICT 7121 cocoa fruit were used, autoclaved in 1 L of distilled water. Then, the broth was strained, and 0.15% agar was added. After cooling, the cocoa agar medium was plated on Petri dishes. A total of 20 Petri dishes were used to inoculate 1 ml of *M. roreri* spore suspension at a concentration of 4.2 × 10^6^ spores/ML for the times 8, 16, 24, and 48 h after inoculation (hai). The ungerminated dry spores were collected using a fine brush and a beaker with 100 ml of autoclaved water containing streptomycin at a concentration of 0.01%. This solution was homogenized with 0.01% Tween 80, then filtered through sterile gauze. The spores were counted in a Neubauer chamber. Then, the suspension was centrifuged to obtain the spore mass, which was washed in (TCA) 10% in Acetone twice, added 2 ml of RNAlater (Fisher Scientific, United States) to the dried pellet for further extraction of total RNA.

### 2.2. Total RNA extraction and quantification

After removal of the RNAlater, the dried inactive mycelium and spore pellets at inoculated times 0 (uninoculated), 8, 16, 24, and 48 hai were macerated using liquid nitrogen. For each sample, dry weights were obtained, and the samples were stored at -80°C. Total RNA was extracted with the ZR Plant RNA MiniPrepTM kit (Zymo Research, United States) following the manufacturer’s instructions. Total RNA was quantified by Qubit fluorimeter (Invitrogen, United States) using Qubit® dsRNA HS/BR kits and Nanodrop 2000c (Thermo Scientific, United States; Supplementary Table 1).

### 2.3. Library construction and sequencing

The messenger RNA (mRNA) libraries were constructed using the TruSeq RNA® v2 Low Sample (LS) kit (Illumina, United States) according to the manufacturer’s instructions. The purity level and the size of the fragments obtained from the mRNA libraries of the spores at different germination times (0, 8, 16, and 48 hai) and of the mycelium were checked on agarose gel 3%, using DNA markers of 50 bp (New England/Biolabs, United States), 100 bp (Gene Ruler/Fermentas, United States). Absolute quantification of the libraries was performed using the Kapa Library Quantification ABI Prism® qPCR Mix kit (Kapa Biosystems, United States) according to the manufacturer’s instructions. A pool of the samples at a concentration of 15 pM and 5% of the PhiX control at the same concentration was used for each sequencing run. The sequencing was outlined as follows: (i) sequencing only spores time 0 h after inoculation; (ii) sequencing mycelium and spore pool 48 hai; (iii) sequencing spore pool with 8 hai and spores with 16 hai, only one sequencing kit for each one of these outlines. The sequencing was performed in the MiSeq System equipment (Illumina, United States) located in the Center of Biotechnology and Genetics/UESC (CBG) using the MiSeq® Reagent Kit v3 (Illumina, United States) of 150 cycles. The libraries produced in our study were deposited in the NCBI SRA database under Project accession number: PRJNA854689.

### 2.4. Library processing

The obtained raw reads (reads size ranging from 35 to 76 bp) were subjected to a pre-processing step. Initially, reads with quality equal to or greater than Phred 30 and size greater than 20 bp were selected with the Trimmomatic 0.33 tool (Bolger et al., 2014). The remaining reads were used in a quality analysis with the FASTQC tool (Andrews, 2017). The filtered sequences were then mapped against the reference genomes of the plant *T. cacao* and the fungus *M. roreri* using the Bowtie2 tool (Langmead and Salzberg, 2012) to remove sequences from these organisms. The unmapped reads were used for the subsequent analyses. The reference genomes of *Theobroma cacao* and *Moniliophthora roreri* were downloaded from the NCBI GenBank database using the accession numbers GCA_000403535 and GCA_000488995.1, respectively. All analyses were performed using the Galaxy Bioinformatics platform (Afgan et al., 2018).^1^ The pre-processed reads were used in the subsequent analyses.

### 2.5. Metagenomics analysis

The pre-processed reads were used for contig assembly with the Trinity tool [Trinity *de novo* assembly of RNA-Seq data (Galaxy version 2.9.1)] using default parameters (Grabherr et al., 2011). The assembled contigs were evaluated on the Kaiju web server^2^ with the parameters (e-value <0.001) by searching the NCBI BLAST nr + euk − non-redundant protein database. Libraries that showed viral hits were subjected to sequence similarity analysis with Diamond (Buchfink et al., 2015) which returned multiple fragments for each library per condition that were subsequently reassembled with the CAP3 tool (Huang and Madan, 1999). The analysis of abundance at kingdom, genera, and species level was based on taxonomical classification by Kaiju which only elements showing abundance equal or greater than five were considered. Transcripts assigned to the species *M. roreri* and *M. perniciosa* were also removed from these analyzes. Species and genera with less than five transcripts are listed in Supplementary Data 1. The viral sequences identified in our study were deposited in NCBI GenBank database under the accession numbers: ON210269, ON210270, and ON210271.

### 2.6. Temporal analyses of beta diversity

In order to analyze changes in the assemblage composition between different times we calculated temporal beta diversity (Baselga et al., 2015) using the function “beta.temp” from “betapart” R package (Baselga et al., 2021) in program R (R Core Team, 2021). Temporal beta diversity uses a similar concept of spatial beta diversity, allowing the partitioning of beta diversity into turnover and nestedness components. However, instead of assessing how composition changes across a set of sites, it assesses changes on each site between different times (Baselga, 2010; Baselga et al., 2015). Because we have five time points (spores 0, 8, 16, 48 hai, and Mycelium), we calculated beta-diversity between each time and its subsequent time, which allowed us to compare the composition of assemblages across all pairs of successive times of infection. Beta diversity was calculated using Jaccard dissimilarity.

### 2.7. Phylogenetic analysis

A dataset containing the viral contigs assembled in our study and public protein sequences related to *Mitovirus*, *Narnavirus*, and *Ourmiavirus* was constructed and aligned using the MAFFT program (Katoh et al., 2019). The best evolutionary protein model was selected using ModelTest, considering Akaike’s information criterion (Akaike, 1998). Maximum likelihood inference was built in IqTree using 1,000 bootstrap replicates (Nguyen et al., 2015). The tree was rooted considering *Escherichia virus* as an external group (accession number: NP_040755) and edited in Figtree.^3^

### 2.8. Analyses of RNA and DNA sequencing public data from *Moniliophthora roreri*

Three publicly available deep sequencing libraries from *M. roreri* were downloaded from the SRA database. Two RNA-seq libraries (SRR1036616 and SRR1034656) contained mixed pathogen-infected plant material (*M. roreri* and *T. cacao*), derived from an *M. roreri* clone (MCA2977) isolated from the state of Los Rios, Ecuador (Meinhardt et al., 2014). The third library (SRR8453395), prepared from genomic DNA, was derived from *M. roreri* CPMRT01 isolate identified in *T. cacao* plants in Tabasco, Mexico (Hipólito-Romero et al., 2020). All three libraries were used to investigate the presence and abundance of viral sequences detected in our library using the Kallisto tool (Bray et al., 2016).

## 3. Results

### 3.1. *De novo* transcriptome assembly

The total RNA sequencing produced over 85 million reads distributed among the five libraries (conditions). The number of raw reads ranged from 12,384,924 in the mycelium condition to 25,840,512 in spores 0 hai (uninoculated; Supplementary Table 1). After pre-processing, more than 99% of the sequences were kept, supporting the quality of our RNA deep sequencing. Pre-processed sequences were submitted to *de novo* assembly with Trinity, producing a total of 169,074 transcripts. The number of transcripts assembled ranged from 20,879 in Mycelium to 65,605 in spores 48 hai, with mean varying from 508 to 780 nt and N50 between 604 to 1,088 nt (Supplementary Table 1).

### 3.2. Diversity of bacteria and fungi associated with *Moniliophthora roreri* infection in cocoa pods

**Figure 1:**
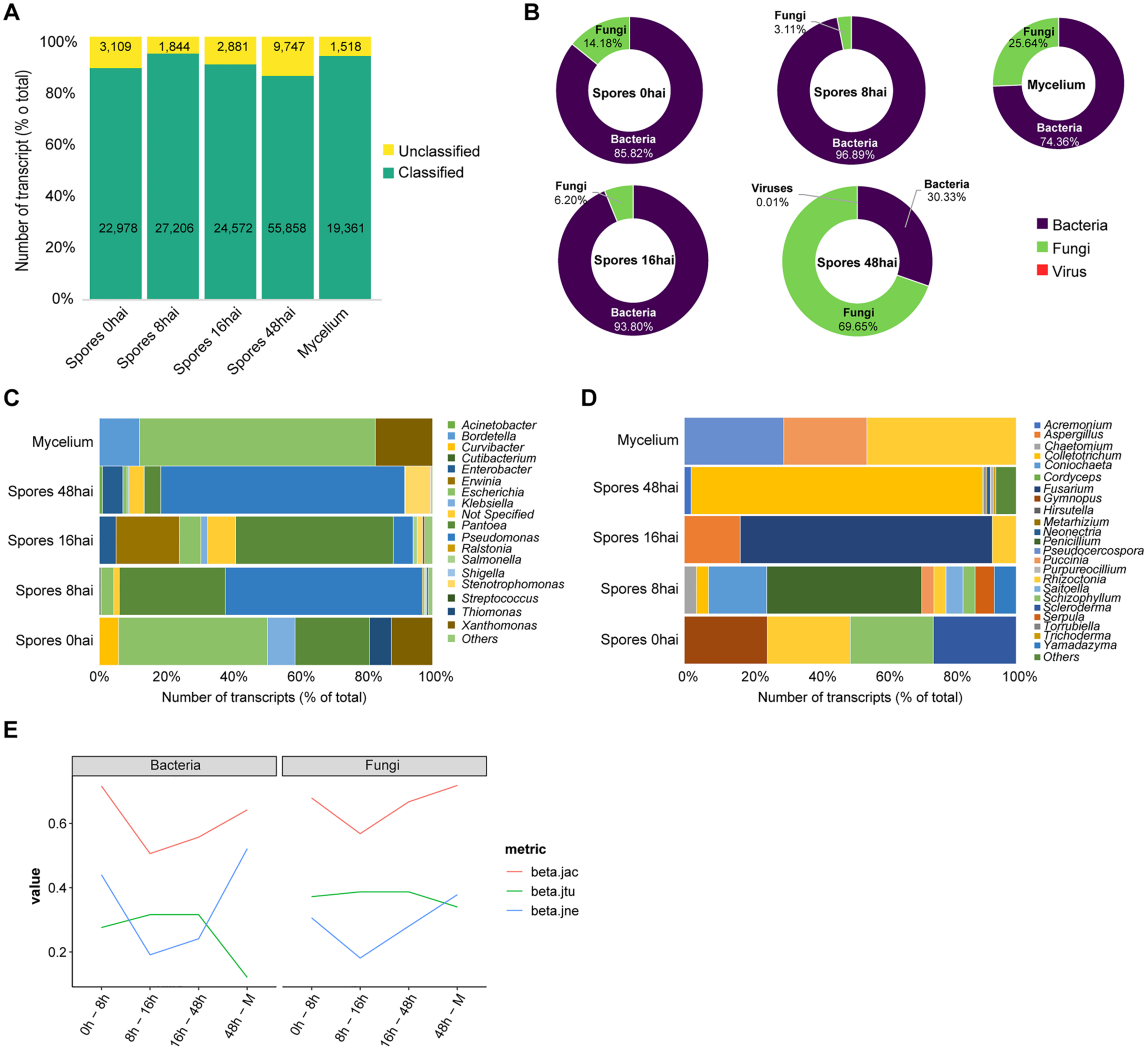
Metagenomics analysis of *Theobroma cacao* pods infected by *Moniliophthora roreri*. **(A)** Overview of taxonomic assignment of assembled transcripts by condition. **(B)** Abundance of fungi, bacteria and virus in the samples. Diversity of bacteria **(C)** and fungi **(D)** by genera. **(E)** Temporal changes in microbiota of *T. cacao* fruits along *M. roreri* infection time points. beta.jtu, beta.jne and beta.jac, represents the partitioning of beta diversity into turnover, nestedness and jackniffe, respectively. hai: hours after inoculation.

The assembled transcripts were classified using sequence-similarity searches according to their closest relative in public databases. From the total 169,074 (100%) transcripts, 149,975 (88.17%) were classified at least at Kingdom level while 19,099 (11.24%) were unassigned. We observed the highest percentage of classified transcripts in the library of spores 8 hai. Library derived from Spores 48 hai showed the highest number of transcripts without taxonomic assignment (Figure 1A).

#### 3.2.1. Bacterial diversity

From the 169,074 transcripts that did not match *Moniliophthora* species, 15,365 sequences could be assigned to bacterial species distributed in the libraries: spores 0 hai (121 transcripts), spores 8 hai (4,270 transcripts), spores 16 hai (1,255 transcripts), spores 48 hai (9,661 transcripts), and mycelium (58 transcripts; Figure 1B). The genus *Pseudomonas* had the highest number of elements assigned in all sequenced libraries with a median of 1933.8 transcripts per library (Figure 1C). *Pseudomonas* is one of the most ubiquitous bacterial genera and has been isolated worldwide from different environments, such as clinical, plants, fungi, and animal samples (Peix et al., 2009). Within *Pseudomonas* genera, the most abundant species were *Pseudomonas parafulva* (2,531 transcripts), *Pseudomonas* sp. *Bc-h* (1,188 transcripts)*, Pseudomonas* sp. *NFR16* (1,019 transcripts), *Pseudomonas putida* (928 transcripts), and *Pseudomonas abietaniphila* (878 transcripts). Other species that ranked in the top 10 were *Pantoea dispersa* (1,761 transcripts), *Stenotrophomonas maltophilia* (671 transcripts), *Enterobacter cancerogenus* (627 transcripts), *Escherichia coli* (456 transcripts), and *Type-E symbiont of Plautia stali* (431 transcripts).

Other genera also showed a large number of transcripts, such as *Pantoea* (2,453 transcripts), *Enterobacter* (663 transcripts), *Acinetobacter* (100 transcripts), *Stenotrophomonas* (745 transcripts), *Escherichia* (456 transcripts), *Erwinia* (250 transcripts), *Klebsiella* (86 transcripts), *Xanthomonas* (63 transcripts), and *Salmonella* (54 transcripts). The genus *Escherichia* was present in all five libraries. The *Pantoea and Xanthomonas* are present in four libraries, and *Pseudomonas* are present in three of the Spore conditions (Figure 1C). We noticed a clear change in the bacterial community between Spores and Mycelium, with some genera being condition-specific while others showed high prevalence among all conditions. Four different strains of the bacteria *Pantoea dispersa*, which had 1,761 transcripts assigned, have been described as inhibiting the mycelium growth of the fungus *Ceratocytis fimbriata* causing black rot in sweet potato, spore germination, as well as altering the morphology of fungal hyphae thus having biological control potential (Jiang et al., 2019).

#### 3.2.2. Fungal diversity

Fungi was the most represented kingdom identified in cocoa pod microbiota. A total of 22,444 (68.05%) transcripts distributed between spores 0 hai (20), spores 8 hai (137), spores 16 hai (83), spores 48 hai (22,184), and mycelium (20) conditions were identified (Figure 1D). Analyzing the diversity at the genera level, we can highlight *Colletotrichum* (1924 transcripts), *Fusarium* (699 transcripts), *Acremonium* (440 transcripts), *Neonectria* (229 transcripts), *Purpureocillium* (129 transcripts), *Trichoderma* (129 transcripts), *Metarhizium* (102 transcripts), *Hirsutella* (89 transcripts), *Cordyceps* (88 transcripts), and *Torrubiella* (86 transcripts). Regarding fungi abundance, we note an unexpected abundance of transcripts derived from *Colletotrichum* elements in the library from Spores 48 hai. Some other genera, such as *Rhizoctonia*, were identified in three libraries (pores 0, 8, and 16 hai). On the other hand, many genera were represented only in specific conditions, such as *Scleroderma* (spores 0 hai), and *Yamadazyma* (spores 8 hai; Figure 1D).

At the species level, the most abundant fungal species was *Colletotrichum gloeosporioides* with 18,116 transcripts, followed by *Acremonium chrysogenum* with 440. We also noticed the presence of many species from the genus *Trichoderma*. Indeed, the species *Trichoderma virens* was present in all libraries while the library constructed from spores 48 hai showed the highest diversity of members from this genus, presenting transcripts from *T. reesei, T. parareesei, T. harzianum, T. guizhouense, T. gamsii*, and *T. atrovirie* species. In the same libraries, we also identified sequences related to the species *Beauveria bassiana* (11 assigned transcripts), another species from the *Hypocreales* order that is also considered endophytic.

### 3.3. Temporal changes in fungal and bacterial diversity

In order to assess the changes in microbiota composition in *T. cacao* pods according to *M. roreri* time of inoculation, we calculated the changes of beta diversity along the different times of infection. We observed higher changes in spores 0 hai compared to spores 8 hai and spores 48 hai compared to Mycelium (Figure 1E). This difference is mainly driven by richness observed in spores 8 hai and spores 48 hai which is at least two times higher than the other conditions (Supplementary Table 2). Interestingly, in most cases, with few exceptions (bacterial species from spores 0 hai to spores 8 hai and spores 48 hai to mycelium and fungal species from spores 48 hai to mycelium) the changes in the community structure were dominated by species turnover (i.e., species replacement were larger than the loss/gain of species; Supplementary Tables 3, 4 and Supplementary Data 1). This profile is highlighted in the comparison between spores 0 hai and mycelium, where the ratio of beta-diversity (turnover/nestedness) is 0.79 and 0.89 for bacterial and fungal species, respectively. Of note, we observed that the changes observed were very similar for fungi and bacteria, showing a strong Pearson correlation (*r*: 0.95 *p*: 0.00003). This result suggests these species are somehow similarly affected by *M. roreri* time of infection.

### 3.4. Characterization of viral sequences associated to *Moniliophthora roreri*

Our metagenomics analyses using Kaiju’s webserver identified four contigs showing similarity to viral sequences in the library constructed from spores 48 hai. Two contigs were closely related to the viral species *Ophiostoma mitovirus 5*, that according to the International Committee on Taxonomy of Viruses (ICTV) belongs to the genus *Mitovirus* – *Mitoviridae* (Lefkowitz et al., 2018). We also identified one contig related to the *Sanxia narna-like virus 1*, which is an unclassified RNA virus (Schoch et al., 2020). Finally, one contig presented similarity to Pseudomonas phage PPpW-3, a DNA virus associated with bacteria from the family *Myoviridae*.

**Figure 2:**
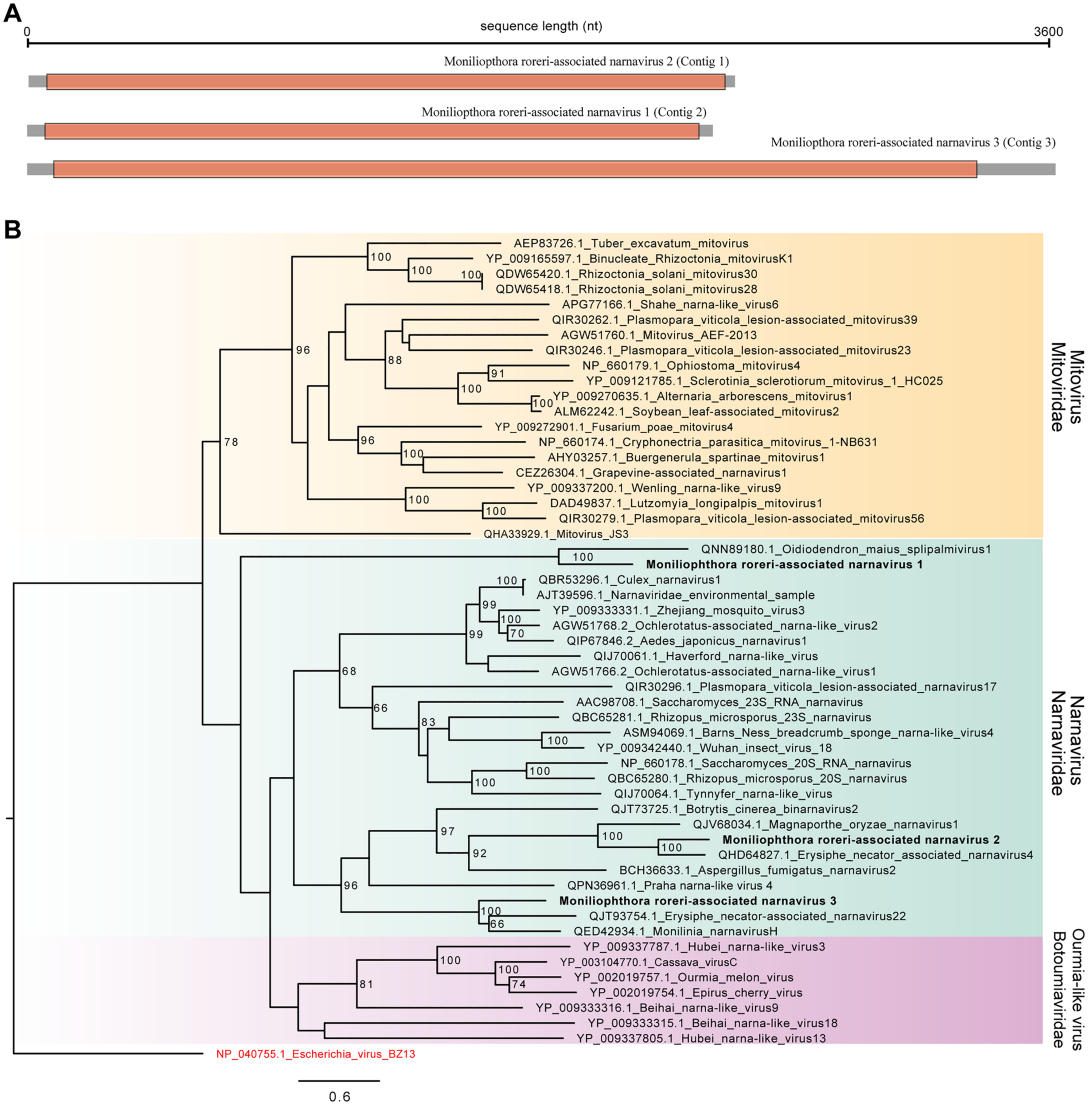
Characterizations of *Moniliophthora roreri*-associated viral sequences. **(A)** Genomic structure of M. roreri associated viruses. Gray square represents the whole viral segment while orange region indicates the large Open Read Frame. **(B)** Maximum likelihood tree constructed based on large viral ORF translated into protein. Viral sequences identified in the work are highlighted in bold. Bootstrap values larger than 60 are indicated in the tree.

Since we had indicative signs of viral presence in the samples, we performed an extra step of virus identification using sequence similarity searches against the NR database using the Diamond tool. Using this strategy, we detected 30 transcripts showing similarity to viral sequences related to species from the *Narnaviridae* family, genus *Narnavirus* (Supplementary Table 5). After redundancy removal and contig extension step, three viral sequences were kept, Contig 1 (2,460 nt), Contig 2 (2,332 nt) and Contig 3 (3,606 nt). These sequences were further validated by BLASTx searches at NCBI website to guarantee the most updated version of sequence databases (Supplementary Tables 6–8; Altschul et al., 1997). Contigs 1, 2 and 3 showed similarity to *Erysiphe necator associated narnavirus 4* (QHD64827.1), *Magnaporthe oryzae narnavirus 1* (BCH36655.1), and *Monilinia narnavirus H* (QED42934.1), respectively. Structural annotation revealed that Contig 1 encodes to an open reading frame of 788 amino acids, Contig 2 (754 amino acids), and Contig 3 (1,085 amino acids; Figure 2A). Of note, the search for domains did not reveal any conserved region for any of the three putative viral genomes, which is common for elements from *Narnavirus* genus.

### 3.5. Phylogenetic characterization of *Moniliophthora roreri*-associated viral sequences

In order to further characterize the putative viral sequences identified in our study, we performed phylogenetic analyses with closely related sequences present in public databases and related families indicated by ICTV, *Mitoviridae* and *Botoumiaviridae*. As observed in sequence similarity searches, the three viral transcripts clustered with different species of the *Narnaviridae* family. Contig 1 clustered with the species *Erysiphe necator narnavirus 4* (QHD64827), while Contig 2 grouped with the species *Oidiodendron maius splipalmivirus 1* (QNN89180), both showing 100 bootstrap replicates. Contig 3 formed a cluster with the species *Erysiphe necator narnavirus 22* (QJT93754) and *Monilinia narnavirus H* (QED42934). According to our phylogenetic analysis, the contigs identified in our study represent new viral genomes related to the *Narnaviridae* family, specifically from *Narnavirus* genus. They were named *Moniliophthora roreri-associated narnavirus* 1 (Contig 2), *Moniliophthora roreri-associated narnavirus 2* (Contig 1) and *Moniliophthora roreri-associated narnavirus 3* (Contig 3) to reflect host origin (Figure 2B).

### 3.6. Presence of *Moniliophthora roreri*-associated viruses in public data

**Figure 3:**
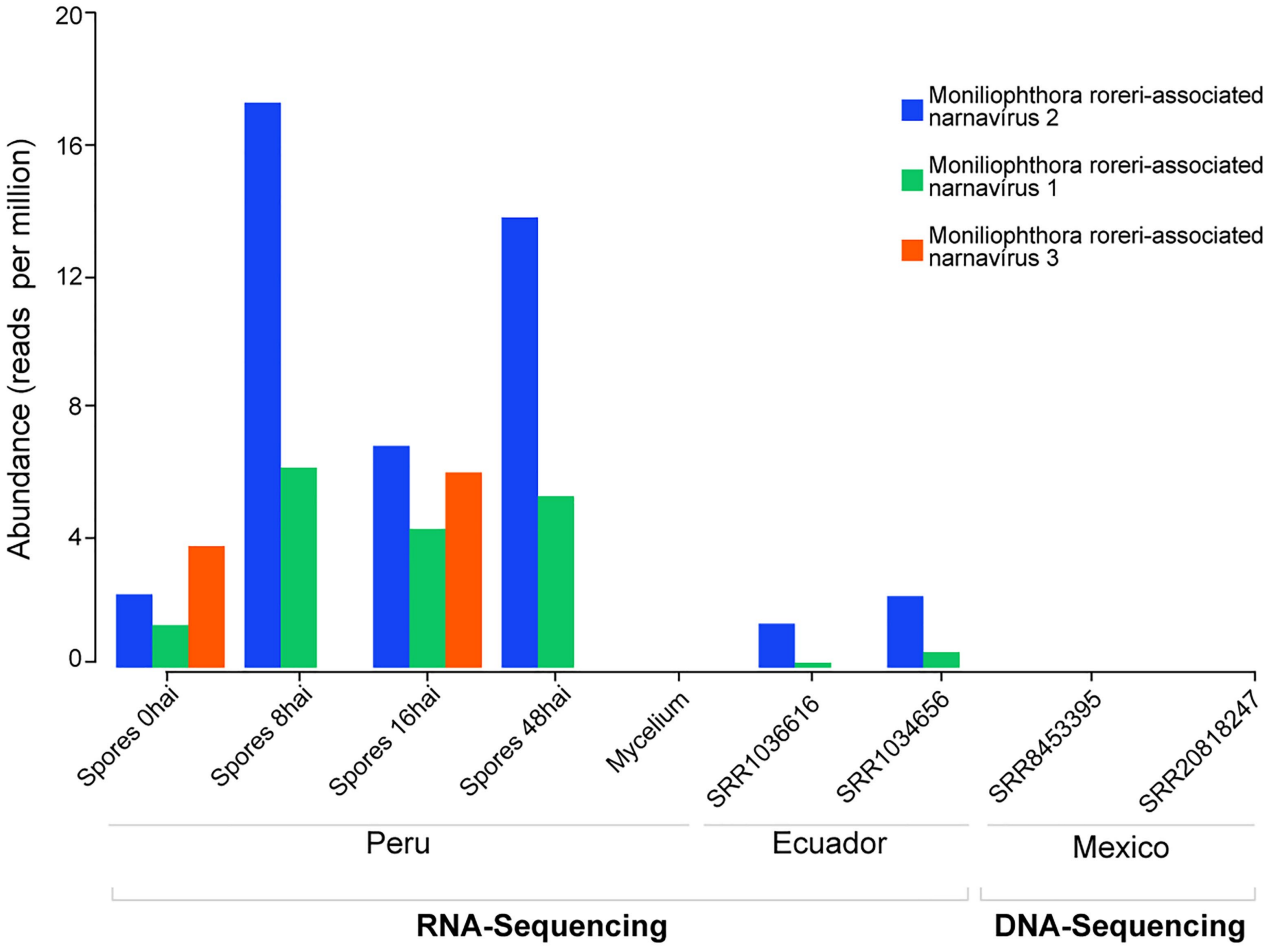
Presence of *Moniliophthora roreri*-associated viral sequences in public datasets. RNA and DNA deep sequencing libraries deposited at NCBI SRA databases from samples originated in different countries were investigated regarding the presence of reads derived from *M. roreri*-associated viral sequences.

To evaluate the presence of the viral sequences found in the cocoa pods affected by frosty pod rot disease sequenced in our study, we investigated publicly available RNA deep-sequenced libraries derived from *M. roreri*. We were able to identify the presence of the Moniliophthora roreri-associated narnavirus 2 and Moniliophthora roreri-associated narnavirus 1 in two libraries from Los Rios, Ecuador submitted by the USDA, containing mixed material from 30 days and 60 days after inoculation of *T. cacao* pods with the *M. roreri* (Figure 3).

To rule out the possibility of these sequences representing viral elements integrated into the genome of *M. roreri* we also searched fragments of *M. roreri*-associated viruses in the fungi genome and DNA sequencing libraries. As expected for RNA viruses that do not produce DNA intermediates in their replication cycle, we were not able to find signals of the viral sequences in the *M. roreri* genome or genomic libraries (Figure 3).

Since *M. roreri* and *M. perniciosa* are related and often occur in the same region, we also assessed the possible presence of the *M. roreri*-associated viruses in public RNA deep-sequenced libraries derived from the sister taxon. Therefore, we searched for the viruses in 10 samples of healthy seedlings and seedlings 30 days after *M. perniciosa* infection (Teixeira et al., 2014). However, we were not able to identify positive hits for the *M. roreri*-associated viruses identified in our study.

## 4. Discussion

The cacao tree (*Theobroma cacao* L.) is a crop of economic importance to many countries in the world. Nevertheless, its production and supply of almonds, which is the main source for chocolate manufacturing, has been hampered by unfavorable weather conditions (Wessel and Quist-Wessel, 2015) and pathogen attacks leading to economic losses and plantation abandonment. Therefore, an effort is necessary to address the problems associated with the control and management of different diseases that affect cocoa production to ensure the economic sustainability of producers (Adeniyi, 2019). Fungal diseases, and *Moniliophthora* species, in particular, remain a major constraint on cocoa production in the Americas and, if they exceed their current range, threaten to seriously damage the chocolate industry worldwide (McElroy et al., 2018), as in Panama, attacks by *M. roreri* and *Phytophthora* sp. cause losses of up to 80% of pods, affecting production (Krauss et al., 2006).

In our study, we performed an exploratory study to investigate the microbiota of spores at different times after inoculation and the mycelium of *M. roreri* using a RNA-seq approach. Our data revealed abundance and diversity of many fungal genera, such as *Fusarium, Acremonium, Neonectria, Purpureocillium, Trichoderma, Metarhizium, Hirsutella, Cordyceps, Torrubiella*, among others. Moreover, the bacterial genera with higher abundance in our study were *Pseudomonas, Pantoea, Stenotrophomonas, Enterobacter, Escherichia, Erwinia, Acenitobacter, Klebsiella, Xanthomonas*, *Salmonella* and others. A close look at the taxonomic profile of cocoa pods community also revealed the presence of important species such as those of the genus *Colletotrichum*, a genus that is composed of elements with an economic impact on crops, in addition to its importance as a model for the study the evolution of host specificity, speciation and reproductive behaviors (Baroncelli et al., 2016). The results found in our study were similar to other metagenomic studies published in the literature, for example, the study conducted by (Carrera-Sánchez et al., 2016), in which plant material infected by *M. roreri* was collected to analyze the microbiota associated with the disease. The results showed the presence of the genera *Cylindrocladium*, *Dichobotrys*, *Moniliophthora*, *Colletotrichum*, and *Phytophthora*. Another study focused on the assessment of cacao rhizosphere through metatranscriptomics revealed rhizobacteria being plant-growth promoters under drought conditions (Tamchek et al., 2019). Metagenomic analysis of the cocoa bean fermentation microbiome also identified a high percentage of bacteria, yeasts, and bacteriophages from the cocoa beans microbiome. The species *Lactobacillus*, *Gluconacetobacter*, *Acetobacter*, and *Gluconobacter* presented a greater dominance (Agyirifo et al., 2019). Another metagenomic sequencing in fermenting cacao pods revealed 97 genera, with *Acetobacter*, *Komagataeibacter*, *Limosilactobacillus*, *Liquorilactobacillus*, *Lactiplantibacillus*, *Leuconostoc*, *Paucilactobacillus*, *Hanseniaspora*, and *Saccharomyces* dominating (Verce et al., 2021). Endophytic bacterial species such as *Pseudomonas aeruginosa*, *Chryseobacterium proteolyticum* were isolated from healthy cocoa tissues, such as leaves and fruits, and both species showed a reduction of pod injury in disease caused by the fungal pathogen *Phytophthora palmivora* (Alsultan et al., 2019).

Cocoa pods harbor a diversity of microorganisms, some of them involved in the fermentation process such as species of the genus acetobacter, *Komagataeibacter, Erwinia, Pantoea, Tatumella* (Garcia-Armisen et al., 2010; Verce et al., 2021) and lactic acid bacteria such as *Lactobacillus* present in the cocoa fermentation microbiome with diverse functions (Serra et al., 2019). Other bacterial species can act as biological controllers of plant pathogens such as *Pseudomonas* spp., although the ecology of these populations is not fully understood (McSpadden Gardener, 2007), species of the genus *Streptomyces* are known to live endophytically in cocoa pods and seeds, promoting growth and favoring plant health (Tchinda et al., 2016).

A plentiful source of microorganisms that can, both directly and indirectly, support plant growth, defense, and development is found in the endophytic niches of plants (Hanada et al., 2010). Endophytic bacteria colonize the roots of cocoa trees efficiently and these species have promising results in inhibiting fungal growth (Alves-Júnior et al., 2021). Endophytic fungi develop most or all of their life cycle colonizing the host plant tissues, without causing evident damage, and some have benefits against the attack of phytopathogens (Sánchez-Fernández et al., 2013). Endophytic fungi with their wide range of biodiversity have provided useful insights for better knowledge of plant-fungi interaction and their role in host-associated microbiomes (Christian et al., 2015). Indeed, plants interact with a wide variety of fungal endophytes that reside in different tissues, sometimes contributing to plant growth and/or defense against biotic and abiotic stress (Gange et al., 2019). In healthy cocoa pods, 25 endophytic fungi were isolated and characterized morphologically with their reproductive structures. Furthermore, tests *in vitro* and *in vivo* using the endophytic fungi *Aspergillus*, *Fusarium*, and *Ramichloridium*, presented activity against *P. palmivora* in double culture, pod and seedling assays (Simamora et al., 2021). In our study the genera *Fusarium* were among the 10 most abundant, presenting many species that could play a role as biological controllers in *T. cacao*.

Necrotrophic, hemibiotrophic, latent or quiescent, and endophytic are general descriptions of the life modes of *Colletotrichum* species, with hemibiotrophic being the most prevalent (De Silva et al., 2017). The specie *C. gloeosporioides* can be considered endophytic and phytopathogenic. This specie mainly attacks young and soft cocoa leaves causing brown lesions surrounded by a characteristic light-yellow halo (Maximova et al., 2006). Anthracnose is a disease caused by *Colletotrichum* sp. that leads to yield reduction in many crops worldwide (da Silva et al., 2020). The genus is considered the eighth most important group of phytopathogenic fungi in the world, based on scientific and economic insights (Dean et al., 2012). Recently, there was a detection of *C. gloeosporioides* in native cashew species in Brazil (dos Santos et al., 2019). Two new species have been described by (Rojas et al., 2010) *C. tropicale* and *C. ignotum* and they are considered frequently asymptomatic in cocoa crops. In our study, we identified 14 species associated with the cocoa fruits: *Colletotrichum chlorophyti, Colletotrichum fioriniae, Colletotrichum gloeosporioides, Colletotrichum graminicola, Colletotrichum higginsianum, Colletotrichum incanum, Colletotrichum nymphaeae, Colletotrichum orbiculare, Colletotrichum orchidophilum, Colletotrichum salicis, Colletotrichum siamense, Colletotrichum simmondsii, Colletotrichum sublineola*, and *Colletotrichum tofieldiae*.

The second species with the highest number of transcripts assigned (440) was the filamentous fungus *Acremonium chrysogenum* (Ascomycota), a specie with industrial importance due to its ability to produce cephalosporin C (CPC), the main source for the production of different antibiotics in the industry (Hu and Zhu, 2016). The necrotrophic phytopathogen *Neonectria ditíssima* (Ascomycota) responsible for the European canker disease (EC) has 229 transcripts in our libraries, it is one of the most damaging apple diseases worldwide and has been recorded to be present in North and South American apple crops resulting in tree loss (Florez et al., 2020).

One of the 10 most abundant fungal genera in the microbiota was *Trichoderma*. Species such as *T. theobromicola* and *T. paucisporum* have been isolated from cocoa in South America, both inhibited the *in vitro* development of *M. roreri* (Samuels et al., 2006). *Trichoderma* species are common in soil and root ecosystems and are widely studied due to their great ability to produce metabolites with the potential to inhibit other microorganisms, parasitize other fungi, and compete with other microorganisms (Harman, 2006). Isolates of *Trichoderma* sp. species *T. virens and T. harzianum* have shown promise for the control of *M. roreri* (Reyes-Figueroa et al., 2016). Several isolates have been described as potential biological controls of moniliasis, such as *Trichoderma* isolates from different regions of Colombia, which have demonstrated mycelial growth antagonistic potential against strains of the fungus *M. roreri* (Suárez and Cabrales, 2008). A biocontrol strain using the fungus *T. reesei* (C2A) exhibited, *in vitro*, mycoparasitic activity, reducing 62% of the mycelial growth in *Fusarium oxysporum* (Gonzalez et al., 2020). These results reinforce the potential of strains of *Trichoderma* for the biological formulations to control the Moniliase disease (Leiva et al., 2020). *Trichoderma harzianum Rifai* from an infected cocoa pod is able to produce nonanoic (pelargonic) acid, which significantly reduces the spore germination of *M. roreri in vitro* (Aneja et al., 2005). The species *T. viride* can produce secondary metabolites (viridin and gliovirin) that have a synergistic effect for inhibiting the mycelial growth and conidium germination of fungal species *Phythopthora*, *P. palmivora*, and *P. megakaria* (Pakora et al., 2018). In our study, the following species were identified, *T. virens, T. reesei, T. parareesei, T. harzianum, T. guizhouense, T. gamsii*, and *T. atroviride*. According to the literature, some species found in our study have the potential for biological control (Ferreira and Musumeci, 2021; Chochocca et al., 2022), and others can test the effects they will produce on the fungus *M. roreri in vitro*. We also identified in the 48-h spore library, transcripts related to the species *Beauveria bassiana* (11 assigned transcripts). *Beauveria bassiana* is considered endophytic in nature (Amobonye et al., 2020). In addition to having a detrimental impact on insect survival, inoculation of *B. bassiana* into cowpea plants increased plant height, leaf count and dry mass (Pachoute et al., 2021).

In our study, we also evaluated viral diversity. There were three viral contigs assigned to the *Narnaviridae* by similarity search and phylogenetic analysis. All three contigs assembled in our study represented novel genomes. According to the ICTV, two species are described for this family: the *Saccharomyces 20S RNA narnavirus* and *Saccharomyces 23S RNA narnavirus*, which have genomic compositions of positive-sense single-stranded RNAs (ssRNA viruses (+); Lefkowitz et al., 2018). A characteristic of the members of this family is that they contain simpler genomes than any RNA virus, ranging from 2.3 to 3.6 kb coding only a single polypeptide that has an RNA-dependent RNA polymerase domain (Hillman and Cai, 2013). Members of this family have been reported as fungal viruses or mycoviruses, being found in a variety of fungal species associated with asymptomatic infections (Ghabrial and Suzuki, 2008). Additionally, they have been found in pathogenic fungi, such as *Rhizoctonia solani*, *Magnaporthe oryzae*, *Cercospora beticola* (Abdoulaye et al., 2019; Liu et al., 2020; Li et al., 2021) and the ectomycorrhizal fungus *Geopora sumneriana* (Sahin and Akata, 2019).

Metagenomic approaches can be useful for detecting and identifying new mycoviruses (Son et al., 2015). These methods are rapidly increasing viral identification and providing evidence of their high abundance and taxonomic complexity (García-Pedrajas et al., 2019). Mycovirus infections are usually cryptic (asymptomatic), but investigations focus on the potential hypovirulence they can cause in the host fungus, a process that can be researched in the context of providing sustainable biological control of fungal diseases (Abdoulaye et al., 2019). For example, the fungus *Colletotrichum* sp. is one of the most economically important phytopathogens and can be infected by many different species of viruses presenting modifications in its development (Casas et al., 2021).

The three contigs assembled in our study can be new viral genomes with similarities to the *Narnaviridae*. In our phylogenetic tree, Contig 1 was next to the species *Magnaporthe Oryzae Narnavirus 1*, and was described in the rice blast fungus *Magnaporthe oryzae* (Lin et al., 2020). Contig 2 grouped with the species *Oidiodendron maius splipalmivirus 1* (Sutela et al., 2020). Contig 3 formed a cluster with the species *Erysiphe necator-associated narnavirus 22* and *Monilinia narnavirus H*. Our results demonstrate that viral diversity is unexplored, and many viral species can be detected and identified infecting fungal species. These viruses may also present function as biological control agents since they can reduce fungal growth. However, although we have considerable amount of data that indicated *M. roreri* as host, since we were not able to perform further experiments, we still uncertain if the viruses are specifically infecting M. roreri, other fungal species or multiple species within that microenvironment assessed. Therefore, they were named with the prefix “*M. roreri*-associated” viruses.

Each plant can present a unique microbiome. The associated microorganisms might colonize different plant tissues and their abundance will depend on the nutrient availability, the planting immune-response system, the competence, the associations between other microorganisms, among others. In our study, we explored the microbiota associated with the infection caused by *M. roreri* in cocoa and found a wide variety of fungal, bacterial, and viral species. Moreover, we describe the presence of three new viral genomes infecting *M. roreri*. Our results can help other studies investigate the role of microorganisms during infection of *M. roreri* in cocoa fruits.

## Data availability statement

The datasets presented in this study can be found in online repositories. The names of the repository/repositories and accession number(s) can be found at: http://www.ncbi.nlm.nih.gov/sra, PRJNA854689.

## Author contributions

EA: conceptualization, methodology, and supervision. KN, BR, NH, and PF: formal analysis. CP, KG, and EA-G: resources. BR, PF, and EA: writing of the original draft. BR, PF, CP, KG, LC, and EA: reviewing and editing. CP: funding acquisition. All authors contributed to the article and approved the submitted version.

## Funding

This work was funded by the Coordenação de Aperfeiçoamento de Pessoal de Nível Superior (CAPES, Finance Code 001), Conselho Nacional de Desenvolvimento Científico e Tecnológico (CNPq #490655/2013-0, #303765/2019-4, and #403670/2020-9), Fundação de Apoio á Pesquisa do Estado da Bahia (Convênio 067/2013).

## Conflict of interest

The authors declare that the research was conducted in the absence of any commercial or financial relationships that could be construed as a potential conflict of interest.

## Publisher’s note

All claims expressed in this article are solely those of the authors and do not necessarily represent those of their affiliated organizations, or those of the publisher, the editors and the reviewers. Any product that may be evaluated in this article, or claim that may be made by its manufacturer, is not guaranteed or endorsed by the publisher.

## Footnotes

^1^ https://usegalaxy.eu/

^2^ https://kaiju.binf.ku.dk/server

^3^ http://tree.bio.ed.ac.uk/software/figtree/
