# Supplementary material for: Characterization of the microbiota dynamics associated with Moniliophthora roreri, causal agent of cocoa frosty pod rot disease, reveals new viral species
Source: Front Microbiol. 2023 Feb 3;13:1053562. doi: 10.3389/fmicb.2022.1053562 (PMC9936985; doi:10.3389/fmicb.2022.1053562)
Supplement: Supplementary file 1 [file Data_Sheet_1.pdf]

Library	Transcripts ≥5		
	**Genus**	**Species**	**Transcripts**
Spores_0hai	*Not specified*	*Not specified*	26
	Not specified	*Not specified*	16
	Not specified	*Not specified*	6
	Scleroderma	*Scleroderma citrinum*	5
	Schizophyllum	*Schizophyllum commune*	5
	Gymnopus	*Gymnopus luxurians*	5
	Rhizoctonia	*Rhizoctonia solani*	5
	**Transcripts <5**		
	**Genus**	**Species**	**Transcripts**
Spores_0hai	*Colletotrichum*	*Colletotrichum gloeosporioides*	4
	Not specified	*Not specified*	3
	Serpula	*Serpula lacrymans*	3
	Not specified	*Not specified*	3
	Fibulorhizoctonia	*Fibulorhizoctonia sp. CBS 109695*	3
	Hypsizygus	*Hypsizygus marmoreus*	3
	Ustilago	*Ustilago maydis*	2
	Plicaturopsis	*Plicaturopsis crispa*	2
	Laccaria	*Laccaria amethystina*	2
	Galerina	*Galerina marginata*	2
	Pleurotus	*Pleurotus ostreatus*	2
	Hebeloma	*Hebeloma cylindrosporum*	2
	Agaricus	*Agaricus bisporus*	2
	Not specified	*Not specified*	2
	Fibroporia	*Fibroporia radiculosa*	2
	Phlebiopsis	*Phlebiopsis gigantea*	2
	Exidia	*Exidia glandulosa*	2
	Not specified	*Not specified*	2
	Aspergillus	*Aspergillus lentulus*	2
	Amanita	*Amanita muscaria*	1

Library	Transcripts <5		
	**Genus**	**Species**	**Transcripts**
Spores_0hai	*Stereum*	*Stereum hirsutum*	1
	Dichomitus	*Dichomitus squalens*	1
	Phanerochaete	*Phanerochaete carnosa*	1
	Neolentinus	*Neolentinus lepideus*	1
	Gloeophyllum	*Gloeophyllum trabeum*	1
	Punctularia	*Punctularia strigosozonata*	1
	Histoplasma	*Histoplasma capsulatum*	1
	Talaromyces	*Talaromyces stipitatus*	1
	Talaromyces	*Talaromyces marneffei*	1
	Penicillium	*Not specified*	1
	Aspergillus	*Aspergillus niger*	1
	Aspergillus	*Aspergillus luchuensis*	1
	Aspergillus	*Not specified*	1
	Not specified	*Not specified*	1
	Choanephora	*Choanephora cucurbitarum*	1
	Mixia	*Mixia osmundae*	1
	Jaapia	*Jaapia argillacea*	1
	Suillus	*Suillus luteus*	1
	Not specified	*Not specified*	1
	Pisolithus	*Not specified*	1
	Hypoxylon	*Hypoxylon sp. EC38*	1
	Trichoderma	*Trichoderma virens*	1
	Baudoinia	*Baudoinia panamericana*	1
	Mycena	*Mycena chlorophos*	1
	Coprinopsis	*Coprinopsis cinerea*	1
	Cylindrobasidium	*Cylindrobasidium torrendii*	1
	Lentinula	*Lentinula edodes*	1
	Not specified	*Not specified*	1
	Leucoagaricus	*Leucoagaricus sp. SymC.cos*	1

Abundance of fungal species identified in the metatranscriptome of sample derived from Spores 0hs.

Library		Transcripts ≥5	
	Genus	Species	Transcripts
	Escherichia	*Escherichia coli*	54
	Not Specified	*Not Specified*	48
	Not Specified	*Not Specified*	26
	Not Specified	*Not Specified*	15
	Pantoea	*Pantoea dispersa*	15
	Xanthomonas	*Xanthomonas citri*	15
Spores_0hai	*Pantoea*	*Pantoea ananatis*	12
	Not Specified	*Not Specified*	10
	Klebsiella	*Klebsiella pneumoniae*	10
	Thiomonas	*Thiomonas sp. CB2*	8
	Pantoea	*Not Specified*	7
	Not Specified	*Not Specified*	7
	Curvibacter	*Curvibacter putative symbiont of Hydra magnipapillata*	7
	Not Specified	*Not Specified*	6

Library		Transcripts <5	
	Genus	Species	Transcripts
	Vibrio	*Not Specified*	4
	Klebsiella	*Not Specified*	4
	Ralstonia	*Ralstonia sp. NT80*	4
	Not Specified	*uncultured bacterium A1Q1_fos_4*	3
	Staphylococcus	*Staphylococcus hominis*	3
	Not Specified	*methanotrophic bacterial endosymbiont of Bathymodiolus sp.*	3
Spores_0hai	*Pseudomonas*	*Not Specified*	3
	Haemophilus	*Haemophilus influenzae*	3
	Izhakiella	*Not Specified*	3
	Brucella	*Not Specified*	3
	Xanthomonas	*Xanthomonas fuscans*	2
	Legionella	*Not Specified*	2
	Not Specified	*Plautia stali symbiont*	2

Library		Transcripts <5	
	Genus	Species	Transcripts
	Streptomyces	*Streptomyces sp. e14*	1
	Stenotrophomonas	*Stenotrophomonas sp. RIT309*	1
	Vibrio	*Vibrio parahaemolyticus*	1
	Vibrio	*Vibrio campbellii*	1
	Vibrio	*Vibrio cholerae*	1
	Pseudomonas	*Not Specified*	1
Spores_0hai	*Pseudomonas*	*Pseudomonas sp. DRA525*	1
	Pseudomonas	*Not Specified*	1
	Halomonas	*Halomonas titanicae*	1
	Tatlockia	*Not Specified*	1
	Pantoea	*Pantoea sp. At-9b*	1
	Pantoea	*Pantoea rwandensis*	1
	Pantoea	*Pantoea rodasii*	1
	Erwinia	*Erwinia sp. 9145*	1
	Yokenella	*Yokenella regensburgei*	1
	Shigella	*Shigella sonnei*	1
	Shigella	*Shigella flexneri*	1
	Salmonella	*Salmonella enterica*	1
	Pluralibacter	*Pluralibacter gergoviae*	1
Spores_0hai	*Kluyvera*	*Kluyvera cryocrescens*	1
	Izhakiella	*Izhakiella sp. D4N98*	1
	Escherichia	*Not Specified*	1
	Ralstonia	*Ralstonia sp. UNCCL144*	1
	Ralstonia	*Ralstonia solanacearum*	1
	Ralstonia	*Not Specified*	1
	Cupriavidus	*Cupriavidus sp. BIS7*	1
	Cupriavidus	*Cupriavidus pauculus*	1
	Cupriavidus	*Cupriavidus necator*	1
	Taylorella	*Taylorella asinigenitalis*	1

Spores_0h ai	*Not Specified*	*uncultured beta proteobacterium CBNPD1 BAC clone 578*	2
	Cupriavidus	*Cupriavidus metallidurans*	2
	Burkholderia	*Not Specified*	2
	Burkholderia	*Burkholderia sp. JS23*	2
	Burkholderia	*Not Specified*	2
	Cutibacterium	*Cutibacterium acnes*	2
	Mannheimia	*Mannheimia haemolytica*	2
	Hymenobacter	*Hymenobacter sp. CCM 8649*	1
	Not Specified	*uncultured bacterium*	1
	Clostridium	*Not Specified*	1
	Streptococcus	*Streptococcus pneumoniae*	1
	Paenibacillus	*Paenibacillus sp. FF9*	1
	Pseudoflavonifractor	*Pseudoflavonifractor capillosus*	1
Spores_0h ai	*Enterobacter*	*Enterobacter sp. MGH 16*	1
	Enterobacter	*Not Specified*	1
	Cronobacter	*Cronobacter muytjensii*	1
	Thiobacillus	*Not Specified*	1
	Sutterella	*Sutterella wadsworthensis CAG:135*	1
	Not Specified	*uncultured alpha proteobacterium HF0130_06E21*	1
	Roseibium	*Roseibium sp. TrichSKD4*	1
	Rhizobium	*Rhizobium oryzae*	1
	Agrobacterium	*Agrobacterium genomosp. 2*	1
	Methylobacterium	*Methylobacterium mesophilicum*	1
	Brucella	*Brucella suis*	1
	Aurantimonas	*Aurantimonas manganoxydans*	1
	Not Specified	*uncultured Acidobacteria bacterium HF4000_26D02*	1

Abundance of bacteria species identified in the metatranscriptome of sample derived from Spores 0hs.

Library		Transcripts ≥5	
	**Genus**	**Species**	**Transcripts**
	Penicillium	*Not specified*	42
	Penicillium	*Penicillium subrubescens*	41
	Not specified	*Not specified*	32
	Not specified	*Not specified*	30
	Coniochaeta	*Coniochaeta ligniaria*	24
	Not specified	*Not specified*	17
	Not specified	*Not specified*	17
	Penicillium	*Penicillium brasilianum*	16
	Not specified	*Not specified*	12
Spores_8hai	*Not specified*	*Not specified*	11
	Yamadazyma	*[Candida] tenuis*	9
	Not specified	*Not specified*	8
	Not specified	*Not specified*	8
	Serpula	*Serpula lacrymans*	8
	Penicillium	*Penicillium oxalicum*	7
	Rachicladosporium	*Not specified*	7
	Saitoella	*Saitoella complicata*	7
	Not specified	*Not specified*	5
	Puccinia	*Puccinia striiformis*	5
	Schizophyllum	*Schizophyllum commune*	5
	Chaetomium	*Chaetomium globosum*	5
	Aspergillus	*Not specified*	5
	Rhizoctonia	*Rhizoctonia solani*	5
	Colletotrichum	*Colletotrichum gloeosporioides*	5

Library		Transcripts <5	
	**Genus**	**Species**	**Transcripts**
	Neurospora	*Not specified*	4
	Phaeoacremonium	*Phaeoacremonium minimum*	4
	Aspergillus	*Aspergillus brasiliensis*	4
	Choanephora	*Choanephora cucurbitarum*	4
	Fibulorhizoctonia	*Fibulorhizoctonia sp. CBS 109695*	3
	Gymnopus	*Gymnopus luxurians*	3
	Not specified	*Not specified*	3
	Candida	*Candida tropicalis*	3

Library		Transcripts <5	
	**Genus**	**Species**	**Transcripts**
	Lentinula	*Lentinula edodes*	1
	Not specified	*Not specified*	1
	Hebeloma	*Hebeloma cylindrosporum*	1
	Sistotremastrum	*Sistotremastrum niveocremeum*	1
	Pycnoporus	*Pycnoporus coccineus*	1
	Fibroporia	*Fibroporia radiculosa*	1
	Dichomitus	*Dichomitus squalens*	1
	Phlebiopsis	*Phlebiopsis gigantea*	1
	Trametes	*Not specified*	1
	Neolentinus	*Neolentinus lepideus*	1
	Exidia	*Exidia glandulosa*	1
	Not specified	*Not specified*	1
	Naumovozyma	*Naumovozyma dairenensis*	1
	Naumovozyma	*Naumovozyma castellii*	1
	Not specified	*Not specified*	1
	Ogataea	*Ogataea polymorpha*	1
Spores_8hai	*Metschnikowia*	*Metschnikowia bicuspidata*	1
	Clavispora	*[Candida] duobushaemulonii*	1
	Clavispora	*Clavispora lusitaniae*	1
	Spathaspora	*Spathaspora passalidarum*	1
	Meyerozyma	*Meyerozyma guilliermondii*	1
	Hyphopichia	*Hyphopichia burtonii*	1
	Debaryomyces	*Debaryomyces hansenii*	1
	Debaryomyces	*Debaryomyces fabryi*	1
	Ascoidea	*Ascoidea rubescens*	1
	Hypoxylon	*Hypoxylon sp. CO27-5*	1
	Hypoxylon	*Hypoxylon sp. CI-4A*	1
	Pestalotiopsis	*Pestalotiopsis fici*	1
	Sordaria	*Sordaria macrospora*	1
	Neurospora	*Neurospora crassa*	1
	Podospora	*Podospora anserina*	1
	Sporothrix	*Sporothrix insectorum*	1
	Grosmannia	*Grosmannia clavigera*	1
	Magnaporthe	*Magnaporthe oryzae*	1

	Eutypa	*Eutypa lata*	3
Spores_8hai	*Verticillium*	*Not specified*	3
	Colletotrichum	*Not specified*	3
	Sphaerulina	*Sphaerulina musiva*	3
	Aspergillus	*Aspergillus aculeatus*	3
	Aureobasidium	*Aureobasidium pullulans*	3
	Bipolaris	*Bipolaris maydis*	2
	Ustilago	*Ustilago maydis*	2
	Pleurotus	*Pleurotus ostreatus*	2
	Hypsizygus	*Hypsizygus marmoreus*	2
	Not specified	*Not specified*	2
	Gloeophyllum	*Gloeophyllum trabeum*	2
	Wickerhamomyces	*Not specified*	2
	Not specified	*Not specified*	2
	Not specified	*Not specified*	2
	Not specified	*Not specified*	2
	Thermothelomyces	*Thermothelomyces thermophila*	2
	Ophiostoma	*Ophiostoma piceae*	2
	Not specified	*Not specified*	2
	Fusarium	*Not specified*	2
	Botrytis	*Botrytis cinerea*	2
	Not specified	*Not specified*	2
	Rachicladosporium	*Rachicladosporium antarcticum*	2
	Ascosphaera	*Ascosphaera flava*	2
	Talaromyces	*Talaromyces marneffei*	2
Spores_8hai	*Penicillium*	*Penicillium decumbens*	2
	Penicilliopsis	*Penicilliopsis zonata*	2
	Aspergillus	*Aspergillus lentulus*	2
	Aspergillus	*Aspergillus flavus*	2
	Aspergillus	*Aspergillus clavatus*	2
	Not specified	*Not specified*	2
	Rhinocladiella	*Rhinocladiella mackenziei*	2
	Capronia	*Capronia epimyces*	2
	Not specified	*Not specified*	2
	Parastagonospora	*Parastagonospora nodorum*	1
	Epicoccum	*Epicoccum nigrum*	1

	Valsa	*Valsa mali*	1
	Diaporthe	*Not specified*	1
	Not specified	*Not specified*	1
	Stachybotrys	*Stachybotrys chlorohalonata*	1
	Tolypocladium	*Tolypocladium ophioglossoides*	1
	Tolypocladium	*Tolypocladium inflatum*	1
Spores_8hai	*Ophiocordyceps*	*Ophiocordyceps unilateralis*	1
	Ophiocordyceps	*Ophiocordyceps nutans*	1
	Hirsutella	*Hirsutella minnesotensis*	1
	Not specified	*Not specified*	1
	Fusarium	*Fusarium langsethiae*	1
	Ustilaginoidea	*Ustilaginoidea virens*	1
	Trichoderma	*Trichoderma virens*	1
	Trichoderma	*Not specified*	1
	Moelleriella	*Moelleriella libera*	1
	Metarhizium	*Metarhizium majus*	1
	Not specified	*Not specified*	1
	Colletotrichum	*Colletotrichum salicis*	1
	Colletotrichum	*Colletotrichum orbiculare*	1
	Pseudogymnoascus	*Pseudogymnoascus sp. VKM F-4246*	1
	Pseudogymnoascus	*Pseudogymnoascus sp. VKM F-3775*	1
	Pseudogymnoascus	*Not specified*	1
	Sclerotinia	*Sclerotinia sclerotiorum*	1
	Not specified	*Not specified*	1
	Phialocephala	*Phialocephala scopiformis*	1
	Glarea	*Glarea lozoyensis*	1
	Clohesyomyces	*Clohesyomyces aquaticus*	1
	Not specified	*Not specified*	1
	Hortaea	*Hortaea werneckii*	1
	Aureobasidium	*Not specified*	1
Spores_8hai	*Zymoseptoria*	*Zymoseptoria tritici*	1
	Zymoseptoria	*Zymoseptoria brevis*	1
	Pseudocercospora	*Pseudocercospora fijiensis*	1
	Mycosphaerella	*Mycosphaerella eumusae*	1
	Dothistroma	*Dothistroma septosporum*	1
	Cladosporium	*Cladosporium herbarum*	1

	Not specified	*fungal sp. No.14919*	1
	Not specified	*fungal sp. No.11243*	1
	Basidiobolus	*Basidiobolus meristosporus*	1
	Anaeromyces	*Anaeromyces robustus*	1
	Mitosporidium	*Mitosporidium daphniae*	1
	Melanopsichium	*Melanopsichium pennsylvanicum*	1
	Malassezia	*Malassezia globosa*	1
	Mixia	*Mixia osmundae*	1
	Leucosporidium	*Leucosporidium creatinivorum*	1
	Cryptococcus	*Cryptococcus amylolentus*	1
	Cryptococcus	*Not specified*	1
	Jaapia	*Jaapia argillacea*	1
	Rhizopogon	*Rhizopogon vinicolor*	1
Spores_8hai	*Not specified*	*Not specified*	1
	Scleroderma	*Scleroderma citrinum*	1
	Pisolithus	*Pisolithus microcarpus*	1
	Pisolithus	*Not specified*	1
	Plicaturopsis	*Plicaturopsis crispa*	1
	Galerina	*Galerina marginata*	1
	Volvariella	*Volvariella volvacea*	1
	Cylindrobasidium	*Cylindrobasidium torrendii*	1
	Rhizoclosmatium	*Rhizoclosmatium globosum*	1

	Not specified	*Not specified*	1
	Coniosporium	*Coniosporium apollinis*	1
	Acidomyces	*Acidomyces richmondensis*	1
	Not specified	*Not specified*	1
	Umbilicaria	*Umbilicaria pustulata*	1
	Lopezaria	*Lopezaria versicolor*	1
	Histoplasma	*Histoplasma capsulatum*	1
	Emmonsia	*Emmonsia crescens*	1
	Talaromyces	*Talaromyces purpureogenus*	1
	Byssochlamys	*Byssochlamys spectabilis*	1
	Penicillium	*Penicillium steckii*	1
	Penicillium	*Penicillium nordicum*	1
	Penicillium	*Penicillium italicum*	1
	Penicillium	*Penicillium antarcticum*	1
	Aspergillus	*Aspergillus udagawae*	1
	Aspergillus	*Aspergillus terreus*	1
	Aspergillus	*Aspergillus ruber*	1
	Endocarpon	*Endocarpon pusillum*	1
Spores_8hai	*Exophiala*	*Exophiala mesophila*	1
	Arthrobotrys	*Arthrobotrys oligospora*	1
	Gonapodya	*Gonapodya prolifera*	1

Abundance of fungal species identified in the metatranscriptome of sample derived from Spores 8hs.

Library		Transcripts ≥5		Library		Transcripts <5	
	Genus	Species	Transcripts		Genus	Species	Transcripts
	Pseudomonas	*Not Specified*	2687		*Carnobacterium*	*Carnobacterium maltaromaticum*	1
	Pantoea	*Pantoea dispersa*	1290		*Not Specified*	*Not Specified*	1
	Not Specified	*Not Specified*	1268		*Staphylococcus*	*Staphylococcus hominis*	1
	Pseudomonas	*Pseudomonas sp. Bc-h*	615		*Paenisporosarcina*	*Paenisporosarcina sp. HGH0030*	1
	Pseudomonas	*Pseudomonas abietaniphila*	432		*Paenibacillus*	*Paenibacillus sp. FF9*	1
	Pseudomonas	*Pseudomonas sp. NFR16*	405		*Gemella*	*Gemella morbillorum*	1
	Pseudomonas	*Pseudomonas putida*	314		*Not Specified*	*Not Specified*	1
	Not Specified	*Not Specified*	176		*Microcystis*	*Microcystis aeruginosa*	1
Spores_8hai	*Escherichia*	*Escherichia coli*	157	Spores_8hai	*Nocardiopsis*	*Nocardiopsis trehalosi*	1
	Pseudomonas	*Pseudomonas graminis*	141		*Streptomyces*	*Streptomyces sp. C*	1
	Not Specified	*Not Specified*	128		*Streptomyces*	*Streptomyces pristinaespiralis*	1
	Pseudomonas	*Pseudomonas syringae*	120		*Streptomyces*	*Streptomyces himastatinicus*	1
	Not Specified	*Not Specified*	110		*Streptomyces*	*Streptomyces ghanaensis*	1
	Pantoea	*Not Specified*	108		*Streptomyces*	*Not Specified*	1
	Not Specified	*Not Specified*	85		*Propionibacterium*	*Propionibacterium sp. HMSC067A02*	1
	Pseudomonas	*Pseudomonas lutea*	81		*Not Specified*	*Not Specified*	1
	Pantoea	*Pantoea ananatis*	56		*Rothia*	*Rothia mucilaginosa*	1
	Pseudomonas	*Pseudomonas sp. NFACC02*	53		*Tetrasphaera*	*Tetrasphaera japonica*	1
	Pseudomonas	*Pseudomonas fluorescens*	52		*Rhodococcus*	*Rhodococcus sp. RD6.2*	1
	Pseudomonas	*Not Specified*	39		*Corynebacterium*	*Corynebacterium diphtheriae*	1
	Pseudomonas	*Pseudomonas sp. StFLB209*	32		*Catenulispora*	*Catenulispora acidiphila*	1
	Not Specified	*Not Specified*	28		*Bifidobacterium*	*Bifidobacterium longum*	1
	Pseudomonas	*Pseudomonas cichorii*	26		*Actinomyces*	*Actinomyces nasicola*	1
	Not Specified	*Type-C symbiont of Plautia stali*	24		*Not Specified*	*Not Specified*	1
	Not Specified	*Plautia stali symbiont*	22		*Desulfovibrio*	*Desulfovibrio indicus*	1
	Pseudomonas	*Pseudomonas fuscovaginae*	20		*Not Specified*	*Gammaproteobacteria bacterium RIFCSPLOWO2_02_FULL_57_10*	1
Spores_8hai	*Salmonella*	*Salmonella enterica*	18	Spores_8hai	*Not Specified*	*Gammaproteobacteria bacterium MFB021*	1
	Pseudomonas	*Pseudomonas chlororaphis*	17		*Not Specified*	*Bathymodiolus azoricus thioautotrophic gill symbiont*	1
	Pseudomonas	*Pseudomonas sp. Leaf127*	17		*Gallaecimonas*	*Gallaecimonas pentaromativorans*	1
	Shigella	*Shigella sonnei*	17		*Xanthomonas*	*Xanthomonas translucens*	1
	Cutibacterium	*Cutibacterium acnes*	17		*Xanthomonas*	*Xanthomonas sp. Mitacek01*	1
	Ralstonia	*Ralstonia sp. NT80*	14		*Rhodanobacter*	*Not Specified*	1
	Streptococcus	*Streptococcus pneumoniae*	14		*Luteibacter*	*Luteibacter sp. 329MFSha*	1
	Pseudomonas	*Pseudomonas xanthomarina*	12		*Vibrio*	*Not Specified*	1
	Pseudomonas	*Pseudomonas sp. M47T1*	12		*Aliivibrio*	*Aliivibrio fischeri*	1

Spores_8hai	*Not Specified*	*gamma proteobacterium L18*	11
	Pseudomonas	*Pseudomonas monteilii*	10
	Pseudomonas	*Pseudomonas frederiksbergensis*	10
	Xanthomonas	*Xanthomonas citri*	10
	Enterobacter	*Enterobacter cloacae*	10
	Pseudomonas	*Pseudomonas vranovensis*	9
	Pseudomonas	*Pseudomonas sp. WCHP16*	9
	Pseudomonas	*Pseudomonas parafulva*	9
	Pseudomonas	*Pseudomonas helleri*	9
	Not Specified	*uncultured bacterium*	9
	Pseudomonas	*Pseudomonas entomophila*	9
	Pseudomonas	*Pseudomonas aeruginosa*	9
	Pseudomonas	*Pseudomonas plecoglossicida*	8
	Pseudomonas	*Pseudomonas agarici*	8
	Haemophilus	*Haemophilus influenzae*	8
	Burkholderia	*Not Specified*	8
	Ralstonia	*Not Specified*	7
	Pseudomonas	*Pseudomonas vancouverensis*	7
	Pseudomonas	*Pseudomonas cremoricolorata*	7
Spores_8hai	*Curvibacter*	*Curvibacter putative symbiont of Hydra magnipapillata*	7
	Staphylococcus	*Staphylococcus aureus*	7
	Pseudomonas	*Pseudomonas mandelii*	7
	Pseudomonas	*Pseudomonas syringae group genomosp. 3*	7
	Vibrio	*Vibrio alginolyticus*	6
	Pseudomonas	*Pseudomonas sp. PH1b*	6
	Pseudomonas	*Pseudomonas sp. GEV388*	6
	Pseudomonas	*Not Specified*	6
	Pseudomonas	*Pseudomonas japonica*	6
	Pseudomonas	*Pseudomonas extremaustralis*	6
	Pantoea	*Pantoea vagans*	6
	Ralstonia	*Ralstonia pickettii*	6
	Pantoea	*Pantoea rwandensis*	5
	Not Specified	*Not Specified*	5
	Streptococcus	*Streptococcus pyogenes*	5
	Mycobacterium	*Mycobacterium abscessus*	5
	Pseudomonas	*Pseudomonas viridiflava*	5
	Pseudomonas	*Pseudomonas syringae group genomosp. 2*	5
Spores_8hai	*Not Specified*	*Pseudomonadales bacterium RIFCSPLOWO2_12_59_9*	1
	Not Specified	*Pseudomonadales bacterium RIFCSPHIGHO2_02_FULL_60_43*	1
	Pseudomonas	*Pseudomonas thermotolerans*	1
	Pseudomonas	*Pseudomonas syringae group genomosp. 7*	1
	Pseudomonas	*Pseudomonas savastanoi*	1
	Pseudomonas	*Pseudomonas ficuserectae*	1
	Pseudomonas	*Pseudomonas coronafaciens*	1
	Pseudomonas	*Pseudomonas caricapapayae*	1
	Pseudomonas	*Pseudomonas luteola*	1
	Pseudomonas	*Not Specified*	1
	Pseudomonas	*Pseudomonas sp. Z003-0.4C(8344-21)*	1
	Pseudomonas	*Pseudomonas sp. W15Feb9B*	1
	Pseudomonas	*Pseudomonas sp. UW4*	1
	Pseudomonas	*Pseudomonas sp. URMO17WK12:I5*	1
	Pseudomonas	*Pseudomonas sp. URMO17WK12:I12*	1
	Pseudomonas	*Pseudomonas sp. URMO17WK12:I11*	1
	Pseudomonas	*Pseudomonas sp. URIL14HWK12:I5*	1
	Pseudomonas	*Pseudomonas sp. UC 17F4*	1
	Pseudomonas	*Pseudomonas sp. TTU2014-080ASC*	1
Spores_8hai	*Pseudomonas*	*Pseudomonas sp. T*	1
	Pseudomonas	*Pseudomonas sp. S3E12*	1
	Pseudomonas	*Pseudomonas sp. Root329*	1
	Pseudomonas	*Pseudomonas sp. R81*	1
	Pseudomonas	*Pseudomonas sp. R45(2017)*	1
	Pseudomonas	*Pseudomonas sp. P818*	1
	Pseudomonas	*Pseudomonas sp. NFIX28*	1
	Pseudomonas	*Pseudomonas sp. NFACC45*	1
	Pseudomonas	*Pseudomonas sp. NFACC25*	1
	Pseudomonas	*Pseudomonas sp. NBRC 111133*	1
	Pseudomonas	*Pseudomonas sp. NBRC 111124*	1
	Pseudomonas	*Pseudomonas sp. NBRC 111119*	1
	Pseudomonas	*Pseudomonas sp. MRSN12121*	1
	Pseudomonas	*Pseudomonas sp. MOIL14HWK12:I2*	1
	Pseudomonas	*Pseudomonas sp. ML96*	1
	Pseudomonas	*Pseudomonas sp. MIACH*	1
	Pseudomonas	*Pseudomonas sp. MF4836*	1
	Pseudomonas	*Pseudomonas sp. Leaf48*	1

	Pseudomonas	*Pseudomonas asturiensis*	5
	Pseudomonas	*Pseudomonas sp. S9*	5
	Pseudomonas	*Pseudomonas sp. Leaf58*	5
	Pseudomonas	*Pseudomonas gingeri*	5
	Pseudomonas	*Not Specified*	5
	Pseudomonas	*Pseudomonas batumici*	5
	Yersinia	*Yersinia pestis*	5
	Not Specified	*uncultured beta proteobacterium CBNPD1 BAC clone 578*	5
	Magnetospirillum	*Magnetospirillum gryphiswaldense*	5
	Klebsiella	*Klebsiella pneumoniae*	5

**Library**		**Transcripts <5**	
	**Genus**	**Species**	**Transcripts**
	Mycobacterium	*Mycobacterium tuberculosis*	4
	Lactobacillus	*Lactobacillus rhamnosus*	4
	Paenibacillus	*Paenibacillus odorifer*	4
	Propionibacterium	*Propionibacterium freudenreichii*	4
	Pseudomonas	*Pseudomonas stutzeri*	4
	Pseudomonas	*Pseudomonas sp. KK4*	4
	Pseudomonas	*Pseudomonas sp. ES3-33*	4
	Pseudomonas	*Pseudomonas sp. CB1*	4
Spores_8hai	*Pseudomonas*	*Pseudomonas guangdongensis*	4
	Pseudomonas	*Pseudomonas trivialis*	4
	Pseudomonas	*Pseudomonas endophytica*	4
	Edwardsiella	*Edwardsiella piscicida*	4
	Pararhodospirillum	*Pararhodospirillum photometricum*	4
	Brucella	*Not Specified*	4
	Stenotrophomonas	*Stenotrophomonas maltophilia*	4
	Acinetobacter	*Acinetobacter baumannii*	4
	Streptococcus	*Streptococcus salivarius*	3
	Streptococcus	*Streptococcus anginosus*	3
	Streptococcus	*Not Specified*	3
	Bacillus	*Bacillus pumilus*	3
	Actinomyces	*Actinomyces turicensis*	3
	Pseudomonas	*Pseudomonas sp. URHB0015*	3
	Pseudomonas	*Pseudomonas sp. SHC52*	3
	Pseudomonas	*Pseudomonas sp. Root562*	3
	Pseudomonas	*Pseudomonas sp. RIT-PI-q*	3
	Pseudomonas	*Pseudomonas sp. NBRC 111130*	3
Spores_8hai	*Pseudomonas*	*Pseudomonas sp. KG01*	3
	Pseudomonas	*Pseudomonas sp. CF161*	3
	Pseudomonas	*Pseudomonas sp. CBZ-4*	3

	Pseudomonas	*Pseudomonas sp. Leaf129*	1
	Pseudomonas	*Pseudomonas sp. LAB-08*	1
	Pseudomonas	*Pseudomonas sp. In5*	1
	Pseudomonas	*Pseudomonas sp. GM80*	1
	Pseudomonas	*Pseudomonas sp. GM78*	1
	Pseudomonas	*Pseudomonas sp. GM74*	1
	Pseudomonas	*Pseudomonas sp. GM50*	1
	Pseudomonas	*Pseudomonas sp. GM41(2012)*	1
	Pseudomonas	*Pseudomonas sp. GM33*	1
	Pseudomonas	*Pseudomonas sp. GM21*	1
	Pseudomonas	*Pseudomonas sp. GM18*	1
	Pseudomonas	*Pseudomonas sp. GM17*	1
	Pseudomonas	*Pseudomonas sp. FSL W5-0299*	1
	Pseudomonas	*Pseudomonas sp. CHM02*	1
	Pseudomonas	*Pseudomonas sp. CCOS 191*	1
	Pseudomonas	*Pseudomonas sp. C5pp*	1
	Pseudomonas	*Pseudomonas sp. B10*	1
	Pseudomonas	*Pseudomonas sp. Ant30-3*	1
	Pseudomonas	*Pseudomonas sp. ABAC61*	1
	Pseudomonas	*Pseudomonas sp. A3(2016)*	1
Spores_8hai	*Pseudomonas*	*Pseudomonas sp. 655*	1
	Pseudomonas	*Pseudomonas sp. 63_8*	1
	Pseudomonas	*Pseudomonas sp. 58 R 3*	1
	Pseudomonas	*Pseudomonas sp. 25 R 14*	1
	Pseudomonas	*Pseudomonas sp. 24 R 17*	1
	Pseudomonas	*Pseudomonas saponiphila*	1
	Pseudomonas	*Pseudomonas oryzihabitans*	1
	Pseudomonas	*Pseudomonas fulva*	1
	Pseudomonas	*Pseudomonas punonensis*	1
	Pseudomonas	*Pseudomonas psychrotolerans*	1
	Pseudomonas	*Pseudomonas denitrificans*	1
	Pseudomonas	*Pseudomonas pachastrellae*	1
	Pseudomonas	*Pseudomonas moorei*	1
	Pseudomonas	*Pseudomonas marincola*	1
	Pseudomonas	*Pseudomonas indica*	1
	Pseudomonas	*Pseudomonas veronii*	1
	Pseudomonas	*Pseudomonas synxantha*	1
	Pseudomonas	*Pseudomonas marginalis*	1
Spores_8hai	*Pseudomonas*	*Pseudomonas gessardii*	1
	Pseudomonas	*Pseudomonas deceptionensis*	1
	Pseudomonas	*Pseudomonas composti*	1

	Pseudomonas	*Pseudomonas sp. 5*	3
	Pseudomonas	*Pseudomonas sp. 11/12A*	3
	Pseudomonas	*Pseudomonas rhizosphaerae*	3
	Pseudomonas	*Pseudomonas reinekei*	3
	Pseudomonas	*Pseudomonas mosselii*	3
	Pseudomonas	*Pseudomonas mediterranea*	3
	Pseudomonas	*Pseudomonas taetrolens*	3
	Pseudomonas	*Pseudomonas fragi*	3
	Pseudomonas	*Pseudomonas alcaligenes*	3
	Not Specified	*Not Specified*	3
	Pantoea	*Pantoea sp. At-9b*	3
	Pantoea	*Pantoea sp. AS1*	3
	Candidatus Hamiltonella	*Candidatus Hamiltonella defensa*	3
	Klebsiella	*Not Specified*	3
	Escherichia	*Escherichia vulneris*	3
Spores_8hai	*Escherichia*	*Not Specified*	3
	Citrobacter	*Citrobacter sp. MGH109*	3
	Burkholderia	*Burkholderia mallei*	3
	Burkholderia	*Not Specified*	3
	Not Specified	*Not Specified*	3
	Roseibium	*Roseibium sp. TrichSKD4*	3
	Vibrio	*Vibrio parahaemolyticus*	3
	Pantoea	*Pantoea agglomerans*	3
	Not Specified	*uncultured bacterium A1Q1_fos_4*	2
	Not Specified	*uncultured bacterium 5H7*	2
	Not Specified	*Not Specified*	2
	Streptococcus	*Streptococcus equi*	2
	Streptococcus	*Streptococcus dysgalactiae*	2
	Enterococcus	*Enterococcus faecalis*	2
	Not Specified	*Not Specified*	2
	Collinsella	*Collinsella aerofaciens*	2
	Rothia	*Rothia dentocariosa*	2
	Rothia	*Not Specified*	2
	Paenarthrobacter	*Paenarthrobacter nicotinovorans*	2
	Gardnerella	*Gardnerella vaginalis*	2
	Actinomyces	*Actinomyces odontolyticus*	2
	Not Specified	*methanotrophic bacterial endosymbiont of Bathymodiolus sp.*	2
	Xanthomonas	*Xanthomonas fuscans*	2
	Vibrio	*Not Specified*	2
	Pseudomonas	*Pseudomonas versuta*	2
Spores_8hai	*Pseudomonas*	*Pseudomonas sp. RIT-PI-r*	2

	Pseudomonas	*Pseudomonas coleopterorum*	1
	Pseudomonas	*Pseudomonas lundensis*	1
	Pseudomonas	*Pseudomonas arsenicoxydans*	1
	Pseudomonas	*Pseudomonas antarctica*	1
	Pseudomonas	*Pseudomonas alcaliphila*	1
	Pseudomonas	*Pseudomonas mendocina*	1
	Pseudomonas	*Pseudomonas flavescens*	1
	Pseudomonas	*Pseudomonas citronellolis*	1
	Pseudomonas	*Pseudomonas caeni*	1
	Acinetobacter	*Acinetobacter towneri*	1
	Halomonas	*Halomonas titanicae*	1
	Chromohalobacter	*Chromohalobacter japonicus*	1
	Tatlockia	*Not Specified*	1
	Legionella	*Legionella londiniensis*	1
	Legionella	*Legionella lansingensis*	1
Spores_8hai	*Yersinia*	*Yersinia enterocolitica*	1
	Serratia	*Serratia liquefaciens*	1
	Serratia	*Not Specified*	1
	Providencia	*Providencia stuartii*	1
	Morganella	*Morganella morganii*	1
	Edwardsiella	*Not Specified*	1
	Tatumella	*Tatumella saanichensis*	1
	Pantoea	*Pantoea wallisii*	1
	Pantoea	*Pantoea sp. PSNIH1*	1
	Pantoea	*Pantoea sp. GM01*	1
	Pantoea	*Pantoea rodasii*	1
	Pantoea	*Pantoea cypripedii*	1
	Erwinia	*Erwinia sp. Leaf53*	1
	Erwinia	*Erwinia persicina*	1
	Erwinia	*Not Specified*	1
	Not Specified	*Type-E symbiont of Plautia stali*	1
	Not Specified	*Type-D symbiont of Plautia stali*	1
	Not Specified	*Enterobacteriaceae bacterium B14*	1
	Shigella	*Not Specified*	1
	Raoultella	*Raoultella planticola*	1
	Pluralibacter	*Pluralibacter gergoviae*	1
	Klebsiella	*Klebsiella sp. LTGPAF-6F*	1
	Klebsiella	*Klebsiella aerogenes*	1
	Izhakiella	*Not Specified*	1
	Enterobacter	*Enterobacter sp. FY-07*	1
Spores_8hai	*Enterobacter*	*Enterobacter ludwigii*	1

	Pseudomonas	*Pseudomonas sp. Pf153*	2		*Enterobacter*	*Enterobacter sp. MGH 16*	1
	Pseudomonas	*Pseudomonas sp. PA27(2017)*	2		*Enterobacter*	*Not Specified*	1
	Pseudomonas	*Pseudomonas sp. P482*	2		*Enterobacter*	*Not Specified*	1
	Pseudomonas	*Pseudomonas sp. P1.31*	2		*Cronobacter*	*Cronobacter universalis*	1
	Pseudomonas	*Pseudomonas sp. NBRC 111131*	2		*Cronobacter*	*Cronobacter turicensis*	1
	Pseudomonas	*Pseudomonas sp. MF6396*	2		*Cronobacter*	*Cronobacter malonaticus*	1
	Pseudomonas	*Pseudomonas sp. Leaf434*	2		*Citrobacter*	*Citrobacter freundii*	1
	Pseudomonas	*Pseudomonas sp. GM84*	2		*Cedecea*	*Cedecea davisae*	1
	Pseudomonas	*Pseudomonas sp. GM25*	2		*Pseudoalteromonas*	*Pseudoalteromonas sp. TB64*	1
	Pseudomonas	*Pseudomonas sp. CFII64*	2		*Marinobacter*	*Marinobacter sp. T13-3*	1
	Pseudomonas	*Pseudomonas sp. BAY1663*	2		*Marinobacter*	*Marinobacter hydrocarbonoclasticus*	1
	Pseudomonas	*Pseudomonas sp. B28(2017)*	2		*Aeromonas*	*Not Specified*	1
	Pseudomonas	*Pseudomonas sp. ATCC PTA-122608*	2		*Not Specified*	*Not Specified*	1
	Pseudomonas	*Pseudomonas sp. 22 E 5*	2		*Thauera*	*Not Specified*	1
	Pseudomonas	*Pseudomonas sp. 10-1B*	2		*Propionivibrio*	*Propionivibrio dicarboxylicus*	1
	Pseudomonas	*Pseudomonas prosekii*	2		*Dechloromonas*	*Dechloromonas aromatica*	1
	Pseudomonas	*Pseudomonas panipatensis*	2		*Not Specified*	*uncultured beta proteobacterium HF0010_04H24*	1
	Pseudomonas	*Pseudomonas otitidis*	2		*Nitrosomonas*	*Nitrosomonas mobilis*	1
	Pseudomonas	*Pseudomonas kuykendallii*	2		*Neisseria*	*Not Specified*	1
	Pseudomonas	*Pseudomonas knackmussii*	2		*Chromobacterium*	*Chromobacterium piscinae*	1
	Pseudomonas	*Pseudomonas guariconensis*	2		*Rubrivivax*	*Rubrivivax gelatinosus*	1
	Pseudomonas	*Pseudomonas grimontii*	2		*Rhizobacter*	*Rhizobacter gummiphilus*	1
	Pseudomonas	*Pseudomonas tolaasii*	2		*Massilia*	*Massilia alkalitolerans*	1
	Pseudomonas	*Pseudomonas rhodesiae*	2		*Massilia*	*Not Specified*	1
Spores_8hai	*Pseudomonas*	*Pseudomonas mucidolens*	2	Spores_8hai	*Herbaspirillum*	*Not Specified*	1
	Pseudomonas	*Pseudomonas costantinii*	2		*Duganella*	*Duganella sp. Root1480D1*	1
	Pseudomonas	*Pseudomonas corrugata*	2		*Variovorax*	*Variovorax sp. YR216*	1
	Pseudomonas	*Pseudomonas congelans*	2		*Variovorax*	*Not Specified*	1
	Pseudomonas	*Pseudomonas brenneri*	2		*Ramlibacter*	*Ramlibacter sp. Leaf400*	1
	Pseudomonas	*Pseudomonas delhiensis*	2		*Not Specified*	*Burkholderiaceae bacterium 16*	1
	Pseudomonas	*Pseudomonas brassicacearum*	2		*Ralstonia*	*Ralstonia sp. NFACC01*	1
	Pseudomonas	*Pseudomonas azotifigens*	2		*Ralstonia*	*Ralstonia mannitolilytica*	1
	Pseudomonas	*Pseudomonas alkylphenolica*	2		*Paraburkholderia*	*Paraburkholderia sartisoli*	1
	Pseudomonas	*Pseudomonas resinovorans*	2		*Paraburkholderia*	*Paraburkholderia sacchari*	1
	Pseudomonas	*Pseudomonas pseudoalcaligenes*	2		*Paraburkholderia*	*Paraburkholderia ferrariae*	1
	Pseudomonas	*Pseudomonas oleovorans*	2		*Paraburkholderia*	*Paraburkholderia aspalathi*	1
	Pseudomonas	*Pseudomonas nitroreducens*	2		*Paraburkholderia*	*Candidatus Paraburkholderia schumannianae*	1
	Pseudomonas	*Not Specified*	2		*Cupriavidus*	*Not Specified*	1
	Mannheimia	*Mannheimia haemolytica*	2		*Burkholderia*	*Burkholderia thailandensis*	1
	Legionella	*Not Specified*	2		*Burkholderia*	*Burkholderia pseudomallei*	1

	Serratia	*Serratia marcescens*	2
	Xenorhabdus	*Xenorhabdus poinarii*	2
	Photorhabdus	*Photorhabdus luminescens*	2
	Pantoea	*Pantoea stewartii*	2
	Pantoea	*Pantoea sp. BL1*	2
	Pantoea	*Pantoea sp. AS-PWVM4*	2
	Pantoea	*Pantoea sp. A4*	2
	Not Specified	*Not Specified*	2
	Enterobacter	*Enterobacter cancerogenus*	2
Spores_8hai	*Cronobacter*	*Cronobacter sakazakii*	2
	Thiocapsa	*Thiocapsa sp. KS1*	2
	Cellvibrio	*Cellvibrio japonicus*	2
	Azoarcus	*Not Specified*	2
	Thiomonas	*Thiomonas sp. CB2*	2
	Not Specified	*Not Specified*	2
	Cupriavidus	*Cupriavidus metallidurans*	2
	Burkholderia	*Not Specified*	2
	Bordetella	*Bordetella pertussis*	2
	Bordetella	*Bordetella ansorpii*	2
	Not Specified	*Not Specified*	2
	Azospirillum	*Azospirillum brasilense*	2
	Gluconobacter	*Gluconobacter frateurii*	2
	Methylobacterium	*Methylobacterium mesophilicum*	2
	Phenylobacterium	*Phenylobacterium sp. Root700*	2
	Rhizobium	*Rhizobium sp. Leaf262*	2
	Shigella	*Shigella flexneri*	2
	Ralstonia	*Ralstonia solanacearum*	2
	Chlorobium	*Chlorobium chlorochromatii*	1
	Not Specified	*bacterium F082*	1
	Not Specified	*Candidatus Uhrbacteria bacterium GW2011_GWF2_39_13*	1
	Not Specified	*uncultured bacterium 5G12*	1
	Not Specified	*uncultured bacterium 125003-E23*	1
	Not Specified	*Entomoplasmatales bacterium EntAcro1*	1
	Pseudoflavonifractor	*Pseudoflavonifractor capillosus*	1
Spores_8hai	*Ruminiclostridium*	*[Clostridium] cellulosi*	1

	Burkholderia	*Burkholderia sp. lig30*	1
	Burkholderia	*Burkholderia sp. RPE67*	1
	Burkholderia	*Burkholderia stabilis*	1
	Burkholderia	*Burkholderia multivorans*	1
	Bordetella	*Bordetella petrii*	1
	Bordetella	*Bordetella bronchiseptica*	1
	Achromobacter	*Achromobacter xylosoxidans*	1
	Achromobacter	*Not Specified*	1
	Not Specified	*uncultured alpha proteobacterium HF0070_14E07*	1
Spores_8hai	*Sphingomonas*	*Sphingomonas sp. MEA3-1*	1
	Novosphingobium	*Novosphingobium sp. PP1Y*	1
	Not Specified	*uncultured Rhodospirillales bacterium HF4000_24M03*	1
	Azospirillum	*Azospirillum lipoferum*	1
	Roseomonas	*Roseomonas sp. M3*	1
	Ruegeria	*Ruegeria atlantica*	1
	Donghicola	*Donghicola sp. KarMa*	1
	Xanthobacter	*Xanthobacter autotrophicus*	1
	Rhizobium	*Rhizobium sp. 2MFCol3.1*	1
	Agrobacterium	*Agrobacterium tumefaciens*	1
	Mesorhizobium	*Mesorhizobium sp. LSHC422A00*	1
	Methylobacterium	*Not Specified*	1
	Rhodomicrobium	*Not Specified*	1
	Bradyrhizobium	*Bradyrhizobium sp. S23321*	1
	Bradyrhizobium	*Bradyrhizobium sp. DOA1*	1
	Not Specified	*Not Specified*	1
	Methylocella	*Methylocella silvestris*	1
	Candidatus Pelagibacter	*Candidatus Pelagibacter sp. HTCC7211*	1
	Not Specified	*Caulobacterales bacterium 68-7*	1
	Not Specified	*Caulobacteraceae bacterium PMMR1*	1
	Phenylobacterium	*Phenylobacterium sp. SCN 70-31*	1
	Phenylobacterium	*Phenylobacterium sp. RIFCSPHIGHO2_01_FULL_70_10*	1
	Phenylobacterium	*Phenylobacterium sp. RIFCSPHIGHO2_01_FULL_69_31*	1
	Caulobacter	*Caulobacter vibrioides*	1
	Caulobacter	*Caulobacter sp. UNC358MFTsu5.1*	1
Spores_8hai	*Asticcacaulis*	*Not Specified*	1

	Clostridioides	*Clostridioides difficile*	1		*Chlamydia*	*Chlamydia trachomatis*	1
	Clostridium	*uncultured Clostridium sp.*	1		*Sporocytophaga*	*Sporocytophaga myxococcoides*	1
	Streptococcus	*Streptococcus suis*	1		*Chitinophaga*	*Chitinophaga pinensis*	1
	Streptococcus	*Streptococcus agalactiae*	1		*Sanguibacteroides*	*Sanguibacteroides justesenii*	1
	Lactobacillus	*Lactobacillus fermentum*	1		*Not Specified*	*Acidobacteria bacterium 13_1_20CM_3_53_8*	1

Abundance of bacteria species identified in the metatranscriptome of sample derived from Spores 8hs.

Library		Transcripts ≥5	
	Genus	Species	Transcripts
Spores_16hai	*Fusarium*	*Not specified*	46
	Aspergillus	*Not specified*	31
	Not specified	*Not specified*	22
	Fusarium	*Fusarium poae*	21
	Not specified	*Not specified*	21
	Fusarium	*Not specified*	18
	Fusarium	*Fusarium langsethiae*	11
	Not specified	*Not specified*	9
	Aspergillus	*Aspergillus flavus*	9
	Fusarium	*Fusarium avenaceum*	8
	Fusarium	*Fusarium pseudograminearum*	8
	Not specified	*Not specified*	8
	Rhizoctonia	*Rhizoctonia solani*	6
	Fusarium	*Fusarium graminearum*	5
	Fusarium	*Fusarium sp. FIESC_5*	5
	Not specified	*Not specified*	5
	Not specified	*Not specified*	5
	Fusarium	*Fusarium oxysporum*	5
	Aspergillus	*Aspergillus oryzae*	5

Library		Transcripts <5	
	Genus	Species	Transcripts
Spores_16hai	*Fusarium*	*Not specified*	4
	Cylindrobasidium	*Cylindrobasidium torrendii*	4
	Gymnopus	*Gymnopus luxurians*	4
	Hypsizygus	*Hypsizygus marmoreus*	4
	Exidia	*Exidia glandulosa*	4
	Not specified	*Not specified*	4
	Ustilago	*Ustilago maydis*	3
	Puccinia	*Puccinia striiformis*	3
	Serpula	*Serpula lacrymans*	3
	Lentinula	*Lentinula edodes*	3
	Gelatoporia	*Gelatoporia subvermispora*	3
	Fusarium	*Nectria haematococca*	3
	Not specified	*Not specified*	3

Library		Transcripts <5	
	Genus	Species	Transcripts
Spores_16hai	*Pseudozyma*	*Pseudozyma hubeiensis*	1
	Malassezia	*Malassezia globosa*	1
	Wallemia	*Wallemia mellicola*	1
	Tremella	*Tremella mesenterica*	1
	Jaapia	*Jaapia argillacea*	1
	Not specified	*Not specified*	1
	Pisolithus	*Not specified*	1
	Paxillus	*Paxillus rubicundulus*	1
	Coniophora	*Coniophora puteana*	1
	Piloderma	*Piloderma croceum*	1
	Galerina	*Galerina marginata*	1
	Pleurotus	*Pleurotus ostreatus*	1
	Not specified	*Not specified*	1
	Fistulina	*Fistulina hepatica*	1
	Hebeloma	*Hebeloma cylindrosporum*	1
	Leucoagaricus	*Leucoagaricus sp. SymC.cos*	1
Spores_16hai	*Agaricus*	*Agaricus bisporus*	1
	Sistotremastrum	*Sistotremastrum niveocremeum*	1
	Phlebiopsis	*Phlebiopsis gigantea*	1
	Laetiporus	*Laetiporus sulphureus*	1
	Sanghuangporus	*Sanghuangporus baumii*	1
	Gloeophyllum	*Gloeophyllum trabeum*	1
	Auricularia	*Auricularia subglabra*	1
	Not specified	*Not specified*	1
	Not specified	*Not specified*	1
	Naumovozyma	*Naumovozyma dairenensis*	1
	Clavispora	*[Candida] auris*	1
	Clavispora	*Clavispora lusitaniae*	1
	Yamadazyma	*[Candida] tenuis*	1
	Candida	*Candida tropicalis*	1
	Madurella	*Madurella mycetomatis*	1
	Diaporthe	*Diaporthe ampelina*	1
	Tolypocladium	*Tolypocladium ophioglossoides*	1
	Neonectria	*Neonectria ditissima*	1

	Not specified	*Not specified*	3
	Colletotrichum	*Colletotrichum gloeosporioides*	3
	Melanopsichium	*Melanopsichium pennsylvanicum*	2
	Mixia	*Mixia osmundae*	2
	Rhizopogon	*Rhizopogon vinicolor*	2
	Scleroderma	*Scleroderma citrinum*	2
	Fibulorhizoctonia	*Fibulorhizoctonia sp. CBS 109695*	2
	Plicaturopsis	*Plicaturopsis crispa*	2
	Schizophyllum	*Schizophyllum commune*	2
	Grifola	*Grifola frondosa*	2
	Amanita	*Amanita muscaria*	2
	Fibroporia	*Fibroporia radiculosa*	2
	Phanerochaete	*Phanerochaete carnosa*	2
	Punctularia	*Punctularia strigosozonata*	2
	Fusarium	*Fusarium mangiferae*	2
Spores_16hai	*Fusarium*	*Fusarium cyanostomum*	2
	Not specified	*Not specified*	2
	Colletotrichum	*Colletotrichum orbiculare*	2
	Not specified	*Not specified*	2
	Not specified	*Not specified*	2
	Linderina	*Linderina pennispora*	1
	Fusarium	*Fusarium verticillioides*	1
	Fusarium	*Fusarium fujikuroi*	1
	Trichoderma	*Trichoderma virens*	1
	Trichoderma	*Not specified*	1
	Not specified	*Not specified*	1
	Pochonia	*Pochonia chlamydosporia*	1
	Moelleriella	*Moelleriella libera*	1
	Colletotrichum	*Colletotrichum salicis*	1
	Not specified	*Not specified*	1
	Sphaerulina	*Sphaerulina musiva*	1
	Coniosporium	*Coniosporium apollinis*	1
	Talaromyces	*Talaromyces marneffei*	1
	Penicillium	*Not specified*	1
Spores_16hai	*Aspergillus*	*Aspergillus fumigatus*	1
	Aspergillus	*Aspergillus fischeri*	1
	Aspergillus	*Aspergillus brasiliensis*	1
	Not specified	*Not specified*	1
	Not specified	*Not specified*	1
	Not specified	*Not specified*	1
	Not specified	*Not specified*	1
	Gonapodya	*Gonapodya prolifera*	1

Supplementary Table 6. Abundance of fungal species identified in the metatranscriptome of sample derived from Spores 16hs.

Library		Transcripts ≥5		Library		Transcripts <5	
	Genus	Species	Transcripts		Genus	Species	Transcripts
	Not Specified	*Not Specified*	1244		*Vibrio*	*Vibrio parahaemolyticus*	2
	Pantoea	*Pantoea dispersa*	363		*Pseudomonas*	*Pseudomonas amygdali*	2
	Pseudomonas	*Not Specified*	265		*Providencia*	*Providencia stuartii*	2
	Pantoea	*Not Specified*	162		*Photorhabdus*	*Photorhabdus luminescens*	2
	Not Specified	*Not Specified*	123		*Pantoea*	*Pantoea vagans*	2
	Not Specified	*Not Specified*	95		*Cedecea*	*Cedecea davisae*	2
	Not Specified	*Not Specified*	82		*Hymenobacter*	*Hymenobacter sp. CCM 8649*	1
	Escherichia	*Escherichia coli*	81		*Sporocytophaga*	*Sporocytophaga myxococcoides*	1
	Pantoea	*Pantoea rwandensis*	74		*Not Specified*	*bacterium P201*	1
Spores_16hai	*Erwinia*	*Erwinia sp. Leaf53*	61	Spores_16hai	*Not Specified*	*uncultured bacterium A1Q1_fos_4*	1
	Enterobacter	*Enterobacter cancerogenus*	56		*Ruminiclostridium*	*[Clostridium] leptum*	1
	Pantoea	*Pantoea ananatis*	52		*Desulfotomaculum*	*Desulfotomaculum geothermicum*	1
	Erwinia	*Erwinia persicina*	48		*Eubacterium*	*Eubacterium ventriosum*	1
	Pseudomonas	*Pseudomonas putida*	42		*Streptococcus*	*Streptococcus agalactiae*	1
	Not Specified	*bacteria symbiont BFo1 of Frankliniella occidentalis*	41		*Lactobacillus*	*Lactobacillus plantarum*	1
	Not Specified	*Not Specified*	40		*Staphylococcus*	*Staphylococcus saprophyticus*	1
	Erwinia	*Not Specified*	32		*Paenibacillus*	*Paenibacillus sp. Soil787*	1
	Pantoea	*Pantoea rodasii*	29		*Paenibacillus*	*Paenibacillus sp. P22*	1
	Not Specified	*Type-E symbiont of Plautia stali*	29		*Paenibacillus*	*Paenibacillus sp. FF9*	1
	Klebsiella	*Klebsiella pneumoniae*	25		*Bacillus*	*Bacillus pumilus*	1
	Not Specified	*Not Specified*	21		*Geomicrobium*	*Geomicrobium sp. JCM 19038*	1
	Erwinia	*Erwinia amylovora*	21		*Rhodococcus*	*Rhodococcus sp. RD6.2*	1
	Stenotrophomonas	*Stenotrophomonas maltophilia*	20		*Bifidobacterium*	*Bifidobacterium adolescentis*	1
	Pantoea	*Pantoea wallisii*	18		*Actinomyces*	*Actinomyces turicensis*	1
	Erwinia	*Erwinia iniecta*	18		*Not Specified*	*Not Specified*	1
	Salmonella	*Salmonella enterica*	16		*Sulfurospirillum*	*Sulfurospirillum arcachonense*	1
Spores_16hai	*Erwinia*	*Erwinia mallotivora*	15	Spores_16hai	*Stenotrophomonas*	*Stenotrophomonas sp. SKA14*	1
	Erwinia	*Erwinia toletana*	11		*Stenotrophomonas*	*Stenotrophomonas rhizophila*	1
	Not Specified	*uncultured bacterium*	10		*Pseudoxanthomonas*	*Pseudoxanthomonas sp. GW2*	1
	Pantoea	*Pantoea sp. IMH*	10		*Not Specified*	*Not Specified*	1
	Pantoea	*Pantoea cypripedii*	10		*Vibrio*	*Vibrio sp. HENC-02*	1
	Erwinia	*Erwinia typographi*	10		*Vibrio*	*Vibrio campbellii*	1
	Burkholderia	*Not Specified*	10		*Salinivibrio*	*Salinivibrio sp. PR5*	1
	Erwinia	*Erwinia billingiae*	10		*Thiothrix*	*Thiothrix caldifontis*	1
	Erwinia	*Erwinia piriflorinigrans*	9		*Not Specified*	*uncultured Pseudomonadales bacterium HF0010_05E14*	1
	Xanthomonas	*Xanthomonas citri*	8		*Pseudomonas*	*Pseudomonas fuscovaginae*	1
	Pseudomonas	*Pseudomonas parafulva*	8		*Pseudomonas*	*Pseudomonas stutzeri*	1

	Pantoea	*Pantoea sp. At-9b*	8
	Pantoea	*Pantoea sp. 1.19*	8
	Erwinia	*Erwinia tasmaniensis*	8
	Erwinia	*Erwinia gerundensis*	8
Spores_16hai	*Stenotrophomonas*	*Not Specified*	7
	Pseudomonas	*Not Specified*	7
	Acinetobacter	*Not Specified*	7
	Edwardsiella	*Edwardsiella piscicida*	7
	Erwinia	*Erwinia sp. ErVv1*	7
	Not Specified	*Type-D symbiont of Plautia stali*	7
	Not Specified	*Type-C symbiont of Plautia stali*	7
	Enterobacter	*Enterobacter cloacae*	7
	Acinetobacter	*Acinetobacter baumannii*	7
	Pseudomonas	*Pseudomonas syringae*	7
	Not Specified	*methanotrophic bacterial endosymbiont of Bathymodiolus sp.*	6
	Pseudomonas	*Pseudomonas savastanoi*	6
	Pseudomonas	*Pseudomonas xanthomarina*	6
	Haemophilus	*Not Specified*	6
Spores_16hai	*Tatumella*	*Tatumella morbirosei*	6
	Pantoea	*Pantoea sp. SM3*	6
	Pantoea	*Pantoea sp. AS-PWVM4*	6
	Erwinia	*Erwinia tracheiphila*	6
	Erwinia	*Candidatus Erwinia dacicola*	6
	Not Specified	*Plautia stali symbiont*	6
	Brucella	*Not Specified*	6
	Serratia	*Serratia marcescens*	5
	Pantoea	*Pantoea stewartii*	5
	Pantoea	*Pantoea sp. BL1*	5
	Klebsiella	*Not Specified*	5
	Ralstonia	*Ralstonia sp. NT80*	5
	Pseudomonas	*Pseudomonas fluorescens*	5

**Library**		**Transcripts <5**	
	**Genus**	**Species**	**Transcripts**
	Serratia	*Serratia symbiotica*	4
	Rouxiella	*Rouxiella badensis*	4
Spores_16hai	*Pantoea*	*Pantoea sp. A4*	4
	Pantoea	*Pantoea eucrina*	4
	Raoultella	*Not Specified*	4
	Burkholderia	*Burkholderia multivorans*	4
	Not Specified	*Not Specified*	4
	Pseudomonas	*Pseudomonas sp. NBRC 111138*	1
	Pseudomonas	*Pseudomonas sp. NBRC 111133*	1
	Pseudomonas	*Pseudomonas sp. NBRC 111124*	1
	Pseudomonas	*Pseudomonas sp. GM30*	1
Spores_16hai	*Pseudomonas*	*Pseudomonas sp. CCOS 191*	1
	Pseudomonas	*Pseudomonas sp. Bc-h*	1
	Pseudomonas	*Pseudomonas japonica*	1
	Pseudomonas	*Pseudomonas tolaasii*	1
	Pseudomonas	*Pseudomonas protegens*	1
	Pseudomonas	*Pseudomonas mucidolens*	1
	Pseudomonas	*Pseudomonas entomophila*	1
	Pseudomonas	*Pseudomonas coleopterorum*	1
	Pseudomonas	*Pseudomonas chlororaphis*	1
	Pseudomonas	*Pseudomonas pseudoalcaligenes*	1
	Pseudomonas	*Pseudomonas abietaniphila*	1
	Not Specified	*Not Specified*	1
	Psychrobacter	*Psychrobacter sp. AntiMn-1*	1
	Acinetobacter	*Acinetobacter towneri*	1
Spores_16hai	*Acinetobacter*	*Acinetobacter sp. C15*	1
	Acinetobacter	*Acinetobacter seohaensis*	1
	Acinetobacter	*Not Specified*	1
	Marinospirillum	*Marinospirillum alkaliphilum*	1
	Legionella	*Legionella londiniensis*	1
	Yersinia	*Yersinia nurmii*	1
	Yersinia	*Yersinia mollaretii*	1
	Yersinia	*Yersinia intermedia*	1
	Serratia	*Serratia plymuthica*	1
	Serratia	*Serratia odorifera*	1
	Serratia	*Serratia liquefaciens*	1
	Rahnella	*Rahnella aquatilis*	1
	Rahnella	*Not Specified*	1
	Nissabacter	*Nissabacter archeti*	1
	Not Specified	*Not Specified*	1
	Pectobacterium	*Pectobacterium atrosepticum*	1
	Pectobacterium	*Not Specified*	1
Spores_16hai	*Dickeya*	*Dickeya paradisiaca*	1
	Dickeya	*Dickeya dianthicola*	1
	Dickeya	*Dickeya chrysanthemi*	1
	Dickeya	*Not Specified*	1
	Xenorhabdus	*Xenorhabdus nematophila*	1

	Cutibacterium	Cutibacterium acnes	4
	Pseudomonas	Pseudomonas sp. P482	3
	Pseudomonas	Pseudomonas sp. NFR16	3
	Pseudomonas	Pseudomonas sp. NFPP07	3
	Pseudomonas	Pseudomonas sp. NBRC 111131	3
	Pseudomonas	Pseudomonas sp. CB1	3
	Pseudomonas	Pseudomonas sp. BMS12	3
	Pseudomonas	Pseudomonas graminis	3
	Pseudomonas	Pseudomonas cremoricolorata	3
	Pseudomonas	Pseudomonas aeruginosa	3
Spores_16hai	Not Specified	Not Specified	3
	Haemophilus	Haemophilus influenzae	3
	Aggregatibacter	Aggregatibacter aphrophilus	3
	Halomonas	Halomonas titanicae	3
	Pectobacterium	Pectobacterium carotovorum	3
	Photorhabdus	Photorhabdus asymbiotica	3
	Hafnia	Hafnia alvei	3
	Pantoea	Pantoea alhagi	3
	Erwinia	Erwinia sp. 9145	3
	Shigella	Shigella sonnei	3
	Klebsiella	Klebsiella aerogenes	3
	Izhakiella	Izhakiella capsodis	3
	Enterobacter	Enterobacter ludwigii	3
Spores_16hai	Marinobacter	Marinobacter sp. T13-3	3
	Not Specified	uncultured beta proteobacterium CBNPD1 BAC clone 578	3
	Curvibacter	Curvibacter putative symbiont of Hydra magnipapillata	3
	Novosphingobium	Novosphingobium sp. PP1Y	3
	Roseibium	Roseibium sp. TrichSKD4	3
	Rhizobium	Rhizobium sp. NT-26	3
	Brucella	Brucella pinnipedialis	3
	Riemerella	Riemerella anatipestifer	3
	Pseudomonas	Pseudomonas plecoglossicida	3
	Pantoea	Pantoea agglomerans	3
	Cronobacter	Cronobacter sakazakii	3
	Not Specified	uncultured bacterium 5H7	2
	Pseudoflavonifractor	Pseudoflavonifractor capillosus	2
	Streptococcus	Streptococcus mutans	2
	Staphylococcus	Staphylococcus hominis	2
Spores_16hai	Mycobacterium	Mycobacterium abscessus	2

	Xenorhabdus	Xenorhabdus cabanillasii	1
	Xenorhabdus	Xenorhabdus bovienii	1
	Providencia	Providencia heimbachae	1
	Not Specified	Not Specified	1
	Hafnia	Hafnia paralvei	1
	Edwardsiella	Edwardsiella tarda	1
	Tatumella	Tatumella saanichensis	1
	Phaseolibacter	Phaseolibacter flectens	1
	Pantoea	Pantoea sp. RIT-PI-b	1
	Pantoea	Pantoea sp. PSNIH2	1
Spores_16hai	Pantoea	Pantoea sp. OV426	1
	Pantoea	Pantoea sp. NGS-ED-1003	1
	Pantoea	Pantoea sp. GM01	1
	Pantoea	Pantoea deleyi	1
	Pantoea	Pantoea calida	1
	Erwinia	Erwinia teleogrylli	1
	Erwinia	Erwinia pyrifoliae	1
	Erwinia	Erwinia psidii	1
	Erwinia	Erwinia oleae	1
	Not Specified	Not Specified	1
	Shigella	Shigella flexneri	1
	Shigella	Not Specified	1
	Raoultella	Raoultella terrigena	1
Spores_16hai	Lelliottia	Lelliottia amnigena	1
	Leclercia	Leclercia adecarboxylata	1
	Kosakonia	Kosakonia oryziphila	1
	Kosakonia	Kosakonia oryzendophytica	1
	Kluyvera	Kluyvera intermedia	1
	Klebsiella	Klebsiella variicola	1
	Klebsiella	Klebsiella quasipneumoniae	1
	Franconibacter	Franconibacter helveticus	1
	Escherichia	Not Specified	1
	Enterobacter	Enterobacter timonensis	1
	Enterobacter	Enterobacter kobei	1
	Enterobacter	Enterobacter sp. MGH 16	1
	Cronobacter	Cronobacter universalis	1
	Cronobacter	Not Specified	1
	Citrobacter	Citrobacter koseri	1
Spores_16hai	Citrobacter	Citrobacter freundii complex	1

	Xanthomonas	*Xanthomonas fuscans*	2
	Not Specified	*Not Specified*	2
	Vibrio	*Not Specified*	2
	Pseudomonas	*Pseudomonas syringae group genomosp. 2*	2
	Pseudomonas	*Pseudomonas cichorii*	2
	Pseudomonas	*Pseudomonas sp. Leaf127*	2
	Pseudomonas	*Pseudomonas sp. FGI182*	2
	Pseudomonas	*Pseudomonas sp. DRA525*	2
	Pseudomonas	*Pseudomonas sp. Chol1*	2
	Pseudomonas	*Pseudomonas sp. 10-1B*	2
	Pseudomonas	*Not Specified*	2
	Pseudomonas	*Not Specified*	2
Spores_16hai	*Acinetobacter*	*Acinetobacter pittii*	2
	Basfia	*[Mannheimia] succiniciproducens*	2
	Yersinia	*Yersinia pestis*	2
	Yersinia	*Yersinia enterocolitica*	2
	Yersinia	*Not Specified*	2
	Rouxiella	*Rouxiella chamberiensis*	2
	Ewingella	*Ewingella americana*	2
	Xenorhabdus	*Xenorhabdus khoisanae*	2
	Tatumella	*Not Specified*	2
	Pantoea	*Pantoea septica*	2
	Pantoea	*Pantoea gaviniae*	2
	Shimwellia	*Shimwellia blattae*	2
	Raoultella	*Raoultella planticola*	2
	Raoultella	*Raoultella ornithinolytica*	2
Spores_16hai	*Klebsiella*	*Klebsiella oxytoca*	2
	Escherichia	*Escherichia vulneris*	2
	Enterobacter	*Not Specified*	2
	Cronobacter	*Cronobacter turicensis*	2
	Burkholderia	*Burkholderia pseudomallei*	2
	Burkholderia	*Burkholderia mallei*	2
	Burkholderia	*Not Specified*	2
	Streptococcus	*Streptococcus pneumoniae*	2

	Citrobacter	*Citrobacter amalonaticus*	1
	Citrobacter	*Not Specified*	1
	Cedecea	*Cedecea sp. NFIX57*	1
	Cedecea	*Cedecea neteri*	1
	Buttiauxella	*Buttiauxella brennerae*	1
	Buttiauxella	*Buttiauxella agrestis*	1
	Thiocapsa	*Thiocapsa sp. KS1*	1
	Rheinheimera	*Rheinheimera nanhaiensis*	1
	Teredinibacter	*Teredinibacter turnerae*	1
	Pseudoalteromonas	*Pseudoalteromonas luteoviolacea*	1
	Not Specified	*uncultured beta proteobacterium HF0130_04F21*	1
	Not Specified	*uncultured beta proteobacterium HF0010_04H24*	1
Spores_16hai	*Thiomonas*	*Thiomonas sp. CB2*	1
	Acidovorax	*Acidovorax sp. CF316*	1
	Paraburkholderia	*Paraburkholderia kururiensis*	1
	Paraburkholderia	*Candidatus Paraburkholderia schumannianae*	1
	Burkholderia	*Burkholderia sp. TSV86*	1
	Burkholderia	*Burkholderia mallei*	1
	Burkholderia	*Burkholderia sp. RPE67*	1
	Not Specified	*Not Specified*	1
	Taylorella	*Taylorella asinigenitalis*	1
	Candidimonas	*Candidimonas bauzanensis*	1
	Achromobacter	*Not Specified*	1
	Not Specified	*Not Specified*	1
	Sphingomonas	*Sphingomonas paucimobilis*	1
	Azospirillum	*Azospirillum lipoferum*	1
Spores_16hai	*Azospirillum*	*Azospirillum brasilense*	1
	Komagataeibacter	*Komagataeibacter nataicola*	1
	Donghicola	*Donghicola sp. KarMa*	1
	Liberibacter	*Candidatus Liberibacter solanacearum*	1
	Brucella	*Brucella suis*	1
	Fulvimarina	*Fulvimarina pelagi*	1
	Not Specified	*Not Specified*	1
	Chlamydia	*Chlamydia muridarum*	1

Abundance of bacteria species identified in the metatranscriptome of sample derived from Spores 16hs.

Library		Transcripts ≥5		Library		Transcripts <5	
	Genus	Species	Transcripts		Genus	Species	Transcripts
	Colletotrichum	*Colletotrichum gloeosporioides*	18111		*Not specified*	*Not specified*	4
	Colletotrichum	*Not specified*	928		*Hypocrella*	*Hypocrella siamensis*	4
	Acremonium	*Acremonium chrysogenum*	440		*Not specified*	*Not specified*	4
	Fusarium	*Nectria haematococca*	334		*Not specified*	*Not specified*	4
	Neonectria	*Neonectria ditissima*	229		*Not specified*	*Not specified*	4
	Fusarium	*Not specified*	189		*Verticillium*	*Verticillium alfalfae*	4
	Not specified	*Not specified*	167		*Pseudogymnoascus*	*Pseudogymnoascus sp. VKM F-4518 (FW-2643)*	4
	Trichoderma	*Not specified*	151		*Not specified*	*Not specified*	4
	Not specified	*Not specified*	151		*Not specified*	*Not specified*	4
	Colletotrichum	*Colletotrichum incanum*	142		*Pyrenophora*	*Pyrenophora teres*	4
	Colletotrichum	*Colletotrichum orbiculare*	133		*Bipolaris*	*Bipolaris maydis*	4
	Purpureocillium	*Purpureocillium lilacinum*	129		*Epicoccum*	*Epicoccum nigrum*	4
	Colletotrichum	*Colletotrichum chlorophyti*	128		*Not specified*	*Not specified*	4
	Stachybotrys	*Not specified*	110		*Trichophyton*	*Not specified*	4
	Fusarium	*Fusarium avenaceum*	103		*Penicillium*	*Penicillium steckii*	4
	Colletotrichum	*Colletotrichum higginsianum*	89		*Penicillium*	*Penicillium italicum*	4
	Hirsutella	*Hirsutella minnesotensis*	89		*Aspergillus*	*Aspergillus wentii*	4
	Torrubiella	*Torrubiella hemipterigena*	86		*Aspergillus*	*Aspergillus aculeatus*	4
	Tolypocladium	*Tolypocladium ophioglossoides*	81		*Exophiala*	*Exophiala spinifera*	4
	Not specified	*Not specified*	71		*Dactylellina*	*Dactylellina haptotyla*	4
	Colletotrichum	*Colletotrichum orchidophilum*	67		*Not specified*	*Not specified*	4
	Metarhizium	*Not specified*	66		*Rasamsonia*	*Rasamsonia emersonii*	3
	Drechmeria	*Drechmeria coniospora*	64		*Byssochlamys*	*Byssochlamys spectabilis*	3
	Candida	*Candida parapsilosis*	64		*Talaromyces*	*Talaromyces stipitatus*	3
	Fusarium	*Not specified*	61		*Penicillium*	*Penicillium subrubescens*	3
	Fusarium	*Fusarium oxysporum*	59		*Penicillium*	*Penicillium expansum*	3
Spores_48hai	*Escovopsis*	*Escovopsis weberi*	59	Spores_48hai	*Penicillium*	*Penicillium coprophilum*	3
	Colletotrichum	*Colletotrichum sublineola*	57		*Aspergillus*	*Aspergillus sydowii*	3
	Not specified	*Not specified*	57		*Aspergillus*	*Aspergillus clavatus*	3
	Colletotrichum	*Colletotrichum tofieldiae*	54		*Not specified*	*Not specified*	3

	Moelleriella	*Moelleriella libera*	53
	Pochonia	*Pochonia chlamydosporia*	52
	Ustilaginoidea	*Ustilaginoidea virens*	51
	Cordyceps	*Cordyceps confragosa*	50
	Claviceps	*Claviceps purpurea*	50
	Not specified	*Not specified*	46
	Not specified	*Not specified*	44
	Colletotrichum	*Colletotrichum graminicola*	44
	Pestalotiopsis	*Pestalotiopsis fici*	42
	Ophiocordyceps	*Ophiocordyceps unilateralis*	42
	Isaria	*Isaria fumosorosea*	42
	Verticillium	*Not specified*	40
	Ophiocordyceps	*Ophiocordyceps sinensis*	40
	Fusarium	*Fusarium poae*	39
	Metarhizium	*Metarhizium album*	37
	Stachybotrys	*Stachybotrys chartarum*	36
	Not specified	*Not specified*	35
	Scedosporium	*Scedosporium apiospermum*	34
	Trichoderma	*Trichoderma atroviride*	32
	Colletotrichum	*Colletotrichum salicis*	31
	Trichoderma	*Trichoderma gamsii*	31
	Trichoderma	*Trichoderma virens*	31
	Phaeoacremonium	*Phaeoacremonium minimum*	31
	Pseudomassariella	*Pseudomassariella vexata*	29
	Metarhizium	*Metarhizium rileyi*	29
	Not specified	*Not specified*	29
	Not specified	*Not specified*	28
	Cordyceps	*Cordyceps militaris*	28
	Not specified	*Not specified*	28
	Colletotrichum	*Colletotrichum fioriniae*	26
	Eutypa	*Eutypa lata*	26
	Fusarium	*Fusarium verticillioides*	25
	Colletotrichum	*Colletotrichum nymphaeae*	24
	Metarhizium	*Metarhizium acridum*	24
	Coniochaeta	*Coniochaeta ligniaria*	23
	Pleurotus	*Pleurotus ostreatus*	3
	Gymnopus	*Gymnopus luxurians*	3
	Not specified	*Not specified*	3
	Not specified	*Not specified*	3
	Not specified	*Not specified*	3
	Not specified	*Not specified*	3
	Neurospora	*Neurospora crassa*	3
	Not specified	*Not specified*	3
	Fusarium	*Not specified*	3
	Metarhizium	*Metarhizium robertsii*	3
	Metarhizium	*Metarhizium guizhouense*	3
	Pseudogymnoascus	*Pseudogymnoascus sp. VKM F-4520 (FW-2644)*	3
	Pseudogymnoascus	*Pseudogymnoascus sp. VKM F-3775*	3
	Phialocephala	*Phialocephala scopiformis*	3
	Glonium	*Glonium stellatum*	3
	Cenococcum	*Cenococcum geophilum*	3
	Stemphylium	*Stemphylium lycopersici*	3
	Setosphaeria	*Setosphaeria turcica*	3
	Alternaria	*Alternaria alternata*	3
	Ascochyta	*Ascochyta rabiei*	3
	Sphaerulina	*Sphaerulina musiva*	3
	Dothistroma	*Dothistroma septosporum*	3
	Verruconis	*Verruconis gallopava*	3
	Neofusicoccum	*Neofusicoccum parvum*	3
	Coccidioides	*Coccidioides immitis*	3
	Ascosphaera	*Ascosphaera apis*	3
	Phaeomoniella	*Phaeomoniella chlamydospora*	3
	Phialophora	*Phialophora attae*	3
	Exophiala	*Exophiala mesophila*	3
	Capronia	*Capronia coronata*	3
	Pyronema	*Pyronema omphalodes*	3
	Sporothrix	*Sporothrix schenckii*	3
	Coprinopsis	*Coprinopsis cinerea*	2
	Galerina	*Galerina marginata*	2
	Not specified	*Not specified*	2

Spores_48hai	*Fusarium*	*Not specified*	22
	Daldinia	*Daldinia sp. EC12*	22
	Microdochium	*Microdochium bolleyi*	22
	Not specified	*Not specified*	20
	Not specified	*Not specified*	20
	Not specified	*fungal sp. No.14919*	20
	Valsa	*Valsa mali*	20
	Clonostachys	*Clonostachys rosea*	20
	Not specified	*Not specified*	19
	Fusarium	*Fusarium langsethiae*	19
	Ophiostoma	*Ophiostoma piceae*	18
	Diaporthe	*Diaporthe helianthi*	17
	Oidiodendron	*Oidiodendron maius*	16
	Fusarium	*Fusarium mangiferae*	15
	Hypoxylon	*Hypoxylon sp. CI-4A*	15
	Magnaporthe	*Magnaporthe oryzae*	15
	Gaeumannomyces	*Gaeumannomyces tritici*	15
	Aspergillus	*Not specified*	15
	Trichoderma	*Trichoderma guizhouense*	14
	Rosellinia	*Rosellinia necatrix*	14
	Hypoxylon	*Not specified*	14
	Thielavia	*Thielavia terrestris*	14
	Candida	*Not specified*	14
	Colletotrichum	*Colletotrichum simmondsii*	13
	Fusarium	*Fusarium pseudograminearum*	13
	Stachybotrys	*Stachybotrys chlorohalonata*	13
	Sporothrix	*Not specified*	13
	Podospora	*Podospora anserina*	13
	Madurella	*Madurella mycetomatis*	12
	Thielaviopsis	*Thielaviopsis punctulata*	12
	Phialocephala	*Phialocephala subalpina*	12
	Verticillium	*Verticillium longisporum*	11
	Not specified	*Not specified*	11
	Fusarium	*Fusarium graminearum*	11
Spores_48hai	*Fusarium*	*Fusarium acuminatum*	2
	Metarhizium	*Metarhizium majus*	2
	Bipolaris	*Not specified*	2
	Not specified	*Not specified*	2
	Rhizopogon	*Rhizopogon vinicolor*	2
	Fibroporia	*Fibroporia radiculosa*	2
	Neolentinus	*Neolentinus lepideus*	2
	Botryobasidium	*Botryobasidium botryosum*	2
	Lachancea	*Lachancea thermotolerans*	2
	Not specified	*Not specified*	2
	Hypoxylon	*Hypoxylon sp. CO27-5*	2
	Not specified	*Not specified*	2
	Not specified	*Not specified*	2
	Not specified	*Not specified*	2
	Pseudogymnoascus	*Pseudogymnoascus sp. VKM F-4281 (FW-2241)*	2
	Pseudogymnoascus	*Pseudogymnoascus sp. 23342-1-I1*	2
	Pseudogymnoascus	*Pseudogymnoascus sp. 03VT05*	2
	Erysiphe	*Erysiphe necator*	2
	Not specified	*Not specified*	2
	Clohesyomyces	*Clohesyomyces aquaticus*	2
	Baudoinia	*Baudoinia panamericana*	2
	Zymoseptoria	*Not specified*	2
	Mycosphaerella	*Mycosphaerella eumusae*	2
	Diplodia	*Diplodia corticola*	2
	Diplodia	*Not specified*	2
	Xylona	*Xylona heveae*	2
	Umbilicaria	*Umbilicaria pustulata*	2
	Not specified	*Not specified*	2
	Emergomyces	*Emergomyces pasteurianus*	2
	Not specified	*Not specified*	2
	Talaromyces	*Talaromyces islandicus*	2
	Penicillium	*Penicillium roqueforti*	2
	Penicillium	*Penicillium griseofulvum*	2
	Penicillium	*Penicillium brasilianum*	2

	Beauveria	*Beauveria bassiana*	11		*Penicillium*	*Penicillium arizonense*	2
	Diaporthe	*Diaporthe ampelina*	11		*Penicilliopsis*	*Penicilliopsis zonata*	2
	Thermothelomyces	*Thermothelomyces thermophila*	11		*Aspergillus*	*Aspergillus versicolor*	2
	Chaetomium	*Chaetomium thermophilum*	11		*Aspergillus*	*Aspergillus parasiticus*	2
	Cordyceps	*Cordyceps brongniartii*	10		*Aspergillus*	*Aspergillus nomius*	2
	Grosmannia	*Grosmannia clavigera*	10		*Aspergillus*	*Aspergillus fischeri*	2
	Trichoderma	*Trichoderma reesei*	9		*Phialophora*	*Phialophora americana*	2
	Diaporthe	*Not specified*	9		*Fonsecaea*	*Not specified*	2
	Penicillium	*Not specified*	9		*Exophiala*	*Exophiala xenobiotica*	2
	Neurospora	*Neurospora tetrasperma*	9		*Exophiala*	*Exophiala dermatitidis*	2
	Pseudogymnoascus	*Not specified*	9		*Exophiala*	*Exophiala aquamarina*	2
	Fusarium	*Fusarium fujikuroi*	8		*Tuber*	*Tuber melanosporum*	2
	Chaetomium	*Chaetomium globosum*	8		*Drechslerella*	*Drechslerella stenobrocha*	2
	Ceratocystis	*Ceratocystis platani*	8		*Arthrobotrys*	*Arthrobotrys oligospora*	2
	Not specified	*Not specified*	8		*Not specified*	*Not specified*	2
	Rhizoctonia	*Rhizoctonia solani*	8		*Bipolaris*	*Bipolaris oryzae*	1
	Trichoderma	*Trichoderma harzianum*	7		*Leptosphaeria*	*Leptosphaeria maculans*	1
	Cordyceps	*Not specified*	7		*Not specified*	*Not specified*	1
	Hypoxylon	*Hypoxylon sp. EC38*	7		*Not specified*	*fungal sp. No.11243*	1
	Neurospora	*Not specified*	7		*Basidiobolus*	*Basidiobolus meristosporus*	1
	Rhynchosporium	*Not specified*	7		*Not specified*	*Not specified*	1
	Marssonina	*Marssonina brunnea*	7		*Absidia*	*Absidia repens*	1
	Metarhizium	*Metarhizium anisopliae*	6		*Rhizophagus*	*Rhizophagus irregularis*	1
	Metarhizium	*Metarhizium brunneum*	6		*Ustilago*	*Ustilago maydis*	1
	Glarea	*Glarea lozoyensis*	6		*Not specified*	*Not specified*	1
	Sordaria	*Sordaria macrospora*	6		*Melampsora*	*Melampsora larici-populina*	1
	Sclerotinia	*Sclerotinia sclerotiorum*	6		*Mixia*	*Mixia osmundae*	1
Spores_48hai	*Blumeria*	*Blumeria graminis*	6	Spores_48hai	*Trichosporon*	*Trichosporon asahii*	1
	Paraphaeosphaeria	*Paraphaeosphaeria sporulosa*	6		*Jaapia*	*Jaapia argillacea*	1
	Talaromyces	*Talaromyces atroroseus*	6		*Rhizopogon*	*Rhizopogon vesiculosus*	1
	Aspergillus	*Aspergillus terreus*	6		*Not specified*	*Not specified*	1
	Verticillium	*Verticillium dahliae*	5		*Pisolithus*	*Not specified*	1
	Trichoderma	*Trichoderma parareesei*	5		*Paxillus*	*Paxillus rubicundulus*	1
	Fusarium	*Fusarium sp. FIESC_5*	5		*Serpula*	*Serpula lacrymans*	1
	Sporothrix	*Sporothrix insectorum*	5		*Fibulorhizoctonia*	*Fibulorhizoctonia sp. CBS 109695*	1

	Magnaporthiopsis	*Magnaporthiopsis poae*	5
	Not specified	*Not specified*	5
	Stagonospora	*Stagonospora sp. SRC1lsM3a*	5
	Pyrenochaeta	*Pyrenochaeta sp. DS3sAY3a*	5
	Parastagonospora	*Parastagonospora nodorum*	5
	Hortaea	*Hortaea werneckii*	5
	Diplodia	*Diplodia seriata*	5
	Aspergillus	*Aspergillus calidoustus*	5
	Fonsecaea	*Fonsecaea erecta*	5
	Not specified	*Not specified*	5
	Not specified	*Not specified*	5
	Fusarium	*Fusarium proliferatum*	5
	Botrytis	*Botrytis cinerea*	5
		**Transcripts <5**	
	**Genus**	**Species**	**Transcripts**
	Trametes	*Not specified*	1
	Postia	*Postia placenta*	1
	Gelatoporia	*Gelatoporia subvermispora*	1
	Schizopora	*Schizopora paradoxa*	1
	Gloeophyllum	*Gloeophyllum trabeum*	1
	Exidia	*Exidia glandulosa*	1
	Auricularia	*Auricularia auricula-judae*	1
	Not specified	*Nilaparvata lugens yeast-like symbiont*	1
	Kuraishia	*Kuraishia capsulata*	1
	Tetrapisispora	*Tetrapisispora blattae*	1
	Kazachstania	*Kazachstania saulgeensis*	1
	Eremothecium	*Eremothecium cymbalariae*	1
	Wickerhamomyces	*Wickerhamomyces ciferrii*	1
	Komagataella	*Komagataella phaffii*	1
	Metschnikowia	*Metschnikowia bicuspidata*	1
	Geotrichum	*Geotrichum candidum*	1
	Yamadazyma	*[Candida] tenuis*	1
	Candida	*Candida albicans*	1

	Mycena	*Mycena chlorophos*	1
	Hypholoma	*Hypholoma sublateritium*	1
	Schizophyllum	*Schizophyllum commune*	1
	Cylindrobasidium	*Cylindrobasidium torrendii*	1
	Lentinula	*Lentinula edodes*	1
	Hypsizygus	*Hypsizygus marmoreus*	1
	Amanita	*Amanita muscaria*	1
	Leucoagaricus	*Leucoagaricus sp. SymC.cos*	1
	Sistotremastrum	*Not specified*	1
	Stereum	*Stereum hirsutum*	1
	Peniophora	*Peniophora sp. CONT*	1
	Pycnoporus	*Pycnoporus coccineus*	1
	Phlebiopsis	*Phlebiopsis gigantea*	1
	Fomitopsis	*Fomitopsis pinicola*	1
	Trametes	*Trametes pubescens*	1
	Zymoseptoria	*Zymoseptoria tritici*	1
	Pseudocercospora	*Pseudocercospora fijiensis*	1
	Pseudocercospora	*Not specified*	1
	Rachicladosporium	*Not specified*	1
	Coniosporium	*Coniosporium apollinis*	1
	Acidomyces	*Acidomyces richmondensis*	1
	Not specified	*Not specified*	1
	Macrophomina	*Macrophomina phaseolina*	1
	Not specified	*Not specified*	1
	Uncinocarpus	*Uncinocarpus reesii*	1
	Nannizzia	*Nannizzia gypsea*	1
	Microsporum	*Microsporum canis*	1
	Histoplasma	*Histoplasma capsulatum*	1
	Emmonsia	*Emmonsia crescens*	1
	Blastomyces	*Not specified*	1
Spores_48hai	*Talaromyces*	*Talaromyces marneffei*	1
	Penicillium	*Penicillium vulpinum*	1
	Penicillium	*Penicillium oxalicum*	1

	Not specified	*Not specified*	1
	Sporothrix	*Sporothrix brasiliensis*	1
	Magnaporthe	*Not specified*	1
	Not specified	*Not specified*	1
	Tolypocladium	*Tolypocladium inflatum*	1
	Fusarium	*Fusarium ventricosum*	1
Spores_48hai	*Fusarium*	*Not specified*	1
	Fusarium	*Fusarium sp. FN080326*	1
	Fusarium	*Fusarium sambucinum*	1
	Fusarium	*Fusarium foetens*	1
	Fusarium	*Fusarium lichenicola*	1
	Fusarium	*Fusarium aywerte*	1
	Isaria	*Isaria javanica*	1
	Epichloe	*Epichloe festucae*	1
	Not specified	*Bionectriaceae sp. MOS822*	1
	Colletotrichum	*Colletotrichum siamense*	1
	Pseudogymnoascus	*Pseudogymnoascus destructans*	1
	Sclerotinia	*Not specified*	1
	Phialocephala	*Not specified*	1
	Cadophora	*Cadophora finlandica*	1
	Not specified	*Not specified*	1
	Aureobasidium	*Aureobasidium subglaciale*	1
	Aureobasidium	*Aureobasidium pullulans*	1
	Aureobasidium	*Aureobasidium melanogenum*	1
	Aureobasidium	*Not specified*	1
	Batrachochytrium	*Batrachochytrium dendrobatidis*	1
	Penicillium	*Penicillium nordicum*	1
	Penicillium	*Penicillium digitatum*	1
	Penicillium	*Penicillium camemberti*	1
	Penicillium	*Penicillium antarcticum*	1
	Aspergillus	*Aspergillus udagawae*	1
	Aspergillus	*Aspergillus ruber*	1
	Aspergillus	*Aspergillus ochraceoroseus*	1
	Aspergillus	*Aspergillus niger*	1
	Aspergillus	*Aspergillus nidulans*	1
	Aspergillus	*Aspergillus glaucus*	1
	Aspergillus	*Aspergillus fumigatus*	1
	Aspergillus	*Aspergillus flavus*	1
	Aspergillus	*Aspergillus cristatus*	1
	Aspergillus	*Aspergillus carbonarius*	1
	Aspergillus	*Aspergillus bombycis*	1
	Endocarpon	*Endocarpon pusillum*	1
	Rhinocladiella	*Rhinocladiella mackenziei*	1
	Fonsecaea	*Fonsecaea multimorphosa*	1
	Exophiala	*Exophiala oligosperma*	1
	Exophiala	*Not specified*	1
	Capronia	*Capronia epimyces*	1
	Not specified	*Not specified*	1
	Saitoella	*Saitoella complicata*	1
	Pneumocystis	*Not specified*	1
	Gonapodya	*Gonapodya prolifera*	1

Abundance of fungal species identified in the metatranscriptome of sample derived from Spores 48h.

Library		Transcripts ≥5		Library		Transcripts <5	
	Genus	Species	Transcripts		Genus	Species	Transcripts
Spores_48h ai	*Pseudomonas*	*Not Specified*	4555	Spores_48h ai	*Streptococcus*	*Not Specified*	1
	Not Specified	*Not Specified*	2733		*Lactobacillus*	*Lactobacillus plantarum*	1
	Pseudomonas	*Pseudomonas parafulva*	2514		*Staphylococcus*	*Staphylococcus aureus*	1
	Stenotrophomonas	*Stenotrophomonas maltophilia*	651		*Staphylococcus*	*Not Specified*	1
	Pseudomonas	*Pseudomonas sp. NFR16*	614		*Paenibacillus*	*Paenibacillus sp. HW567*	1
	Pseudomonas	*Pseudomonas sp. Bc-h*	573		*Paenibacillus*	*Paenibacillus sp. FF9*	1
	Pseudomonas	*Pseudomonas putida*	572		*Paenibacillus*	*Paenibacillus odorifer*	1
	Enterobacter	*Enterobacter cancerogenus*	571		*Bacillus*	*Bacillus sp. JCM 19047*	1
	Pseudomonas	*Pseudomonas abietaniphila*	446		*Bacillus*	*Bacillus toyonensis*	1
	Not Specified	*Not Specified*	414		*Bacillus*	*Bacillus acidicola*	1
	Not Specified	*Type-E symbiont of Plautia stali*	402		*Not Specified*	*Not Specified*	1
	Not Specified	*Not Specified*	384		*Synechococcus*	*Not Specified*	1
	Not Specified	*Not Specified*	368		*Leptolyngbya*	*Leptolyngbya boryana*	1
	Acinetobacter	*Not Specified*	362		*Mastigocoleus*	*Mastigocoleus testarum*	1
	Stenotrophomonas	*Not Specified*	346		*Not Specified*	*Not Specified*	1
	Pseudomonas	*Pseudomonas graminis*	264		*Not Specified*	*Actinobacteria bacterium OV320*	1
	Pantoea	*Not Specified*	263		*Nonomuraea*	*Not Specified*	1
	Not Specified	*Not Specified*	235		*Streptomyces*	*Streptomyces zinciresistens*	1
	Pseudomonas	*Pseudomonas lutea*	189		*Streptomyces*	*Streptomyces sp. yr375*	1
	Pseudomonas	*Pseudomonas syringae*	158		*Streptomyces*	*Streptomyces sp. IMTB 2501*	1
	Pseudomonas	*Pseudomonas monteilii*	131		*Streptomyces*	*Streptomyces sp. H-KF8*	1
	Pseudomonas	*Pseudomonas cremoricolorata*	130		*Streptomyces*	*Streptomyces sp. DvalAA-14*	1
	Pseudomonas	*Not Specified*	126		*Streptomyces*	*Streptomyces rubellomurinus*	1
	Escherichia	*Escherichia coli*	123		*Streptomyces*	*Streptomyces pristinaespiralis*	1
	Pantoea	*Pantoea rodasii*	115		*Streptomyces*	*Not Specified*	1
	Pantoea	*Pantoea dispersa*	93		*Saccharomonospora*	*Saccharomonospora glauca*	1
	Not Specified	*Not Specified*	92		*Actinokineospora*	*Actinokineospora enzanensis*	1
	Pseudomonas	*Not Specified*	90		*Actinoplanes*	*Actinoplanes philippinensis*	1
	Pseudomonas	*Pseudomonas entomophila*	90		*Kocuria*	*Kocuria kristinae*	1
	Pseudomonas	*Pseudomonas fluorescens*	88		*Pseudoclavibacter*	*Pseudoclavibacter helvolus*	1
	Klebsiella	*Not Specified*	75		*Not Specified*	*Not Specified*	1

	Pseudomonas	*Pseudomonas sp. 250J*	67
	Pseudomonas	*Pseudomonas sp. NFACC02*	63
Spores_48h ai	*Pantoea*	*Pantoea rwandensis*	57
	Pantoea	*Pantoea ananatis*	56
	Pseudomonas	*Pseudomonas fuscovaginae*	52
	Pseudomonas	*Pseudomonas sp. NBRC 111131*	52
	Pseudomonas	*Pseudomonas plecoglossicida*	52
	Pantoea	*Pantoea sp. BL1*	51
	Pseudomonas	*Pseudomonas aeruginosa*	49
	Pseudomonas	*Pseudomonas mosselii*	48
	Pseudomonas	*Pseudomonas cichorii*	47
	Not Specified	*Not Specified*	46
	Pantoea	*Pantoea sp. SM3*	45
	Acinetobacter	*Acinetobacter baumannii*	45
	Acinetobacter	*Acinetobacter pittii*	43
	Klebsiella	*Klebsiella pneumoniae*	40
	Pseudomonas	*Pseudomonas sp. StFLB209*	36
	Acinetobacter	*Not Specified*	35
	Stenotrophomonas	*Stenotrophomonas rhizophila*	33
	Pseudomonas	*Pseudomonas chlororaphis*	32
	Pseudomonas	*Pseudomonas sp. GM84*	31
	Pseudomonas	*Pseudomonas sp. CB1*	31
	Pseudomonas	*Pseudomonas sp. NBRC 111124*	29
	Pseudomonas	*Pseudomonas sp. NBRC 111130*	27
	Pseudomonas	*Pseudomonas sp. CCOS 191*	27
	Pseudomonas	*Pseudomonas sp. Leaf127*	25
	Pseudomonas	*Pseudomonas sp. 10-1B*	25
	Not Specified	*gamma proteobacterium L18*	24
	Pseudomonas	*Pseudomonas batumici*	24
Spores_48h ai	*Xanthomonas*	*Xanthomonas citri*	20
	Salmonella	*Salmonella enterica*	20
	Williamsia	*Williamsia herbipolensis*	1
	Rhodococcus	*Rhodococcus sp. MTM3W5.2*	1
Spores_48h ai	*Rhodococcus*	*Rhodococcus rhodochrous*	1
	Nocardia	*Nocardia terpenica*	1
	Nocardia	*Nocardia jiangxiensis*	1
	Corynebacterium	*Corynebacterium efficiens*	1
	Catenulispora	*Catenulispora acidiphila*	1
	Actinopolyspora	*Actinopolyspora alba*	1
	Varibaculum	*Varibaculum cambriense*	1
	Not Specified	*Not Specified*	1
	Leptospira	*Leptospira sp. B5-022*	1
	Campylobacter	*Campylobacter jejuni*	1
	Not Specified	*uncultured Desulfobacterium sp.*	1
	Not Specified	*Gammaproteobacteria bacterium MFB021*	1
	Thiolapillus	*Thiolapillus brandeum*	1
	Not Specified	*Xanthomonadales bacterium RIFOXYA1_FULL_68_6*	1
	Xylella	*Xylella fastidiosa*	1
	Xanthomonas	*Xanthomonas sacchari*	1
	Xanthomonas	*Xanthomonas fuscans*	1
	Xanthomonas	*Xanthomonas cannabis*	1
	Xanthomonas	*Xanthomonas axonopodis*	1
	Stenotrophomonas	*Stenotrophomonas sp. RIT309*	1
	Stenotrophomonas	*Stenotrophomonas panacihumi*	1
	Stenotrophomonas	*Stenotrophomonas acidaminiphila*	1
	Pseudoxanthomonas	*Pseudoxanthomonas suwonensis*	1
	Pseudoxanthomonas	*Pseudoxanthomonas sp. Root65*	1
	Pseudoxanthomonas	*Pseudoxanthomonas sp. Root630*	1
	Pseudoxanthomonas	*Pseudoxanthomonas sp. CF385*	1
	Pseudoxanthomonas	*Pseudoxanthomonas sp. CF125*	1
Spores_48h ai	*Pseudoxanthomonas*	*Pseudoxanthomonas mexicana*	1
	Lysobacter	*Lysobacter sp. A03*	1

	Pseudomonas	*Pseudomonas sp. M47T1*	19
	Pseudomonas	*Pseudomonas japonica*	19
	Pseudomonas	*Pseudomonas stutzeri*	18
	Pseudomonas	*Pseudomonas sp. Leaf58*	18
	Pseudomonas	*Pseudomonas sp. WCHP16*	17
	Pseudomonas	*Pseudomonas gingeri*	17
	Pseudomonas	*Pseudomonas amygdali*	17
	Pseudomonas	*Pseudomonas sp. GEV388*	16
	Pantoea	*Pantoea sp. At-9b*	16
	Pseudomonas	*Pseudomonas sp. NBRC 111119*	15
	Pseudomonas	*Pseudomonas sp. 5*	15
	Pseudomonas	*Pseudomonas vranovensis*	14
	Pseudomonas	*Pseudomonas marginalis*	14
	Stenotrophomonas	*Stenotrophomonas sp. SC-N050*	13
	Pseudomonas	*Pseudomonas xanthomarina*	13
	Pseudomonas	*Pseudomonas endophytica*	13
	Pseudomonas	*Pseudomonas alkylphenolica*	13
	Pseudomonas	*Pseudomonas alcaligenes*	13
	Pseudomonas	*Pseudomonas sp. VLB120*	12
Spores_48h ai	*Enterobacter*	*Not Specified*	12
	Pseudomonas	*Pseudomonas sp. LAIL14HWK12:I11*	11
	Pseudomonas	*Pseudomonas rhizosphaerae*	11
	Pantoea	*Pantoea wallisii*	11
	Pseudomonas	*Pseudomonas sp. KK4*	10
	Pseudomonas	*Pseudomonas sp. ABAC61*	10
	Pseudomonas	*Pseudomonas helleri*	10
	Pseudomonas	*Not Specified*	10
	Shigella	*Shigella sonnei*	9
	Pseudomonas	*Pseudomonas brassicacearum*	9
	Pseudomonas	*Pseudomonas resinovorans*	9
	Not Specified	*Type-D symbiont of Plautia stali*	9
	Pseudomonas	*Pseudomonas savastanoi*	9
	Not Specified	*uncultured bacterium*	8
	Pseudomonas	*Pseudomonas taiwanensis*	8
	Pseudomonas	*Pseudomonas sp. S9*	8

	Lysobacter	*Lysobacter antibioticus*	1
	Lysobacter	*Not Specified*	1
	Luteimonas	*Luteimonas abyssi*	1
	Rhodanobacter	*Rhodanobacter sp. OK091*	1
	Metallibacterium	*Metallibacterium scheffleri*	1
	Dyella	*Dyella jiangningensis*	1
	Dyella	*Not Specified*	1
	Vibrio	*Vibrio nigripulchritudo*	1
	Vibrio	*Vibrio cholerae*	1
	Vibrio	*Not Specified*	1
	Not Specified	*Not Specified*	1
	Not Specified	*Pseudomonadales bacterium RIFCSPLOWO2_12_59_9*	1
	Pseudomonas	*Pseudomonas yamanorum*	1
	Pseudomonas	*Pseudomonas versuta*	1
	Pseudomonas	*Pseudomonas taeanensis*	1
	Pseudomonas	*Not Specified*	1
	Pseudomonas	*Not Specified*	1
	Pseudomonas	*Not Specified*	1
	Pseudomonas	*Pseudomonas balearica*	1
Spores_48h ai	*Pseudomonas*	*Pseudomonas sp. W15Feb9B*	1
	Pseudomonas	*Pseudomonas sp. UW4*	1
	Pseudomonas	*Pseudomonas sp. TTU2014-096BSC*	1
	Pseudomonas	*Pseudomonas sp. TTU2014-066ASC*	1
	Pseudomonas	*Pseudomonas sp. TJI-51*	1
	Pseudomonas	*Pseudomonas sp. T*	1
	Pseudomonas	*Pseudomonas sp. RIT-PI-r*	1
	Pseudomonas	*Pseudomonas sp. R62*	1
	Pseudomonas	*Pseudomonas sp. R37(2017)*	1
	Pseudomonas	*Pseudomonas sp. PI1*	1
	Pseudomonas	*Pseudomonas sp. PA27(2017)*	1
	Pseudomonas	*Pseudomonas sp. PA1(2017)*	1
	Pseudomonas	*Pseudomonas sp. OV546*	1
	Pseudomonas	*Pseudomonas sp. NFR09*	1
	Pseudomonas	*Pseudomonas sp. NFPP28*	1
	Pseudomonas	*Pseudomonas sp. NFIX28*	1

	Pseudomonas	*Pseudomonas sp. RIT-PI-a*	8
	Pseudomonas	*Pseudomonas sp. NBRC 111139*	8
	Pseudomonas	*Pseudomonas sp. NBRC 111127*	8
	Pseudomonas	*Pseudomonas sp. HMSC08G10*	8
	Pseudomonas	*Pseudomonas kuykendallii*	8
	Pseudomonas	*Pseudomonas veronii*	8
	Pantoea	*Pantoea sp. RIT-PI-b*	8
Spores_48h ai	*Not Specified*	*Plautia stali symbiont*	8
	Streptococcus	*Streptococcus pneumoniae*	7
	Pseudomonas	*Pseudomonas viridiflava*	7
	Pseudomonas	*Not Specified*	7
	Pseudomonas	*Pseudomonas sp. URMO17WK12:I11*	7
	Pseudomonas	*Pseudomonas sp. PH1b*	7
	Pseudomonas	*Pseudomonas sp. Ant30-3*	7
	Pseudomonas	*Pseudomonas tolaasii*	7
	Pseudomonas	*Pseudomonas taetrolens*	7
	Halotalea	*Halotalea alkalilenta*	7
	Legionella	*Legionella lansingensis*	7
	Edwardsiella	*Edwardsiella piscicida*	7
	Pantoea	*Pantoea sp. AS-PWVM4*	7
	Erwinia	*Erwinia amylovora*	7
	Enterobacter	*Enterobacter mori*	7
	Enterobacter	*Enterobacter ludwigii*	7
	Stenotrophomonas	*Stenotrophomonas sp. SKA14*	6
	Stenotrophomonas	*Stenotrophomonas sp. BIIR7*	6
	Stenotrophomonas	*Stenotrophomonas chelatiphaga*	6
	Pseudomonas	*Pseudomonas umsongensis*	6
	Pseudomonas	*Pseudomonas thermotolerans*	6
	Pseudomonas	*Not Specified*	6
	Pseudomonas	*Pseudomonas sp. PA15(2017)*	6
	Pseudomonas	*Pseudomonas psychrophila*	6
	Pseudomonas	*Pseudomonas indica*	6
	Pseudomonas	*Pseudomonas rhodesiae*	6
Spores_48h ai	*Pseudomonas*	*Pseudomonas cedrina*	6
	Pseudomonas	*Pseudomonas azotifigens*	6
	Pseudomonas	*Pseudomonas sp. NFACC25*	1
	Pseudomonas	*Pseudomonas sp. NFACC19-2*	1
	Pseudomonas	*Pseudomonas sp. NFACC13-1*	1
	Pseudomonas	*Pseudomonas sp. NBRC 111142*	1
	Pseudomonas	*Pseudomonas sp. NBRC 111134*	1
	Pseudomonas	*Pseudomonas sp. NBRC 111129*	1
	Pseudomonas	*Pseudomonas sp. NBRC 111121*	1
Spores_48h ai	*Pseudomonas*	*Pseudomonas sp. NBRC 111118*	1
	Pseudomonas	*Pseudomonas sp. NBRC 111117*	1
	Pseudomonas	*Pseudomonas sp. MF6394*	1
	Pseudomonas	*Pseudomonas sp. Leaf434*	1
	Pseudomonas	*Pseudomonas sp. Leaf129*	1
	Pseudomonas	*Pseudomonas sp. LPH1*	1
	Pseudomonas	*Pseudomonas sp. JY-Q*	1
	Pseudomonas	*Pseudomonas sp. In5*	1
	Pseudomonas	*Pseudomonas sp. ICMP 19500*	1
	Pseudomonas	*Pseudomonas sp. GM74*	1
	Pseudomonas	*Pseudomonas sp. GM55*	1
	Pseudomonas	*Pseudomonas sp. GM50*	1
	Pseudomonas	*Pseudomonas sp. GM49*	1
	Pseudomonas	*Pseudomonas sp. GM48*	1
	Pseudomonas	*Pseudomonas sp. GM102*	1
	Pseudomonas	*Pseudomonas sp. ENNP23*	1
	Pseudomonas	*Pseudomonas sp. EGD-AKN5*	1
	Pseudomonas	*Pseudomonas sp. DSM 29164*	1
	Pseudomonas	*Pseudomonas sp. DR 5-09*	1
	Pseudomonas	*Pseudomonas sp. CMR5c*	1
	Pseudomonas	*Pseudomonas sp. CMAA1215*	1
	Pseudomonas	*Pseudomonas sp. CC6-YY-74*	1
	Pseudomonas	*Pseudomonas sp. C9*	1
	Pseudomonas	*Pseudomonas sp. BTN1*	1
	Pseudomonas	*Pseudomonas sp. BRG-100*	1
	Pseudomonas	*Pseudomonas sp. B8(2017)*	1
Spores_48h ai	*Pseudomonas*	*Pseudomonas sp. B28(2017)*	1
	Pseudomonas	*Pseudomonas sp. B14(2017)*	1

	Pseudomonas	*Pseudomonas argentinensis*	6
	Pseudomonas	*Pseudomonas straminea*	6
	Pantoea	*Pantoea sp. GM01*	6
	Klebsiella	*Klebsiella variicola*	6
	Enterobacter	*Not Specified*	6
	Xanthomonas	*Not Specified*	5
	Stenotrophomonas	*Stenotrophomonas sp. 92mfcol6.1*	5
	Stenotrophomonas	*Stenotrophomonas pavanii*	5
	Pseudomonas	*Pseudomonas caricapapayae*	5
	Pseudomonas	*Pseudomonas asturiensis*	5
	Pseudomonas	*Pseudomonas sp. NBRC 111133*	5
	Pseudomonas	*Pseudomonas sp. H2*	5
	Pseudomonas	*Pseudomonas sp. EpS/L25*	5
	Pseudomonas	*Pseudomonas sp. CF161*	5
	Pseudomonas	*Pseudomonas saudiphocaensis*	5
	Pseudomonas	*Pseudomonas prosekii*	5
	Pseudomonas	*Pseudomonas panipatensis*	5
	Pseudomonas	*Pseudomonas mucidolens*	5
	Pseudomonas	*Pseudomonas gessardii*	5
	Pseudomonas	*Pseudomonas costantinii*	5
	Pseudomonas	*Pseudomonas fragi*	5
	Pseudomonas	*Pseudomonas agarici*	5
	Pseudomonas	*Pseudomonas nitroreducens*	5
Spores_48h ai	*Pantoea*	*Pantoea sp. A4*	5
	Not Specified	*Not Specified*	5
	Acinetobacter	*Acinetobacter calcoaceticus*	5
	Erwinia	*Erwinia billingiae*	5
	Pantoea	*Pantoea agglomerans*	5
	Enterobacter	*Enterobacter cloacae*	5
**Library**		**Transcripts <5**	
	**Genus**	**Species**	**Transcripts**
	Mycobacterium	*Mycobacterium abscessus*	4
	Vibrio	*Vibrio campbellii*	4
	Pseudomonas	*Pseudomonas cannabina*	4
	Pseudomonas	*Pseudomonas sp. p21*	4

	Pseudomonas	*Pseudomonas sp. B11(2017)*	1
	Pseudomonas	*Pseudomonas sp. AP19*	1
	Pseudomonas	*Pseudomonas sp. 58 R 3*	1
	Pseudomonas	*Pseudomonas sp. 313*	1
	Pseudomonas	*Pseudomonas sp. 31 R 17*	1
	Pseudomonas	*Pseudomonas sp. 2_1_26*	1
	Pseudomonas	*Pseudomonas sp. 28 E 9*	1
	Pseudomonas	*Pseudomonas sp. 25 E 4*	1
	Pseudomonas	*Pseudomonas saudimassiliensis*	1
	Pseudomonas	*Pseudomonas punonensis*	1
	Pseudomonas	*Pseudomonas denitrificans*	1
	Pseudomonas	*Pseudomonas moraviensis*	1
	Pseudomonas	*Pseudomonas moorei*	1
	Pseudomonas	*Pseudomonas knackmussii*	1
	Pseudomonas	*Pseudomonas guineae*	1
	Pseudomonas	*Pseudomonas grimontii*	1
	Pseudomonas	*Pseudomonas granadensis*	1
	Pseudomonas	*Pseudomonas formosensis*	1
	Pseudomonas	*Pseudomonas reptilivorous*	1
	Pseudomonas	*Pseudomonas orientalis*	1
	Pseudomonas	*Pseudomonas mandelii*	1
	Pseudomonas	*Pseudomonas libanensis*	1
	Pseudomonas	*Pseudomonas extremorientalis*	1
Spores_48h ai	*Pseudomonas*	*Pseudomonas azotoformans*	1
	Pseudomonas	*Pseudomonas cerasi*	1
	Pseudomonas	*Pseudomonas arsenicoxydans*	1
	Pseudomonas	*Pseudomonas oleovorans*	1
	Pseudomonas	*Pseudomonas flavescens*	1
	Pseudomonas	*Pseudomonas anguilliseptica*	1
	Psychrobacter	*Psychrobacter sp. CIP 110854*	1
	Perlucidibaca	*Perlucidibaca sp. BK296*	1
	Acinetobacter	*Acinetobacter ursingii*	1
	Acinetobacter	*Acinetobacter towneri*	1
	Acinetobacter	*Acinetobacter radioresistens*	1
	Acinetobacter	*Acinetobacter nectaris*	1

	Pseudomonas	*Pseudomonas sp. URIL14HWK12:I4*	4
	Pseudomonas	*Pseudomonas sp. UC 17F4*	4
	Pseudomonas	*Pseudomonas sp. P1.31*	4
	Pseudomonas	*Pseudomonas sp. NBRC 111136*	4
	Pseudomonas	*Pseudomonas sp. NBRC 111132*	4
	Pseudomonas	*Pseudomonas sp. ML96*	4
Spores_48h ai	*Pseudomonas*	*Pseudomonas sp. Leaf48*	4
	Pseudomonas	*Pseudomonas sp. LAB-08*	4
	Pseudomonas	*Pseudomonas sp. GM30*	4
	Pseudomonas	*Pseudomonas sp. DRA525*	4
	Pseudomonas	*Pseudomonas sp. CFII64*	4
	Pseudomonas	*Pseudomonas sp. BMS12*	4
	Pseudomonas	*Pseudomonas sp. ABFPK*	4
	Pseudomonas	*Pseudomonas soli*	4
	Pseudomonas	*Pseudomonas oryzihabitans*	4
	Pseudomonas	*Pseudomonas oryzae*	4
	Pseudomonas	*Pseudomonas hussainii*	4
	Pseudomonas	*Pseudomonas protegens*	4
	Pseudomonas	*Pseudomonas mediterranea*	4
	Pseudomonas	*Pseudomonas benzenivorans*	4
	Pseudomonas	*Pseudomonas pseudoalcaligenes*	4
	Pseudomonas	*Pseudomonas mendocina*	4
	Pseudomonas	*Pseudomonas citronellolis*	4
	Azotobacter	*Azotobacter beijerinckii*	4
	Haemophilus	*Not Specified*	4
	Serratia	*Serratia marcescens*	4
	Pantoea	*Pantoea sp. AS1*	4
	Pantoea	*Pantoea cypripedii*	4
	Curvibacter	*Curvibacter putative symbiont of Hydra magnipapillata*	4
Spores_48h ai	*Not Specified*	*Not Specified*	4
	Xanthomonas	*Xanthomonas arboricola*	4
	Cutibacterium	*Cutibacterium acnes*	4
	Staphylococcus	*Staphylococcus hominis*	3

	Acinetobacter	*Acinetobacter lwoffii*	1
	Acinetobacter	*Acinetobacter sp. 72431*	1
	Acinetobacter	*Acinetobacter sp. 25977_3*	1
	Acinetobacter	*Acinetobacter beijerinckii*	1
	Not Specified	*Not Specified*	1
	Mannheimia	*Mannheimia haemolytica*	1
Spores_48h ai	*Gilliamella*	*Gilliamella apicola*	1
	Marinobacterium	*Marinobacterium sp. ST58-10*	1
	Balneatrix	*Balneatrix alpica*	1
	Kushneria	*Kushneria avicenniae*	1
	Halomonas	*Halomonas sp. G11*	1
	Halomonas	*Halomonas sp. ALS9*	1
	Halomonas	*Halomonas sp. 'Soap Lake #7'*	1
	Halomonas	*Halomonas arcis*	1
	Nevskia	*Nevskia soli*	1
	Legionella	*Legionella shakespearei*	1
	Legionella	*Legionella oakridgensis*	1
	Yersinia	*Yersinia ruckeri*	1
	Serratia	*Serratia sp. ATCC 39006*	1
	Serratia	*Serratia sp. 14-2641*	1
	Serratia	*Serratia plymuthica*	1
	Serratia	*Serratia odorifera*	1
	Rahnella	*Rahnella sp. J11-6*	1
	Rahnella	*Rahnella aquatilis*	1
	Nissabacter	*Nissabacter archeti*	1
	Sodalis	*Sodalis-like endosymbiont of Proechinophthirus fluctus*	1
	Sodalis	*Sodalis praecaptivus*	1
	Sodalis	*Candidatus Sodalis sp. SoCistrobi*	1
	Pectobacterium	*Pectobacterium carotovorum*	1
Spores_48h ai	*Pectobacterium*	*Not Specified*	1
	Dickeya	*Dickeya sp. NCPPB 3274*	1
	Brenneria	*Brenneria goodwinii*	1
	Not Specified	*Not Specified*	1

	Mycobacterium	*Mycobacterium tuberculosis*	3
	Xanthomonas	*Xanthomonas hyacinthi*	3
	Stenotrophomonas	*Stenotrophomonas sp. TD3*	3
	Stenotrophomonas	*Stenotrophomonas humi*	3
	Stenotrophomonas	*Stenotrophomonas ginsengisoli*	3
	Pseudoxanthomonas	*Pseudoxanthomonas sp. GM95*	3
	Not Specified	*Not Specified*	3
	Not Specified	*Not Specified*	3
	Pseudomonas	*Pseudomonas xinjiangensis*	3
	Pseudomonas	*Pseudomonas vancouverensis*	3
	Pseudomonas	*Not Specified*	3
	Pseudomonas	*Pseudomonas sp. URIL14HWK12:I5*	3
	Pseudomonas	*Pseudomonas sp. URHB0015*	3
	Pseudomonas	*Pseudomonas sp. TTU2014-080ASC*	3
	Pseudomonas	*Pseudomonas sp. Root401*	3
	Pseudomonas	*Pseudomonas sp. RIT-PI-q*	3
	Pseudomonas	*Pseudomonas sp. Pf153*	3
	Pseudomonas	*Pseudomonas sp. PAMC 25886*	3
	Pseudomonas	*Pseudomonas sp. Os17*	3
	Pseudomonas	*Pseudomonas sp. LFM046*	3
	Pseudomonas	*Pseudomonas sp. H1h*	3
	Pseudomonas	*Pseudomonas sp. GM67*	3
	Pseudomonas	*Pseudomonas sp. GM18*	3
Spores_48h ai	*Pseudomonas*	*Pseudomonas sp. FeS53a*	3
	Pseudomonas	*Pseudomonas sp. ES3-33*	3
	Pseudomonas	*Pseudomonas sp. B10*	3
	Pseudomonas	*Pseudomonas sp. A3(2016)*	3
	Pseudomonas	*Pseudomonas simiae*	3
	Pseudomonas	*Pseudomonas saponiphila*	3
	Pseudomonas	*Pseudomonas psychrotolerans*	3
	Pseudomonas	*Pseudomonas palleroniana*	3
	Pseudomonas	*Pseudomonas otitidis*	3
	Pseudomonas	*Pseudomonas frederiksbergensis*	3
	Pseudomonas	*Pseudomonas donghuensis*	3
	Xenorhabdus	*Xenorhabdus doucetiae*	1
	Providencia	*Candidatus Providencia siddallii*	1
	Photorhabdus	*Photorhabdus luminescens*	1
	Photorhabdus	*Not Specified*	1
	Morganella	*Morganella morganii*	1
	Pantoea	*Pantoea sp. YR343*	1
	Pantoea	*Pantoea sp. PSNIH2*	1
	Pantoea	*Pantoea anthophila*	1
	Pantoea	*Pantoea alhagi*	1
	Erwinia	*Erwinia toletana*	1
	Erwinia	*Erwinia tasmaniensis*	1
	Erwinia	*Erwinia sp. Leaf53*	1
	Erwinia	*Erwinia sp. ErVv1*	1
	Erwinia	*Erwinia sp. 9145*	1
	Erwinia	*Erwinia mallotivora*	1
	Erwinia	*Erwinia iniecta*	1
	Erwinia	*Erwinia gerundensis*	1
	Not Specified	*Enterobacteriaceae bacterium strain FGI 57*	1
	Not Specified	*Not Specified*	1
	Trabulsiella	*Trabulsiella odontotermitis*	1
	Siccibacter	*Siccibacter colletis*	1
	Shimwellia	*Shimwellia blattae*	1
	Shigella	*Shigella sp. SF-2015*	1
Spores_48h ai	*Shigella*	*Not Specified*	1
	Salmonella	*Not Specified*	1
	Rosenbergiella	*Rosenbergiella nectarea*	1
	Raoultella	*Raoultella planticola*	1
	Pluralibacter	*Pluralibacter gergoviae*	1
	Mangrovibacter	*Mangrovibacter phragmitis*	1
	Lelliottia	*Lelliottia amnigena*	1
	Leclercia	*Leclercia adecarboxylata*	1
	Kosakonia	*Kosakonia radicincitans*	1
	Kosakonia	*Kosakonia oryziphila*	1
	Kluyvera	*Kluyvera intermedia*	1

	Pseudomonas	*Pseudomonas deceptionensis*	3		*Kluyvera*	*Kluyvera cryocrescens*	1
	Pseudomonas	*Pseudomonas asplenii*	3		*Klebsiella*	*Klebsiella sp. OBRC7*	1
	Pseudomonas	*Pseudomonas alcaliphila*	3		*Klebsiella*	*Klebsiella sp. MS 92-3*	1
	Pseudomonas	*Pseudomonas jinjuensis*	3		*Klebsiella*	*Klebsiella sp. 1_1_55*	1
	Pseudomonas	*Not Specified*	3		*Klebsiella*	*Klebsiella oxytoca*	1
	Legionella	*Legionella pneumophila*	3		*Klebsiella*	*Klebsiella michiganensis*	1
	Yersinia	*Yersinia enterocolitica*	3		*Franconibacter*	*Franconibacter pulveris*	1
Spores_48h ai	*Serratia*	*Serratia liquefaciens*	3	Spores_48h ai	*Franconibacter*	*Not Specified*	1
	Serratia	*Not Specified*	3		*Enterobacter*	*Enterobacter xiangfangensis*	1
	Pantoea	*Pantoea stewartii*	3		*Enterobacter*	*Enterobacter sp. BIDMC100*	1
	Pantoea	*Pantoea sp. OV426*	3		*Enterobacter*	*Enterobacter kobei*	1
	Pantoea	*Pantoea eucrina*	3		*Cronobacter*	*Cronobacter condimenti*	1
	Candidatus Hamiltonella	*Candidatus Hamiltonella defensa*	3		*Cronobacter*	*Not Specified*	1
	Klebsiella	*Klebsiella aerogenes*	3		*Citrobacter*	*Citrobacter koseri*	1
	Enterobacter	*Enterobacter hormaechei*	3		*Thiohalospira*	*Thiohalospira halophila*	1
	Thiocapsa	*Thiocapsa sp. KS1*	3		*Thiorhodococcus*	*Thiorhodococcus sp. AK35*	1
	Aeromonas	*Aeromonas hydrophila*	3		*Rheinheimera*	*Rheinheimera nanhaiensis*	1
	Paraburkholderia	*Candidatus Paraburkholderia schumannianae*	3		*Shewanella*	*Shewanella violacea*	1
	Burkholderia	*Burkholderia sp. KJ006*	3		*Marinobacter*	*Marinobacter sp. X15-166B*	1
	Burkholderia	*Not Specified*	3		*Marinobacter*	*Marinobacter hydrocarbonoclasticus*	1
	Not Specified	*Not Specified*	3		*Agarivorans*	*Agarivorans gilvus*	1
	Skermanella	*Skermanella stibiiresistens*	3		*Oceanisphaera*	*Oceanisphaera psychrotolerans*	1
	Donghicola	*Donghicola sp. KarMa*	3		*Aeromonas*	*Aeromonas sp. DNP9*	1
	Kaistia	*Kaistia granuli*	3		*Aeromonas*	*Aeromonas sp. ANNP30*	1
	Cronobacter	*Cronobacter sakazakii*	3		*Aeromonas*	*Not Specified*	1
	Clostridium	*Clostridium sp. W14A*	2		*Not Specified*	*beta proteobacterium CB*	1
	Paenibacillus	*Paenibacillus sp. IHB B 3415*	2		*Not Specified*	*Betaproteobacteria bacterium MOLA814*	1
	Mycobacterium	*Not Specified*	2		*Candidatus Accumulibacter*	*Candidatus Accumulibacter aalborgensis*	1
	Stenotrophomonas	*Stenotrophomonas sp. DDT-1*	2		*Not Specified*	*uncultured beta proteobacterium HF0010_04H24*	1
	Stenotrophomonas	*Stenotrophomonas koreensis*	2		*Vitreoscilla*	*Vitreoscilla stercoraria*	1
	Luteibacter	*Luteibacter sp. 9135*	2		*Neisseria*	*Not Specified*	1
	Not Specified	*Pseudomonadales bacterium RIFCSPLOWO2_02_FULL_63_210*	2		*Vogesella*	*Vogesella sp. EB*	1

Spores_48h ai	*Pseudomonas*	*Pseudomonas zeshuii*	2
	Pseudomonas	*Pseudomonas yangmingensis*	2
	Pseudomonas	*Pseudomonas thivervalensis*	2
	Pseudomonas	*Not Specified*	2
	Pseudomonas	*Pseudomonas coronafaciens*	2
	Pseudomonas	*Pseudomonas sp. Z003-0.4C(8344-21)*	2
	Pseudomonas	*Pseudomonas sp. VI4.1*	2
	Pseudomonas	*Pseudomonas sp. URIL14HWK12:I6*	2
	Pseudomonas	*Pseudomonas sp. TCU-HL1*	2
	Pseudomonas	*Pseudomonas sp. St29*	2
	Pseudomonas	*Pseudomonas sp. SHC52*	2
	Pseudomonas	*Pseudomonas sp. S1E40*	2
	Pseudomonas	*Pseudomonas sp. S-6-2*	2
	Pseudomonas	*Pseudomonas sp. Root562*	2
	Pseudomonas	*Pseudomonas sp. RIT357*	2
Spores_48h ai	*Pseudomonas*	*Pseudomonas sp. P482*	2
	Pseudomonas	*Pseudomonas sp. P1.8*	2
	Pseudomonas	*Pseudomonas sp. NBRC 111144*	2
	Pseudomonas	*Pseudomonas sp. MIACH*	2
	Pseudomonas	*Pseudomonas sp. MF4836*	2
	Pseudomonas	*Pseudomonas sp. Leaf15*	2
	Pseudomonas	*Pseudomonas sp. LAIL14HWK12:I7*	2
	Pseudomonas	*Pseudomonas sp. KG01*	2
	Pseudomonas	*Pseudomonas sp. GM33*	2
	Pseudomonas	*Pseudomonas sp. GM21*	2
	Pseudomonas	*Pseudomonas sp. GM17*	2
	Pseudomonas	*Pseudomonas sp. FGI182*	2
	Pseudomonas	*Pseudomonas sp. CHM02*	2
	Pseudomonas	*Pseudomonas sp. C5pp*	2
	Pseudomonas	*Pseudomonas sp. B5(2017)*	2
	Pseudomonas	*Pseudomonas sp. 22 E 5*	2
	Pseudomonas	*Pseudomonas sp. 2(2015)*	2
Spores_48h ai	*Pseudogulbenkiania*	*Pseudogulbenkiania sp. NH8B*	1
	Pseudogulbenkiania	*Pseudogulbenkiania sp. MAI-1*	1
	Microvirgula	*Microvirgula aerodenitrificans*	1
	Not Specified	*Not Specified*	1
	Not Specified	*Burkholderiales bacterium RIFCSPHIGHO2_01_FULL_63_240*	1
	Thiomonas	*Thiomonas sp. CB2*	1
	Methylibium	*Methylibium sp. T29-B*	1
	Parasutterella	*Parasutterella excrementihominis*	1
	Massilia	*Massilia sp. BSC265*	1
	Massilia	*Massilia sp. 9096*	1
	Janthinobacterium	*Janthinobacterium sp. HH01*	1
	Janthinobacterium	*Janthinobacterium sp. CG3*	1
	Janthinobacterium	*Janthinobacterium lividum*	1
	Herbaspirillum	*Herbaspirillum lusitanum*	1
	Herbaspirillum	*Herbaspirillum frisingense*	1
Spores_48h ai	*Herbaspirillum*	*Herbaspirillum chlorophenolicum*	1
	Duganella	*Duganella sp. CF458*	1
	Collimonas	*Collimonas fungivorans*	1
	Collimonas	*Collimonas arenae*	1
	Not Specified	*Comamonadaceae bacterium B1*	1
	Xenophilus	*Xenophilus azovorans*	1
	Variovorax	*Variovorax sp. Root318D1*	1
	Hylemonella	*Hylemonella gracilis*	1
	Comamonas	*Comamonas thiooxydans*	1
	Acidovorax	*Acidovorax sp. Root217*	1
	Acidovorax	*Not Specified*	1
	Not Specified	*bacterium endosymbiont of Mortierella elongata FMR23-6*	1
	Ralstonia	*Ralstonia sp. 25mfcol4.1*	1
	Paraburkholderia	*Paraburkholderia susongensis*	1
	Paraburkholderia	*Paraburkholderia piptadeniae*	1
	Paraburkholderia	*Paraburkholderia phymatum*	1
	Paraburkholderia	*Paraburkholderia mimosarum*	1

	Pseudomonas	*Pseudomonas sagittaria*	2
	Pseudomonas	*Pseudomonas fulva*	2
	Pseudomonas	*Pseudomonas pohangensis*	2
	Pseudomonas	*Pseudomonas mohnii*	2
	Pseudomonas	*Pseudomonas marincola*	2
	Pseudomonas	*Pseudomonas koreensis*	2
	Pseudomonas	*Pseudomonas guariconensis*	2
	Pseudomonas	*Pseudomonas guangdongensis*	2
	Pseudomonas	*Pseudomonas trivialis*	2
	Pseudomonas	*Pseudomonas migulae*	2
	Pseudomonas	*Pseudomonas corrugata*	2
	Pseudomonas	*Pseudomonas congelans*	2
	Pseudomonas	*Pseudomonas flexibilis*	2
	Pseudomonas	*Pseudomonas extremaustralis*	2
	Pseudomonas	*Pseudomonas capeferrum*	2
	Pseudomonas	*Pseudomonas borbori*	2
	Pseudomonas	*Pseudomonas antarctica*	2
	Oblitimonas	*Oblitimonas alkaliphila*	2
	Azotobacter	*Azotobacter vinelandii*	2
	Not Specified	*Not Specified*	2
Spores_48h ai	*Haemophilus*	*[Haemophilus] parasuis*	2
	Haemophilus	*Haemophilus influenzae*	2
	Halomonas	*Not Specified*	2
	Yersinia	*Yersinia pestis*	2
	Yersinia	*Not Specified*	2
	Rahnella	*Not Specified*	2
	Xenorhabdus	*Xenorhabdus bovienii*	2
	Proteus	*Not Specified*	2
	Photorhabdus	*Photorhabdus temperata*	2
	Edwardsiella	*Edwardsiella tarda*	2
	Pantoea	*Pantoea sp. IMH*	2
	Pantoea	*Pantoea septica*	2
	Pantoea	*Pantoea brenneri*	2
	Erwinia	*Erwinia typographi*	2
	Erwinia	*Erwinia tracheiphila*	2

	Paraburkholderia	*Paraburkholderia ferrariae*	1
	Paraburkholderia	*Paraburkholderia bryophila*	1
	Paraburkholderia	*Paraburkholderia aspalathi*	1
	Paraburkholderia	*Paraburkholderia andropogonis*	1
	Paraburkholderia	*Not Specified*	1
	Pandoraea	*Pandoraea oxalativorans*	1
	Lautropia	*Lautropia sp. SCN 69-89*	1
	Cupriavidus	*Not Specified*	1
	Burkholderia	*Burkholderia sp. JS23*	1
	Burkholderia	*Burkholderia gladioli*	1
	Burkholderia	*Burkholderia cenocepacia*	1
	Burkholderia	*Burkholderia arvi*	1
	Bordetella	*Bordetella genomosp. 13*	1
	Bordetella	*Bordetella ansorpii*	1
	Bordetella	*Not Specified*	1
	Advenella	*Advenella mimigardefordensis*	1
	Advenella	*Advenella kashmirensis*	1
	Achromobacter	*Not Specified*	1
	Not Specified	*Not Specified*	1
	Not Specified	*alpha proteobacterium AAP38*	1
Spores_48h ai	*Sphingopyxis*	*Not Specified*	1
	Sphingomonas	*Sphingomonas sp. PAMC 26605*	1
	Sphingomonas	*Sphingomonas sp. CCH5-D11*	1
	Sphingobium	*Sphingobium sp. YR657*	1
	Novosphingobium	*Novosphingobium sp. MD-1*	1
	Tistrella	*Tistrella mobilis*	1
	Thalassospira	*Thalassospira lucentensis*	1
	Magnetospirillum	*Magnetospirillum gryphiswaldense*	1
	Azospirillum	*Not Specified*	1
	Not Specified	*Acetobacteraceae bacterium AT-5844*	1
	Commensalibacter	*Commensalibacter intestini*	1
	Acidomonas	*Acidomonas methanolica*	1
	Acidiphilium	*Acidiphilium multivorum*	1
	Acetobacter	*Acetobacter nitrogenifigens*	1
	Acetobacter	*Not Specified*	1

	Erwinia	*Erwinia teleogrylli*	2
	Erwinia	*Erwinia sp. Ejp617*	2
	Erwinia	*Erwinia persicina*	2
	Erwinia	*Not Specified*	2
	Not Specified	*Type-C symbiont of Plautia stali*	2
	Not Specified	*Klebsiella variicola CAG:634*	2
	Escherichia	*Not Specified*	2
Spores_48h ai	*Enterobacter*	*Enterobacter sp. kpr-6*	2
	Enterobacter	*Enterobacter sp. FY-07*	2
	Citrobacter	*Citrobacter freundii*	2
	Cedecea	*Cedecea davisae*	2
	Thioalkalivibrio	*Thioalkalivibrio sp. ALgr1*	2
	Pseudogulbenkiania	*Not Specified*	2
	Limnohabitans	*Limnohabitans sp. Rim47*	2
	Paraburkholderia	*Paraburkholderia tropica*	2
	Paraburkholderia	*Paraburkholderia graminis*	2
	Burkholderia	*Burkholderia pseudomallei*	2
	Burkholderia	*Not Specified*	2
	Sphingobium	*Sphingobium yanoikuyae*	2
	Novosphingobium	*Not Specified*	2
	Rickettsia	*Rickettsia bellii*	2
	Roseobacter	*Roseobacter sp. AzwK-3b*	2
	Agrobacterium	*Not Specified*	2
	Methylobacterium	*Methylobacterium populi*	2
	Brucella	*Not Specified*	2
	Bradyrhizobium	*Bradyrhizobium sp. STM 3809*	2
	Bradyrhizobium	*Not Specified*	2
	Vibrio	*Vibrio parahaemolyticus*	2
	Acinetobacter	*Acinetobacter bouvetii*	2
	Erwinia	*Erwinia pyrifoliae*	2
	Shigella	*Shigella flexneri*	2
	Escherichia	*Escherichia vulneris*	2
Spores_48h ai	*Aliiglaciecola*	*Aliiglaciecola lipolytica*	2

	Thalassobacter	*Not Specified*	1
	Salinihabitans	*Salinihabitans flavidus*	1
	Roseobacter	*Roseobacter sp. GAI101*	1
	Roseibium	*Roseibium sp. TrichSKD4*	1
	Jannaschia	*Jannaschia aquimarina*	1
	Confluentimicrobium	*Confluentimicrobium sp. EMB200-NS6*	1
	Xanthobacter	*Xanthobacter sp. 126*	1
Spores_48h ai	*Rhizobium*	*Rhizobium sullae*	1
	Rhizobium	*Rhizobium sp. YS-1r*	1
	Rhizobium	*Rhizobium sp. NFR07*	1
	Rhizobium	*Rhizobium sp. Leaf371*	1
	Rhizobium	*Rhizobium lusitanum*	1
	Rhizobium	*Not Specified*	1
	Pararhizobium	*Pararhizobium polonicum*	1
	Neorhizobium	*Neorhizobium galegae*	1
	Agrobacterium	*Agrobacterium rhizogenes*	1
	Pseudaminobacter	*Pseudaminobacter salicylatoxidans*	1
	Pseudaminobacter	*Pseudaminobacter manganicus*	1
	Aliihoeflea	*Aliihoeflea sp. 2WW*	1
	Methylobacterium	*Methylobacterium mesophilicum*	1
	Methylobacterium	*Not Specified*	1
	Rhodoplanes	*Rhodoplanes sp. Z2-YC6860*	1
	Devosia	*Devosia sp. H5989*	1
	Ochrobactrum	*Ochrobactrum intermedium*	1
	Rhodopseudomonas	*Rhodopseudomonas palustris*	1
	Bradyrhizobium	*Bradyrhizobium sp. STM 3843*	1
	Methylocella	*Methylocella silvestris*	1
	Not Specified	*Not Specified*	1
	Caulobacter	*Caulobacter sp. K31*	1
	Opitutus	*Opitutus sp. GAS368*	1
	Chlamydia	*Chlamydia trachomatis*	1
	Chlamydia	*Chlamydia muridarum*	1
Spores_48h ai	*Not Specified*	*Nitrospirae bacterium RBG_19FT_COMBO_55_12*	1

	Rudanella	*Rudanella lutea*	1
	Bacteroides	*Bacteroides fragilis*	1
	Not Specified	*bacteria symbiont BFo1 of Frankliniella occidentalis*	1
	Not Specified	*uncultured bacterium 5H7*	1
	Not Specified	*Entomoplasmatales bacterium EntAcro1*	1
	Lachnoclostridium	*[Clostridium] asparagiforme*	1
	Clostridium	*Clostridium sp. DMHC 10*	1
	Nitrospira	*Candidatus Nitrospira nitrosa*	1
	Sphingobacterium	*Sphingobacterium sp. IITKGP-BTPF85*	1
	Mucilaginibacter	*Mucilaginibacter mallensis*	1
	Gillisia	*Gillisia sp. CAL575*	1
	Solirubrum	*Solirubrum puertoriconensis*	1
	Hydrogenobaculum	*Hydrogenobaculum sp. Y04AAS1*	1

Abundance of bacteria species identified in the metatranscriptome of sample derived from Spores 48h.

Library		Transcripts ≥5	
	Genus	Species	Transcripts
	Not specified	*Not specified*	24
	Not specified	*Not specified*	21
Mycelium	*Rhizoctonia*	*Rhizoctonia solani*	9
	Not specified	*Not specified*	8
	Pseudocercospora	*Pseudocercospora fijiensis*	6
	Puccinia	*Puccinia striiformis*	5

Library		Transcripts <5	
	Genus	Species	Transcripts
	Serpula	*Serpula lacrymans*	4
	Lentinula	*Lentinula edodes*	4
Mycelium	*Gymnopus*	*Gymnopus luxurians*	4
	Not specified	*Not specified*	4
	Scleroderma	*Scleroderma citrinum*	3
	Galerina	*Galerina marginata*	3
	Schizophyllum	*Schizophyllum commune*	3
	Not specified	*Not specified*	3
	Not specified	*Not specified*	3
	Jaapia	*Jaapia argillacea*	2
	Rhizopogon	*Rhizopogon vinicolor*	2
	Pleurotus	*Pleurotus ostreatus*	2
	Hypsizygus	*Hypsizygus marmoreus*	2
	Fibroporia	*Fibroporia radiculosa*	2
	Sanghuangporus	*Sanghuangporus baumii*	2
	Not specified	*Not specified*	2
	Ophiocordyceps	*Ophiocordyceps unilateralis*	2
	Zymoseptoria	*Zymoseptoria tritici*	2
	Trichoderma	*Trichoderma virens*	2
	Not specified	*Not specified*	2
	Acidomyces	*Acidomyces richmondensis*	2
	Bipolaris	*Bipolaris maydis*	1
	Not specified	*Not specified*	1

Library		Transcripts <5	
	Genus	Species	Transcripts
	Mixia	*Mixia osmundae*	1
	Tremella	*Tremella mesenterica*	1
Mycelium	*Cryptococcus*	*Cryptococcus amylolentus*	1
	Fibulorhizoctonia	*Fibulorhizoctonia sp. CBS 109695*	1
	Plicaturopsis	*Plicaturopsis crispa*	1
	Cylindrobasidium	*Cylindrobasidium torrendii*	1
	Not specified	*Not specified*	1
	Not specified	*Not specified*	1
	Hebeloma	*Hebeloma cylindrosporum*	1
	Amanita	*Amanita muscaria*	1
	Leucoagaricus	*Leucoagaricus sp. SymC.cos*	1
	Agaricus	*Agaricus bisporus*	1
	Serendipita	*Serendipita indica*	1
	Peniophora	*Peniophora sp. CONT*	1
	Pycnoporus	*Pycnoporus coccineus*	1
	Dichomitus	*Dichomitus squalens*	1
	Phlebiopsis	*Phlebiopsis gigantea*	1
	Neolentinus	*Neolentinus lepideus*	1
	Gloeophyllum	*Gloeophyllum trabeum*	1
	Exidia	*Exidia glandulosa*	1
	Hypoxylon	*Hypoxylon sp. EC38*	1
	Neurospora	*Neurospora tetrasperma*	1
Mycelium	*Fusarium*	*Fusarium pseudograminearum*	1
	Not specified	*Not specified*	1
	Colletotrichum	*Colletotrichum gloeosporioides*	1
	Oidiodendron	*Oidiodendron maius*	1
	Cenococcum	*Cenococcum geophilum*	1
	Sphaerulina	*Sphaerulina musiva*	1
	Talaromyces	*Talaromyces marneffei*	1
	Penicillium	*Penicillium freii*	1
	Saitoella	*Saitoella complicata*	1

	Ustilago	*Ustilago maydis*	1		*Not specified*	*Not specified*	1
	Moesziomyces	*Moesziomyces antarcticus*	1				

Abundance of fungal species identified in the metatranscriptome of sample derived from Mycelium.

Library	Transcripts ≥5		
	**Genus**	**Species**	**Transcripts**
	Escherichia	*Escherichia coli*	41
	Not Specified	*Not Specified*	11
	Xanthomonas	*Xanthomonas citri*	10
Mycelium	*Pseudomonas*	*Not Specified*	9
	Not Specified	*Not Specified*	9
	Not Specified	*Not Specified*	7
	Bordetella	*Bordetella pertussis*	7
	Haemophilus	*Not Specified*	5

Library	Transcripts <5		
	**Genus**	**Species**	**Transcripts**
	Sporocytophaga	*Sporocytophaga myxococcoides*	4
	Pantoea	*Pantoea ananatis*	4
	Pseudomonas	*Not Specified*	3
Mycelium	*Curvibacter*	*Curvibacter putative symbiont of Hydra magnipapillata*	3
	Komagataeibacter	*Komagataeibacter nataicola*	3
	Vibrio	*Vibrio parahaemolyticus*	3
	Burkholderia	*Burkholderia mallei*	3
	Salmonella	*Salmonella enterica*	3
	Streptococcus	*Not Specified*	2
	Not Specified	*uncultured gamma proteobacterium HF0010_05D02*	2
	Pseudomonas	*Pseudomonas syringae*	2
	Legionella	*Legionella feeleii*	2
	Enterobacter	*Enterobacter sp. MGH 16*	2
	Not Specified	*Not Specified*	2
	Thiomonas	*Thiomonas sp. CB2*	2
Mycelium	*Taylorella*	*Taylorella asinigenitalis*	2
	Magnetospirillum	*Magnetospirillum gryphiswaldense*	2
	Azospirillum	*Azospirillum brasilense*	2
	Roseibium	*Roseibium sp. TrichSKD4*	2
	Agrobacterium	*Agrobacterium genomosp. 2*	2
	Brucella	*Brucella suis*	2
	Brucella	*Brucella pinnipedialis*	2

Library	Transcripts <5		
	**Genus**	**Species**	**Transcripts**
	Streptococcus	*Streptococcus anginosus*	1
	Paenibacillus	*Paenibacillus sp. FF9*	1
	Paenibacillus	*Paenibacillus selenitireducens*	1
Mycelium	*Streptomyces*	*Streptomyces sp. Amel2xC10*	1
	Streptomyces	*Streptomyces ghanaensis*	1
	Cutibacterium	*Cutibacterium acnes*	1
	Rothia	*Rothia dentocariosa*	1
	Microbacterium	*Microbacterium sp. HM58-2*	1
	Rhodococcus	*Rhodococcus ruber*	1
	Mycobacterium	*Mycobacterium tuberculosis*	1
	Not Specified	*Not Specified*	1
	Not Specified	*Not Specified*	1
	Not Specified	*methanotrophic bacterial endosymbiont of Bathymodiolus sp.*	1
Mycelium	*Pseudomonas*	*Pseudomonas viridiflava*	1
	Pseudomonas	*Pseudomonas savastanoi*	1
	Pseudomonas	*Not Specified*	1
	Pseudomonas	*Pseudomonas lutea*	1
	Not Specified	*Not Specified*	1
	Halomonas	*Halomonas titanicae*	1
	Tatlockia	*Not Specified*	1
	Rahnella	*Not Specified*	1
	Pantoea	*Pantoea wallisii*	1
	Shigella	*Shigella sonnei*	1
	Shigella	*Not Specified*	1
	Enterobacter	*Enterobacter sp. EGD-HP1*	1
Mycelium	*Enterobacter*	*Enterobacter hormaechei*	1
	Cronobacter	*Cronobacter sakazakii*	1
	Neisseria	*Not Specified*	1
	Not Specified	*Not Specified*	1
	Paraburkholderia	*Candidatus Paraburkholderia schumannianae*	1
	Burkholderia	*Not Specified*	1
	Burkholderia	*Burkholderia sp. RPE67*	1

	Candidatus Pelagibacter	*Candidatus Pelagibacter sp. HTCC7211*	2		*Burkholderia*	*Burkholderia sp. MSHR3999*	1
	Hymenobacter	*Hymenobacter sp. CCM 8649*	2		*Not Specified*	*Not Specified*	1
	Acinetobacter	*Acinetobacter baumannii*	2		*Thalassospira*	*Thalassospira xiamenensis*	1
	Klebsiella	*Klebsiella pneumoniae*	2		*Rhizobium*	*Rhizobium sp. NT-26*	1
	Not Specified	*uncultured bacterium A1Q1_fos_4*	1		*Mesorhizobium*	*Mesorhizobium loti*	1
	Not Specified	*uncultured bacterium 5H7*	1		*Brucella*	*Not Specified*	1
	Not Specified	*uncultured bacterium*	1		*Not Specified*	*Not Specified*	1
	Streptococcus	*Streptococcus pneumoniae*	1		*Not Specified*	*SAR11 cluster bacterium PRT-SC02*	1

Supplementary Table 11. Abundance of bacteria species identified in the metatranscriptome of sample derived from Mycelium.
