# Supplementary material for: Characterization of the microbiota dynamics associated with Moniliophthora roreri, causal agent of cocoa frosty pod rot disease, reveals new viral species
Source: Front Microbiol. 2023 Feb 3;13:1053562. doi: 10.3389/fmicb.2022.1053562 (PMC9936985; doi:10.3389/fmicb.2022.1053562)
Supplement: Supplementary file 2 [file Data_Sheet_2.PDF]

Supplementary Material

Characterization of the microbiota dynamics associated with *Moniliophthora roreri*, causal agent of cocoa pod rot disease, reveals new viral species

Brayan Maudiel Diaz Reyes[1]; Paula Luize Camargos Fonseca[1,2]; Neander Marcel Heming[1]; Lucas Barbosa de Amorim Conceição[3]; Katiucia Ticila de Souza Nascimento[1]; Karina Peres Gramacho[4]; Enrique Arevalo-Gardini[5,6]; Carlos Priminho Pirovani[1]; Eric Roberto Guimarães Rocha Aguiar[1,*]

[1]Departamento de Ciências Biológicas, Universidade Estadual de Santa Cruz, Ilhéus, Bahia, CEP 45662900, Brazil; 1994bmaudiel@gmail.com; katyticila@gmail.com; pirovanicp@gmail.com; ericgdp@gmail.com;
[2]Departamento de Genética, Instituto de Ciências Biológicas, Universidade Federal de Minas Gerais, Belo Horizonte, Minas Gerais, CEP 30270-901, Brazil; camargos.paulaluize@gmail.com
[3]Instituto de Biologia, Universidade Federal da Bahia, Salvador, Bahia, CEP 40170-115, Brazil; lucasbarbosa1714@gmail.com
[4]Centro de Pesquisas do Cacau, Comissão Executivo do Plano da Lavoura Cacaueira, CEPEC/CEPLAC, Rodovia Ilhéus-Itabuna, Km 22, Ilhéus/Ba, Brasil. Cep: 45662-000.
[5]Instituto de Cultivos Tropicales, ICT, Tarapoto/Peru.
[6]Universidad Nacional Autónoma de Alto Amazonas - UNAAA, Yurimaguas, Perú.

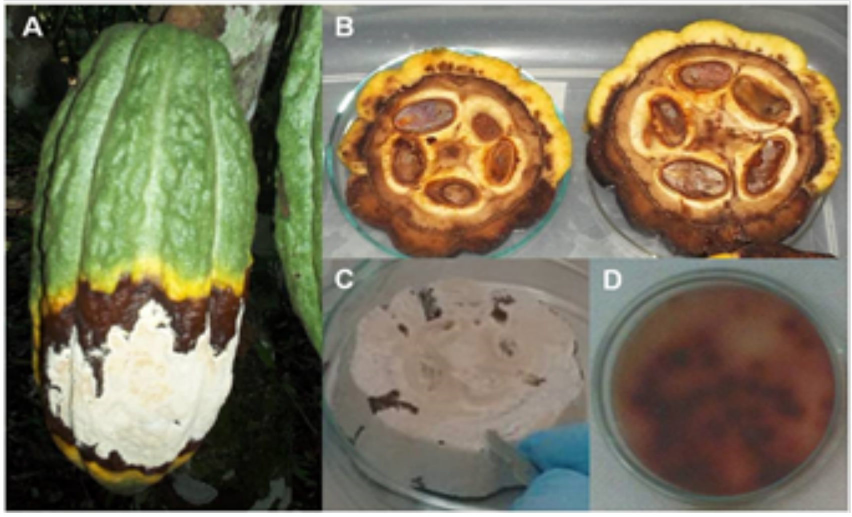

Supplementary Figure 1. Steps to obtain the spores and mycelium of *M. roreri* fungus in the ICT - Tarapoto / Peru. A. Fruit naturally infected with *M. roreri*; B. Slices in a humid chamber for 3 to 4 days at room temperature; C. Growth of the vegetative mycelium; D. Spore culture.

	Spores_0hai	Spores_8hai	Spores_ 16hai	Spores_48hai	Mycelium
**Raw**	25,840,512	17,590,721	13,700,034	16,678,051	12,384,924
**Reads after quality filter(phred> 30)**	25,619,553	17,423,068	13,542,853	16,545,502	12,315,054
**Reads not mapped to *M. roreri* genome**	9,578,356	13,394,360	6,683,356	14,404,718	5,917,329
**Assembled transcripts**	26,087	29,050	27,453	65,605	20,879
**N50**	1088	604	774	733	785
**Transcripts average length (nt)**	780	508	602	559	610
**Size longest contig (nt)**	10795	13909	10795	10795	10795

Supplementary Table 1. Overview of libraries processing per condition. hai: hours after inoculation.

Condition	Bacteria richness	Fungi richness
Spores_0hai	25	35
Spores_8hai	70	76
Spores_16hai	48	50
Spores_48hai	79	102
Mycelium	31	39

Supplementary Table 2. Fungi and bacterial richness per condition. hai: hours after inoculation.

Condition	beta.jtu	beta.jne	beta.jac	beta.ratio
Spores_0hai - Spores_8hai	0.276	0.440	0.716	0.385
Spores_8hai - Spores_16hai	0.316	0.191	0.506	0.625
Spores_16hai - Spores_48hai	0.316	0.241	0.557	0.567
Spores_48hai - Mycelium	0.121	0.521	0.642	0.188
Spores_0hai - Mycelium	0.387	0.099	0.486	0.796

Supplementary Table 3. Temporal changes in Beta-diversity of Bacterial species. jtu = turnover; jne = nestedness; jac = jackniffe; ratio = proportion (jtu/jac). hai: hours after inoculation.

**Condition**	**beta.jtu**	**beta.jne**	**beta.jac**	**beta.ratio**
Spores_0hai - Spores_8hai	0.372	0.306	0.679	0.548
Spores_8hai - Spores_16hai	0.387	0.181	0.568	0.681
Spores_16hai - Spores_48hai	0.387	0.280	0.667	0.580
Spores_48hai - Mycelium	0.340	0.378	0.718	0.474
Spores_0hai - Mycelium	0.409	0.049	0.458	0.893

Supplementary Table 4. Temporal changes in Beta-diversity of Fungi species. jtu = turnover; jne = nestedness; jac = jackniffe; ratio = proportion (jtu/jac). hai: hours after inoculation.

Libraries	Query ID	Bit score	Subject title
Spores 0hai	TRINITY_DN11667_c0_g1_i1	806	QHD64827.1 RdRp [Erysiphe necator associated narnavirus 4]
	TRINITY_DN11667_c0_g1_i1	398	QJV68034.1 putative RNA-dependent RNA polymerase [Magnaporthe oryzae narnavirus 1]
	TRINITY_DN11667_c0_g1_i1	398	BCH36656.1 RNA-dependent RNA polymerase [Magnaporthe oryzae narnavirus 1]
	TRINITY_DN11667_c0_g1_i1	372	QIR30299.1 RNA-dependent RNA polymerase [Plasmopara viticola lesion associated narnavirus 20]
	TRINITY_DN11453_c0_g1_i1	671	QED42934.1 putative RdRp [Monilinia narnavirus H]
	TRINITY_DN11453_c0_g1_i1	477	QIR30307.1 RNA-dependent RNA polymerase [Plasmopara viticola lesion associated narnavirus 28]
	TRINITY_DN11453_c0_g1_i1	193	QIR30306.1 RNA-dependent RNA polymerase [Plasmopara viticola lesion associated narnavirus 27]
	TRINITY_DN4781_c0_g1_i1	202	BCH36655.1 hypothetical protein [Magnaporthe oryzae narnavirus 1]
	TRINITY_DN4781_c0_g2_i1	133	BCH36655.1 hypothetical protein [Magnaporthe oryzae narnavirus 1]
Spores 8hai	TRINITY_DN5828_c0_g1_i1	84.3	BCH36655.1 hypothetical protein [Magnaporthe oryzae narnavirus 1]
	TRINITY_DN15040_c0_g1_i1	806	QHD64827.1 RdRp [Erysiphe necator associated narnavirus 4]
	TRINITY_DN15040_c0_g1_i1	398	QJV68034.1 putative RNA-dependent RNA polymerase [Magnaporthe oryzae narnavirus 1]
	TRINITY_DN15040_c0_g1_i1	397	BCH36656.1 RNA-dependent RNA polymerase [Magnaporthe oryzae narnavirus 1]
	TRINITY_DN15040_c0_g1_i1	372	QIR30299.1 RNA-dependent RNA polymerase [Plasmopara viticola lesion associated narnavirus 20]
	TRINITY_DN8478_c0_g1_i1	201	BCH36655.1 hypothetical protein [Magnaporthe oryzae narnavirus 1]
Spores 16hai	TRINITY_DN5928_c0_g1_i1	807	QHD64827.1 RdRp [Erysiphe necator associated narnavirus 4]
	TRINITY_DN5928_c0_g1_i1	398	QJV68034.1 putative RNA-dependent RNA polymerase [Magnaporthe oryzae narnavirus 1]
	TRINITY_DN5928_c0_g1_i1	398	BCH36656.1 RNA-dependent RNA polymerase [Magnaporthe oryzae narnavirus 1]
	TRINITY_DN5928_c0_g1_i1	372	QIR30299.1 RNA-dependent RNA polymerase [Plasmopara viticola lesion associated narnavirus 20]
	TRINITY_DN18983_c0_g1_i1	671	QED42934.1 putative RdRp [Monilinia narnavirus H]
	TRINITY_DN18983_c0_g1_i1	477	QIR30307.1 RNA-dependent RNA polymerase [Plasmopara viticola lesion associated narnavirus 28]
	TRINITY_DN18983_c0_g1_i1	193	QIR30306.1 RNA-dependent RNA polymerase [Plasmopara viticola lesion associated narnavirus 27]
	TRINITY_DN19217_c0_g1_i1	79.3	BCH36655.1 hypothetical protein [Magnaporthe oryzae narnavirus 1]
	TRINITY_DN2324_c0_g1_i1	236	BCH36655.1 hypothetical protein [Magnaporthe oryzae narnavirus 1]
Spores 48hai	TRINITY_DN3790_c0_g1_i1	550	QHD64827.1 RdRp [Erysiphe necator associated narnavirus 4]
	TRINITY_DN3790_c0_g1_i1	322	QIR30299.1 RNA-dependent RNA polymerase [Plasmopara viticola lesion associated narnavirus 20]
	TRINITY_DN3790_c0_g1_i1	313	QJV68034.1 putative RNA-dependent RNA polymerase [Magnaporthe oryzae narnavirus 1]
	TRINITY_DN3790_c0_g1_i1	313	BCH36656.1 RNA-dependent RNA polymerase [Magnaporthe oryzae narnavirus 1]
	TRINITY_DN40268_c0_g1_i1	204	QHD64827.1 RdRp [Erysiphe necator associated narnavirus 4]
	TRINITY_DN40729_c0_g1_i1	76.3	QHD64827.1 RdRp [Erysiphe necator associated narnavirus 4]

Supplementary Table 5. **Sequence similarity results regarding virus sequences.** Top results of Diamond blastx search against NR database. hai: hours after inoculation.

Description	Scientific Name	Max Score	Total Score	Query Cover	E value	Per. ident	Acc. Len	Accession
RdRp [Erysiphe necator associated narnavirus 4]	Erysiphe necator associated narnavirus 4	810	810	92%	0	52.7	775	QHD64827.1
RNA-dependent RNA polymerase [Magnaporthe oryzae narnavirus 1]	Magnaporthe oryzae narnavirus 1	402	402	90%	4.00E-124	35.86	786	QVU39992.1
putative RNA-dependent RNA polymerase [Magnaporthe oryzae narnavirus 1]	Magnaporthe oryzae narnavirus 1	397	397	90%	3.00E-122	35.9	783	QJV68034.1
RNA-dependent RNA polymerase [Magnaporthe oryzae narnavirus 1]	Magnaporthe oryzae narnavirus 1	396	396	90%	5.00E-122	35.91	783	BCH36656.1
putative RNA dependent RNA polymerase [Oidiodendron maius splipalmivirus 1]	Oidiodendron maius splipalmivirus 1	391	391	88%	4.00E-120	35.11	768	QNN89179.1
RNA-dependent RNA polymerase [Erysiphe necator associated narnavirus 46]	Erysiphe necator associated narnavirus 46	372	372	91%	3.00E-113	33.33	760	QJT93778.1
RNA-dependent RNA polymerase [Plasmopara viticola lesion associated narnavirus 20]	Plasmopara viticola lesion associated narnavirus 20	371	371	91%	2.00E-112	33.2	760	QIR30299.1
RNA-dependent RNA polymerase [Erysiphe necator associated narnavirus 35]	Erysiphe necator associated narnavirus 35	83.2	83.2	70%	4.00E-12	21.64	776	QJT93767.1
RNA-dependent RNA polymerase [Botrytis cinerea binarnavirus 2]	Botrytis cinerea binarnavirus 2	82.8	82.8	64%	5.00E-12	23.23	813	QJT73725.1
RNA-dependent RNA polymerase [Erysiphe necator associated narnavirus 13]	Erysiphe necator associated narnavirus 13	75.9	75.9	43%	8.00E-10	23.66	810	QJT93745.1
RNA-dependent RNA polymerase [Aspergillus fumigatus narnavirus 2]	Aspergillus fumigatus narnavirus 2	73.9	73.9	70%	2.00E-09	21.72	626	BCH36633.1
RNA-dependent RNA polymerase [Aspergillus fumigatus narnavirus 2]	Aspergillus fumigatus narnavirus 2	72	72	61%	1.00E-08	21.81	626	AXE72934.1
RNA-dependent RNA polymerase [Botrytis cinerea binarnavirus 1]	Botrytis cinerea binarnavirus 1	72	72	45%	1.00E-08	23.6	825	QJT73724.1
RNA-dependent RNA polymerase [Neofusicoccum parvum narnavirus 2]	Neofusicoccum parvum narnavirus 2	70.9	70.9	60%	2.00E-08	22.95	611	QDB74995.1
RNA-dependent RNA polymerase [Aspergillus fumigatus narnavirus 2]	Aspergillus fumigatus narnavirus 2	70.1	70.1	61%	4.00E-08	21.79	626	BCH36627.1
RNA-dependent RNA polymerase [Aspergillus fumigatus narnavirus 2]	Aspergillus fumigatus narnavirus 2	69.7	69.7	61%	5.00E-08	21.79	626	BCH36622.1
RNA-dependent RNA polymerase [Plasmopara viticola lesion associated narnavirus 6]	Plasmopara viticola lesion associated narnavirus 6	67.8	67.8	39%	2.00E-07	22.77	621	QIR30285.1

RNA-dependent RNA polymerase [Plasmopara viticola lesion associated narnavirus 19]	Plasmopara viticola lesion associated narnavirus 19	67.4	67.4	45%	3.00E-07	23.65	807	QIR30298.1
RNA-dependent RNA polymerase [Erysiphe necator associated narnavirus 12]	Erysiphe necator associated narnavirus 12	65.9	65.9	45%	9.00E-07	22.84	825	QJT93744.1
RNA-dependent RNA polymerase [Cladosporium tenuissimum narnavirus 1]	Cladosporium tenuissimum narnavirus 1	64.7	64.7	68%	2.00E-06	21.57	754	QDB74996.1
RNA-dependent RNA polymerase [Erysiphe necator associated narnavirus 49]	Erysiphe necator associated narnavirus 49	64.3	64.3	61%	3.00E-06	20.57	697	QJT93781.1
RNA-dependent RNA polymerase [Erysiphe necator associated narnavirus 50]	Erysiphe necator associated narnavirus 50	63.5	63.5	45%	4.00E-06	23.94	824	QJT93782.1
RNA-dependent RNA polymerase [Plasmopara viticola lesion associated narnavirus 1]	Plasmopara viticola lesion associated narnavirus 1	63.5	63.5	45%	5.00E-06	23.94	824	QIR30280.1
RNA-dependent RNA polymerase [Aspergillus fumigatus narnavirus 1]	Aspergillus fumigatus narnavirus 1	62	62	45%	1.00E-05	20.91	618	AXE72933.1
RNA-dependent RNA polymerase [Erysiphe necator associated narnavirus 24]	Erysiphe necator associated narnavirus 24	58.5	58.5	68%	2.00E-04	22.06	751	QJT93756.1
RNA-dependent RNA polymerase [Plasmopara viticola lesion associated narnavirus 5]	Plasmopara viticola lesion associated narnavirus 5	57.8	57.8	36%	3.00E-04	22.59	650	QIR30284.1
RNA-dependent RNA polymerase [Erysiphe necator associated narnavirus 30]	Erysiphe necator associated narnavirus 30	55.1	55.1	59%	0.002	22.13	641	QJT93762.1
RdRp [Erysiphe necator associated narnavirus 5]	Erysiphe necator associated narnavirus 5	53.9	53.9	61%	0.004	22.05	640	QHD64828.1

Supplementary Table 6. On line Blastx results for Contig 1.

Description	Scientific Name	Max Score	Total Score	Query Cover	E value	Per. ident	Acc. Len	Accession
hypothetical protein [Magnaporthe oryzae narnavirus 1]	Magnaporthe oryzae narnavirus 1	322	322	85%	3.00E-94	30.56	795	BCH36655.1
putative RNA dependent RNA polymerase [Oidiodendron maius splipalmivirus 1]	Oidiodendron maius splipalmivirus 1	288	288	63%	2.00E-81	35.25	786	QNN89180.1

Supplementary Table 7. On line Blastx results for Contig 2.

Description	Scientific Name	Max Score	Total Score	Query Cover	E value	Per. ident	Acc. Len	Accession
putative RdRp [Monilinia narnavirus H]	Monilinia narnavirus H	640	640	85%	0	37.32	1086	QED42934.1
RNA-dependent RNA polymerase [Erysiphe necator associated narnavirus 22]	Erysiphe necator associated narnavirus 22	612	612	83%	0	38.6	1062	QJT93754.1
RNA-dependent RNA polymerase [Plasmopara viticola lesion associated narnavirus 28]	Plasmopara viticola lesion associated narnavirus 28	466	466	81%	2.00E-141	32.64	1043	QIR30307.1
RNA-dependent RNA polymerase [Plasmopara viticola lesion associated narnavirus 27]	Plasmopara viticola lesion associated narnavirus 27	171	171	64%	8.00E-39	26.24	1101	QIR30306.1
RNA-dependent RNA polymerase [Wenling narna-like virus 6]	Wenling narna-like virus 6	164	237	66%	1.00E-36	27.47	1069	APG77272.1
RNA-dependent RNA polymerase [Erysiphe necator associated narnavirus 34]	Erysiphe necator associated narnavirus 34	140	229	46%	2.00E-29	30.39	1100	QJT93766.1
RNA-dependent RNA polymerase [Grapevine-associated narna-like virus 14]	Grapevine-associated narna-like virus 14	101	101	33%	4.00E-18	25.17	472	QXN75398.1
hypothetical protein [Praha narna-like virus 4]	Praha narna-like virus 4	57.4	57.4	41%	7.00E-04	21.22	1015	QPN36961.1
RNA-dependent RNA polymerase [Beihai narna-like virus 22]	Beihai narna-like virus 22	57	57	23%	9.00E-04	24.43	1027	YP_009333139.1

Supplementary table 8. On line Blastx results for Contig 3.
